# Comparative Genomics Reveals Recurrent Loss of *Autophagy-Related 9B* (*ATG9B*) in Amniotes

**DOI:** 10.3390/genes17060673

**Published:** 2026-06-09

**Authors:** Supawadee Sukseree, Leopold Eckhart

**Affiliations:** Department of Dermatology, Medical University of Vienna, 1090 Vienna, Austria

**Keywords:** autophagy, vesicle, skin, evolution, cetaceans, gene loss, pseudogenization, sebaceous gland, hair, keratinocytes

## Abstract

**Background/Objectives**: Autophagy is an evolutionarily conserved intracellular degradation mechanism that is regulated by a set of autophagy-related (ATG) proteins. The only transmembrane protein among ATGs is the lipid scramblase ATG9, which exists in the form of two paralogs, ATG9A and ATG9B, in humans and other vertebrates. **Methods**: Here, we analyzed human and murine skin transcriptome and proteome datasets for the expression of ATG9 paralogs and performed comparative genomics to determine their conservation during the evolution of amniotes (mammals and sauropsids). **Results**: The expression of *ATG9B*, but not of *ATG9A*, is enriched in differentiated epidermal keratinocytes and in skin appendages of humans and mice. In contrast to the conservation of *ATG9A* in all major clades of amniotes, *ATG9B* has been lost in at least three phylogenetic lineages. Cetaceans, which have unique skin adaptations to aquatic life, harbor mutations that disrupt the open reading frame of *ATG9B*. Many or all species of turtles (*Testudines*) and crocodilians (*Crocodylia*) have entirely lost the *ATG9B* gene. **Conclusions**: *ATG9B* has undergone independent pseudogenization or gene loss in different subgroups of amniotes. In mammalian species that have retained the gene, its expression pattern indicates functions of *ATG9B* in the skin and skin appendages.

## 1. Introduction

Autophagy is an evolutionarily conserved intracellular degradation process that depends on the enclosure of material in double-membraned vesicles and their fusion with lysosomes [1]. A series of autophagy-related (ATG) proteins control the various steps of autophagy [2]. Most of these genes encoding ATG proteins are highly conserved during evolution, which has allowed the use of the yeast *Saccharomyces cerevisiae* to determine the basic principles and genetic regulators of this process [3]. However, gene duplications and, rarely, gene loss, have led to several differences in autophagy regulators in model and non-model species across the phylogenetic tree [4,5,6]. In multicellular organisms such as vertebrates, autophagy is used for multiple purposes, including the control of the metabolism, suppression of toxic reactions, cellular remodeling during differentiation, control of development and aging, and the defense against pathogens [1,7,8,9]. The functions of several components of the molecular machinery of autophagy have remained incompletely understood.

**Figure 1:**
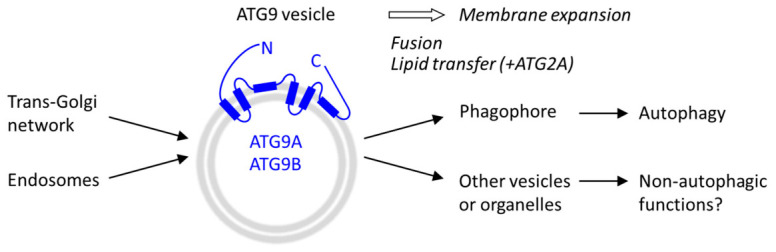
Functions of ATG9 proteins. ATG9A and ATG9B are transmembrane proteins which are localized to vesicles. These so-called ATG9 vesicles transport lipids from either the trans-Golgi network or endosomes to growing phagophores to support the early phase of autophagy. Alternatively, vesicles containing ATG9B may interact with other vesicles or organelles to exert non-autophagic functions. The drawing of the ATG9 vesicle and its suggested links to other cellular structures and functions are based on references [14,15]. In the schematic drawing, the protein is shown by a blue line and boxes for helical segments. The amino-terminus (N) and the carboxy-terminus (C) are labeled. The lipid bilayer of the vesicle is indicated by two gray circles.

ATG9 is the only membrane protein among ATGs in the yeast. It contains four helices that cross the membrane and two helices that enter the membrane [10]. ATG9 forms homotrimers and localizes to single-membrane vesicles, referred to as ATG9 vesicles, where it cooperates with ATG2 in the transfer of lipids to the growing phagophore in the initial phase of autophagy [11]. The function of ATG9 depends on its activity as lipid scramblase as it translocates phospholipids between outer and inner leaflets of vesicles [12,13]. In vertebrates, the ATG9 gene has undergone duplication to give rise to *ATG9A* and *ATG9B* [6]. The topology and functions in phagophore growth are conserved in ATG9A and ATG9B at least in vitro, but additional functions of the two paralogs of ATG9 have been identified [14,15]. Functions unrelated to autophagy include activities at membranes of the Golgi apparatus, lysosomes, and the cell surface. ATG9B has been implicated in interactions with bacteria [16], and the modulation of T-cell functions [17]. The peculiar topology and the functions of ATG9 proteins are schematically depicted in Figure 1.

Despite the common ancestry, ATG9A and ATG9B display considerable differences. ATG9B has a longer amino-terminal segment than ATG9A, whereas its carboxy-terminal segment is shorter than that of ATG9A [10]. Furthermore, ATG9B lacks a region that interacts with ATG13 and ATG101. However, both ATG9A and ATG9B are bound by ATG2A [10]. In contrast to the duplication of the *ATG9* gene in vertebrates, ATG9 and proteins interacting with ATG9 have been lost in ciliates [18,19]. The functional implications of the evolutionary diversification of the *ATG9* gene repertoire are not fully understood.

The evolutionary loss of function of tissues and organs is often associated with the loss or pseudogenization of genes [20,21]. This is particularly well known for adaptations of the skin, such as changes in the barrier function of the epidermis and the loss of skin appendages in cetaceans which are accompanied by the loss of specific genes [22,23]. While this loss is not surprising for genes that are known to be exclusively involved in a particular organ, gene loss events may be informative about the functions of genes that have not or only incompletely been characterized. The identification of “natural gene knockouts” is comparable to the targeted gene knockout in model species, which helps to define links between the genotype and phenotype. The evolutionary loss of autophagy-related genes appears to be a rare event, but many ATG genes have not yet been studied in this regard.

Here, we performed comparative analyses of genome sequences and gene expression datasets to identify differences in the evolutionary conservation and tissue distribution of ATG9A and ATG9B. In particular, we focus on cases of gene loss and pseudogenization events leading to the absence of a functional protein, as defined in a previous paper [21]. We report previously uncharacterized decay of *ATG9B* genes during the evolution of at least three clades of amniotes. Thus, this study identifies natural knockout models which may complement targeted *ATG9B* gene deficiency in experimental model species.

## 2. Materials and Methods

### 2.1. Identification of ATG9 Homologs in Genome Sequences and Assessment of Expression Levels in Tissues

The genome sequences of a phylogenetically diverse set of vertebrates (Table S1) were analyzed for *ATG9A* and *ATG9B* genes. In addition to downloading the sequences of *ATG9* orthologs predicted in GenBank (https://www.ncbi.nlm.nih.gov/gene/, last accessed on 24 March 2026) and ENSEMBL (https://www.ensembl.org/, last accessed on 8 February 2026), the tBLASTn searches (https://blast.ncbi.nlm.nih.gov/Blast.cgi, last accessed 24 March 2026) [24] were performed in the genomic region around the orthologs of the genes that flank *ATG9A* and *ATG9B* in the human genome. The hits were used as queries for further BLAST (versions TBLASTN 2.17.0+ and BLASTP 2.17.0+) searches, and reciprocal best hits were considered orthologous. Default parameters of BLAST at the NCBI GenBank website were used (expect threshold 0.05; word size 5; matrix BLOSUM62; gap costs for existence 11; gap costs for extension 1; conditional compositional score matrix adjustment; filter for low complexity regions). BLASTn searches of the GenBank Sequence Read Archive (SRA) were performed to identify individual sequence reads which confirmed frameshift mutations and/or premature stop codons in *ATG9B* pseudogenes. Nucleotide sequences were translated using the tools on the ExPASy website (https://web.expasy.org/translate/, last accessed on 8 February 2026) [25]. Nucleotide and amino acid sequences were aligned with MUSCLE (MUltiple Sequence Comparison by Log-Expectation) in the version muscle (3.8.425) [26].

The Genotype-Tissue Expression (GTEx) portal [27] and the Protein Atlas database (https://www.proteinatlas.org/, last accessed on 8 February 2026) [28] were used to evaluate expression levels of human genes.

### 2.2. Proteome Analysis

Proteome data from studies of sweat collected from the skin surface (including cellular material detached from the skin) [29], cornified nails [30], and hair [31,32] were analyzed. The criteria for protein identification, such as the detection of at least two unique peptides, and the statistics for quantitative comparisons were obtained from the respective report.

## 3. Results

### 3.1. Analysis of ATG9A and ATG9B Expression Using Transcriptome and Proteome Data of Mammalian Skin

The expression levels of *ATG9A* and *ATG9B* were first compared in human tissues based on the mRNA abundance values available on the GTEx portal [27]. In line with the literature [10], ATG9A has a broader tissue distribution than ATG9B, which is found at highest levels in the skin, esophagus, testis, pituitary gland, vagina, and cervix (Figure S1). The skin contains relatively high levels of both ATG9A and ATG9B mRNA (Figure S1). In the Protein Atlas database [28], ATG9A is part of cluster 25 “Non-specific—Basic cellular processes” (https://www.proteinatlas.org/ENSG00000198925-ATG9A/tissue, last accessed on 8 February 2026), whereas ATG9B is part of the expression cluster 81 “Squamous epithelium—Keratinization” (https://www.proteinatlas.org/ENSG00000181652-ATG9B/tissue, last accessed on 8 February 2026).

Next, we re-analyzed the expression levels of autophagy-related proteins in mammalian skin transcriptome and proteome data available in public repositories. Only data linked to publications in peer-reviewed journals were evaluated with the aim to compare the abundance of ATG9A and ATG9B [29,30,31,32,33,34,35,36]. We found that ATG9B was present both at the mRNA and the protein level in the epithelial compartment (epidermis, skin appendages) of the skin in humans and mice (Table 1). In studies that separated differentiated from undifferentiated epithelial cells, ATG9B was enriched in the differentiated cells (Table 1). According to proteomics, ATG9B protein was present in purified nails and hair shafts [30,31,32], which consist entirely of cornified keratinocytes (Table 1). ATG9A was not consistently detected in the skin transcriptome and proteome data investigated (Table 1), suggesting that its expression level was below the respective threshold of detection in some of the studies [29,30,31,32,33,34,35,36].

### 3.2. Comparative Genomics Indicates Loss of ATG9B in Subgroups of Reptiles and Pseudogenization in Cetaceans

Genes functioning primarily in the skin are predicted to be particularly prone to duplication or loss when the organism adapts to new environments [23,37]. To explore whether skin-associated *ATG9B* (Table 1) shows a different degree of conservation in comparison to ubiquitously expressed *ATG9A* (Table 1), we determined by comparative genomics the presence or absence of both genes in vertebrates that have made the evolutionary transition from fully aquatic life to life on the land (tetrapods), including subclades such as cetaceans that secondarily adopted an aquatic lifestyle. This study utilized genome sequences available in public databases such as GenBank and ENSEMBL.

**Figure 2:**
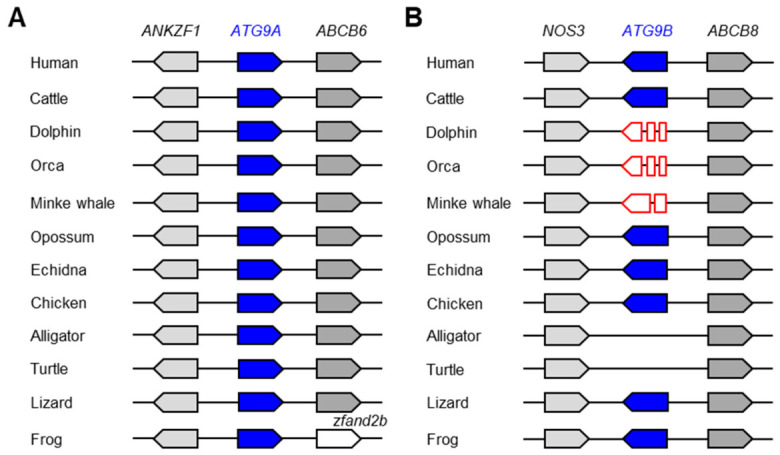
Synteny analysis of *ATG9* genes in tetrapods. The loci of *ATG9A* (**A**) and *ATG9B* (**B**) in relation to the genes flanking them are schematically depicted. Genes are shown as arrows pointing in the direction of transcription. Distances between genes are not drawn to scale. Broken symbols indicate the presence of disruptive mutations in pseudogenes (red). Genes: *ANKZF1*, ankyrin repeat and zinc finger peptidyl tRNA hydrolase 1; *ABCB6*, ATP binding cassette subfamily B member 6; *NOS3*, nitric oxide synthase 3; *ABCB8*, ATP binding cassette subfamily B member 8. Species: Alligator, *Alligator mississippiensis*; cattle, *Bos taurus*; chicken, *Gallus gallus*; dolphin, *Tursiops truncatus*; echidna, *Tachyglossus aculeatus*; frog, *Xenopus tropicalis*; lizard, *Anolis carolinensis*; minke whale, *Balaenoptera acuturostrata*; orca, *Orcinus orca*; opossum, *Monodelphis domestica*; turtle, *Chrysemys picta*.

The arrangements of *ATG9A* and *ATG9B* relative to the neighboring genes was widely conserved in mammals, sauropsids (reptiles and birds), and amphibians (Figure 2 and Figure S2). This conserved synteny allowed us to extend the screening for *ATG9A* and *ATG9B* orthologs beyond the BLAST-dependent detection of similar sequences. In species for which orthologs could not be identified by similarity search, the regions between the conserved neighboring genes, that is, *ANKZF1* (ankyrin repeat and zinc finger peptidyl tRNA hydrolase 1) and *ABCB6* (ATP binding cassette subfamily B member 6) for *ATG9A* and *NOS3* (nitric oxide synthase 3) and *ABCB8* (ATP binding cassette subfamily B member 8) for *ATG9B*, were investigated to substantiate hypotheses about gene loss.

*ATG9A* was conserved in all species for which a genome sequence of the predicted gene locus was available (Figure 2). *ATG9B* was detected in amphibians (anurans, caudates, caecilians), lepidosaurs (geckos, lizards, snakes), birds, and mammals (monotremes, marsupials, and placental mammals) (Figure S2). However, *ATG9B* appeared to be pseudogenized in cetaceans (whales, dolphins, and porpoises; for details, see the next section). No *ATG9B* orthologs are present in two clades of reptiles, namely turtles (*Testudines*) and crocodilians (*Crocodylia*). In these clades, all species for which high-quality annotated genome sequences were available, including the green sea turtle (*Chelonia mydas*), the Western painted turtle (*Chrysemys picta bellii*), the American (*Alligator mississippiensis*) and the Chinese alligator (*Alligator sinensis*), contained no gene at the evolutionarily ancient locus between *NOS3* and *ABCB8* (Figure 2). In the genomes of the Western painted turtle and the American alligator, the distance between *NOS3* and *ABCB8* is smaller than 1500 nucleotides (Figure S3), which is too short for a gene encoding a protein of more than 750 amino acid residues, such as ATG9B.

In a complementary screening approach, we used chicken ATG9B (GenBank accession number XP_040521369.1) and anole lizard ATG9B (GenBank accession number XP_062814233.1) as queries in tBLASTn searches of whole genome shotgun sequences (WGS), including unplaced scaffolds of turtles and crocodilians available in GenBank (last accessed on February 28, 2026). Among the genome sequences investigated was the genome (GenBank accession number GCA_045364815.1) of the Arrau turtle (*Podocnemis expansa*), a side-neck turtle (*Pleurodira*) which represents the phylogenetically basal clade of extant turtles. Furthermore, genome sequences of the gharial (*Gavialis gangeticus*), genome assembly ggan_v0.2, and the Australian saltwater crocodile (*Crocodylus porosus*), genome assembly CroPor_comp1, were investigated. The gharial and the saltwater crocodile belong to the superfamilies *Gavialoidea* and *Crocodyloidea*, which together constitute *Longirostres*, the sister clade of *Alligatoroidea* [38]. In both turtles and crocodylians, the best tBLASTn hits corresponded to homologs of *ATG9A*, and no ortholog of *ATG9B* was identified. Thus, turtles and crocodylians lack *ATG9B* (Figure 2).

### 3.3. ATG9B of Toothed Whales Harbors Disruptive Mutations

**Figure 3:**
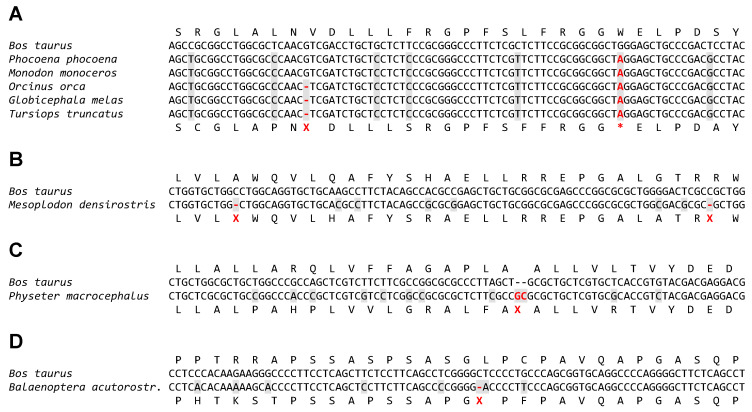
The *ATG9B* gene is disrupted by mutations in cetaceans. Nucleotide sequences of different segments of exon 6 (**A**–**C**) and exon 1 (**D**) of *ATG9B* of the cattle (*Bos taurus*) were aligned to the homologous sequences of cetaceans. Disruptive mutations in *Delphinoidea* (**A**), Blainville’s beaked whale (*Mesoplodon densirostris*) (**B**), sperm whale (*Physeter macrocephalus*), (**C**) and minke whale (*Balaenoptera acuturostrata*) (**D**) are shown in red fonts. Gray shading indicates differences from the sequence of cattle *ATG9B*. Amino acid sequences are shown above and below the nucleotide sequences. Additional data are shown in Figure S5. Species: bottlenose dolphin, *Tursiops truncatus*; harbor porpoise, *Phocoena phocoena*; long-finned pilot whale, *Globicephala melas*; narwhal, *Monodon monoceros*; orca, *Orcinus orca*. X, frameshift; *, in-frame stop codon.

Because the initial screening of mammalian genomes indicated mutations in the *ATG9B* genes of cetaceans, the genome sequences of more than 20 species of this clade were investigated in more detail (Table S1). Given that ATG9B was detected at high levels in the skin and skin appendages, cetaceans are of particular interest because they have a histologically modified and thickened epidermis and lack hair, nails, sebaceous glands, and sweat glands. In toothed whales (*Odontoceti*), multiple frameshift mutations or premature stop codons, together referred to as “disruptive mutations”, were present in the *ATG9B* genes of all species investigated (Figure 3 and Figure S4). Mutations at different sites of the *ATG9B* gene affected members of the clade *Delphinoidea*, including dolphins, the orca and porpoises (Figure 3A), a beaked whale (Figure 3B), and the sperm whale (Figure 3C). At least five disruptive mutations were present in the *ATG9B* pseudogenes of representative species of *Odontoceti*, such as the common bottlenose dolphin and the pygmy sperm whale (Figure S4). Some of the disruptive mutations were shared among *Delphinoidea*, indicating that they had been inherited from a common ancestor (Figure 3A and Figure S5). Taken together, these data demonstrate that *ATG9B* has undergone pseudogenization in toothed whales.

In baleen whales (*Mysticeti*), the *ATG9B* genes contain point mutations impairing the expression of the gene as a functional protein in five species investigated. The first exons of *Balaenoptera acutorostrata* (Figure 3D), *Balaenoptera ricei*, and *Eubalaena glacialis* (Figure S6) harbor frameshift mutations at different positions. In the blue whale (*Balaenoptera musculus*), a premature in-frame stop codon is present in the last exon (Figure S7), and the splice acceptor site at the end of the last intron is mutated in the gray whale (*Eschrichtius robustus*) (Figure S8).

**Figure 4:**
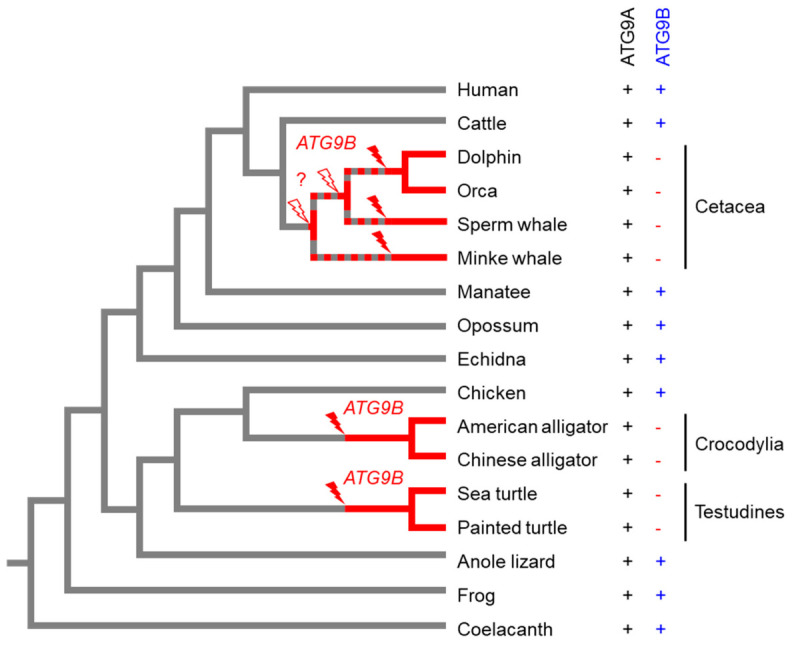
Evolution of *ATG9A* and *ATG9B* in tetrapods. The presence (+) or absence (-) of functional *ATG9A* and *ATG9B* genes were mapped onto a phylogenetic tree of species investigated in this study. Gene loss and pseudogenization events are marked by flash symbols. Two alternative scenarios are possible for the evolution of *ATG9B* in cetaceans: parallel pseudogenization in subclades of cetaceans (red filled flash symbols) or pseudogenization in a common ancestor (white flash symbols with red frames). The coelacanth (*Latimeria chalumnae*) is included to indicate the presence of both *ATG9A* and *ATG9B* in the basal group of lobe-finned fish (*Sarcopterygii*).

The species distribution of *ATG9A* and *ATG9B* was integrated into the phylogenetic tree of vertebrates to infer the evolutionary history of the two genes in different lineages. *ATG9B* was lost or pseudogenized in parallel during the evolution of cetaceans (*Cetacea*), crocodylians (*Crocodylia*), and turtles (*Testudines*) (Figure 4).

## 4. Discussion

This study shows that, in contrast to most other *ATG* genes [6], *ATG9B* has not been strictly conserved in evolution. Obviously, *ATG9B* has arisen through the duplication of an ancient *ATG9* gene, which was probably functionally equivalent to the other product of the gene duplication event, *ATG9A* [6,10]. The time of the duplication predated the divergence of jawless vertebrates (*Cyclostomata*) and jawed vertebrates (*Gnathostomata*) [6]. Further studies are required to determine the evolution and functions of ATG9 paralogs in fishes. ATG9B is able to compensate ATG9A in some contexts [10] but not in others [39,40]. Both ATG9A and ATG9B have been demonstrated to mediate the transfer of lipids [10], clearly distinguishing them from all other ATG proteins. However, the two proteins differ in their structure and, most importantly, the expression patterns of the *ATG9A* and *ATG9B* genes are different. Thus, the loss of *ATG9B* is not primarily a decrease in the total abundance of ATG9 protein, but rather a loss of ATG9B-mediated lipid transport functions in specific tissues.

Our analysis of gene expression data showed that *ATG9B* is not only expressed at high levels in the placenta and pituitary gland, as was emphasized in previous reports [6,10], but also in the esophagus and the skin. In various studies, *ATG9B* mRNA and protein were detected in the skin and skin appendages [30,31,32,33,34,35,36]. Although the expression level of ATG9A appears to be lower than that of ATG9B in some skin compartments (Table 1), it plays an important role in the skin. The deletion of *Atg9a* in epidermal keratinocytes caused spontaneous skin inflammation in mice [39,40], suggesting that ATG9A plays an anti-inflammatory role in the skin. In this model, ATG9B did not compensate for the loss of ATG9A [39,40]. Proteomic evidence for the presence of ATG9B protein in nails and hair (Table 1) indicates expression during the terminal differentiation of keratinocytes, which build these skin appendages upon cornification [41]. Other data shown in Table 1 support the hypothesis of keratinocyte differentiation-associated expression of ATG9B, but further localization of ATG9B expression is required for sebaceous glands and the interfollicular epidermis.

It is important to note that not only autophagy [42,43,44,45,46], but also other vesicle-dependent processes are active in skin epithelial cells. Most notably, the production of sebum is equivalent to the accumulation of lipid within epithelial cells followed by cell disintegration [47,48]. LacZ reporter expression data of the International Mouse Phenotyping Consortium (IMPC; https://www.mousephenotype.org/, last accessed on 27 February 2026) [49] indicate that expression of *Atg9b* is active in the sebaceous glands of the skin (https://www.mousephenotype.org/data/genes/MGI:2685420/images/IMPC_ALZ_075_001?anatomyTerm=skin, last accessed on 27 February 2026) and a special type of sebaceous glands known as preputial glands (https://www.kompphenotype.org/lacz-tab.php?gene=Atg9b&project=komp, last accessed on 27 February 2026). In the mammalian epidermis [41,50], so-called lamellar bodies discharge lipids into the extracellular space between cornified keratinocytes to establish the skin barrier against the environment. Autophagy-related genes are implicated in epithelial differentiation in the epidermis and in epithelia of other organs [51,52], but the role of ATG9B remains to be determined in suitable models.

While studies of *Atg9b* knockout mice have not been reported yet, our comparative genomics study identified cases of inactivation of *ATG9B* during the evolution of amniotes. The loss of *ATG9B* in turtles and crocodilians and its conservation in other reptiles and birds point to differences in the requirement for *ATG9B* among the clades of sauropsids. Due to incomplete phylogenetic sampling and the current lack of high-quality genome sequence assemblies of species in the clades *Pleurodira*, *Gavialoidea*, and *Crocodyloidea*, it is presently not possible to perform a comprehensive investigation of all subgroups of turtles and crocodilians. However, the genome sequences of the Western painted turtle (*Chrysemys picta bellii*) and the American alligator (*Alligator mississippiensis*) showed unambiguously that *ATG9B* is absent from its evolutionarily ancient locus (Figure S3), and extensive BLAST searches of the respective genome sequences, including unassembled contigs, did not reveal an *ATG9B* at another locus in these species. The scarcity of gene expression data in relevant tissues makes it difficult to build hypotheses about the roles of ATG9B in sauropsids and the potential drivers and consequences of *ATG9B* loss in turtles and crocodilians. Notably, the expression of *ATG9B* was upregulated during the differentiation of chicken keratinocytes in vitro [53] suggesting that chicken *ATG9B* may be expressed in a similar way as its mammalian ortholog (Table 1).

Cetaceans have a unique skin among mammals. They lack pilosebaceous units (hair and sebaceous glands), nails and sweat glands, and the thickness of the epidermis is increased relative to their land-dwelling phylogenetic relatives [54]. In particular, the intracellular distribution of lipids within epidermal keratinocytes differs between cetaceans and other mammals [55]. The availability of more than twenty genomes of cetaceans facilitated an in-depth analysis of *ATG9B* genes in this clade. The annotations of the genes in GenBank and ENSEMBL appeared largely, but not completely, reliable. Some errors in the prediction of exon borders affected exons 2 and 3, which are separated by an intron of the rare AT-AC type [56,57], in which the common splice donor (GT) and acceptor (AG) motifs are not present (Figure S8). The homologous intron within *ATG9A* belongs to the same type, indicating an ancient origin. The identification of multiple frameshift mutations of *ATG9B*, some of which are present in several independently sequenced genomes (Figure 3 and Figure S5), demonstrates the pseudogenization of *ATG9B* in toothed whales. Of note, the narwhal (*Monodon monoceros*) was previously reported to have an *ATG9B* gene [10], but the present study shows that this gene contains inactivating mutations (Figure 3A). In baleen whales, *ATG9B* genes contain single point mutations that either disrupt the coding sequence (Figure 3D, Figures S6 and S7) or impair normal splicing (Figure S8). Although errors in the genome sequence assemblies or the production of truncated ATG9B proteins cannot be excluded, it appears likely that some or all species of baleen whales have lost functional ATG9B. As only a few positions of mutations within *ATG9B* are shared among species of the clade *Delphinoidea* (Figure 3), but not in beaked whales, sperm whales, and baleen whales, pseudogenization of *ATG9B* has probably occurred in parallel in different phylogenetic lineages of cetaceans (Figure 4). However, it is possible that one or more mutations not identified in the present study have led to *ATG9B* pseudogenization already in the last common ancestor of *Cetacea* or the last common ancestor of *Odontoceti* (Figure 4, scenario indicated by white flash symbols). Interestingly, *ATG9B* has remained intact during the evolution of the manatee (Figure 4), which is a fully aquatic mammal with skin appendages in the form of vibrissae [58].

With regard to the evolution of *ATG9B* in mammals, we put forward the hypothesis that *ATG9B* became dispensable upon the evolutionary degeneration of several skin features in cetaceans [21,22,23], whereas *ATG9B* was retained in other species in which it contributes to the functions of the skin. However, alternative selective pressures unrelated to skin are not excluded. The roles of *ATG9B* in mammalian skin and other organs remain to be investigated by targeted gene deletion studies in the mouse or other model species. Insights from these studies might also help to evaluate the pathophysiological roles of *ATG9B* that were proposed in the literature [15,16,17,59,60,61,62,63,64,65,66,67].

## 5. Conclusions

In conclusion, this study reveals that *ATG9A* is evolutionarily conserved, whereas *ATG9B* has been lost in at least three subclades of amniotes. In these phylogenetic lineages, either the loss of *ATG9B* is compensated by *ATG9A*, or the process controlled by *ATG9B*, which may be a subtype of autophagy or a non-autophagic process depending on vesicles, is not required. The expression of *ATG9B* in human and murine tissues points to an important role of *ATG9B* in the skin and skin appendages. However, the precise roles of ATG9B in the skin cells of humans and other species are presently unknown. Further studies are required to characterize the functions of ATG9 paralogs in tissues and to determine which phenotypic changes are linked to the evolutionary loss of *ATG9B* in turtles, crocodilians, and cetaceans.

## Figures and Tables

**Table 1:** Identification of *ATG9A* and *ATG9B* gene products in published transcriptome and proteome data of mammalian skin.

Tissue	Species	Method (Molecule)	ATG9A	ATG9B	Notes	Reference
Epidermis, granular layer	Mouse	RNA-seq (mRNA)	+	+	ATG9B is enriched in differentiated keratinocytes	[33]
Epidermis, upper layers	Human	Micro-array (mRNA)	−	+	ATG9B is enriched in differentiated keratinocytes	[35]
Nails	Mouse	Proteomics (protein)	−	+	−	[30]
Hair	Mouse	Proteomics (protein)	−	+	−	[32]
Hair	Human	Proteomics (protein)	−	+	−	[31]
Sweat, skin surface	Human	Proteomics (protein)	−	+	−	[29]
Sebaceous glands	Human	Spatial transcriptomics (mRNA)	+	+	ATG9B is enriched in differentiated sebocytes	[36]
Hair follicles, sebaceous glands	Human	RNA-seq (mRNA)	−	+	ATG9B is downregulated in alopecia	[34]

## Data Availability

Genome sequence data were obtained from NCBI GenBank. Transcriptome and proteome data were obtained from published reports and datasets described in these publications which are cited in this manuscript.

## Supplementary Materials

The following supporting information can be downloaded at: https://www.mdpi.com/article/10.3390/genes17060673/s1, Figure S1: Expression levels of *ATG9A* and *ATG9B* in human tissues; Figure S2: *ATG9B* genes and proteins of tetrapods; Figure S3: Absence of *ATG9B* between *NOS3* and *ABCB8* in the Western painted turtle and the American alligator. turtles and crocs; Figure S4: Multiple disruptive mutations are present in the *ATG9B* pseudogenes of toothed whales (*Odontoceti*); Figure S5: Nucleotide sequence alignment of *ATG9B* sequence segments of cetaceans; Figure S6: Alignment of nucleotide sequences from exon 1 of *ATG9B* of baleen whales; Figure S7: *ATG9B* of the blue whale (*B. musculus*) contains a premature stop codon in the last exon; Figure S8: *ATG9B* of the gray whale (*E. robustus*); Table S1: Accession numbers of genome sequences.
