# Supplementary material for: Comparative Genomics Reveals Recurrent Loss of Autophagy-Related 9B (ATG9B) in Amniotes
Source: Genes (Basel). 2026 Jun 9;17(6):673. doi: 10.3390/genes17060673 (PMC13299143; doi:10.3390/genes17060673)
Supplement: Supplementary file 1 [file genes-17-00673-s001.zip › Supplement.pdf]

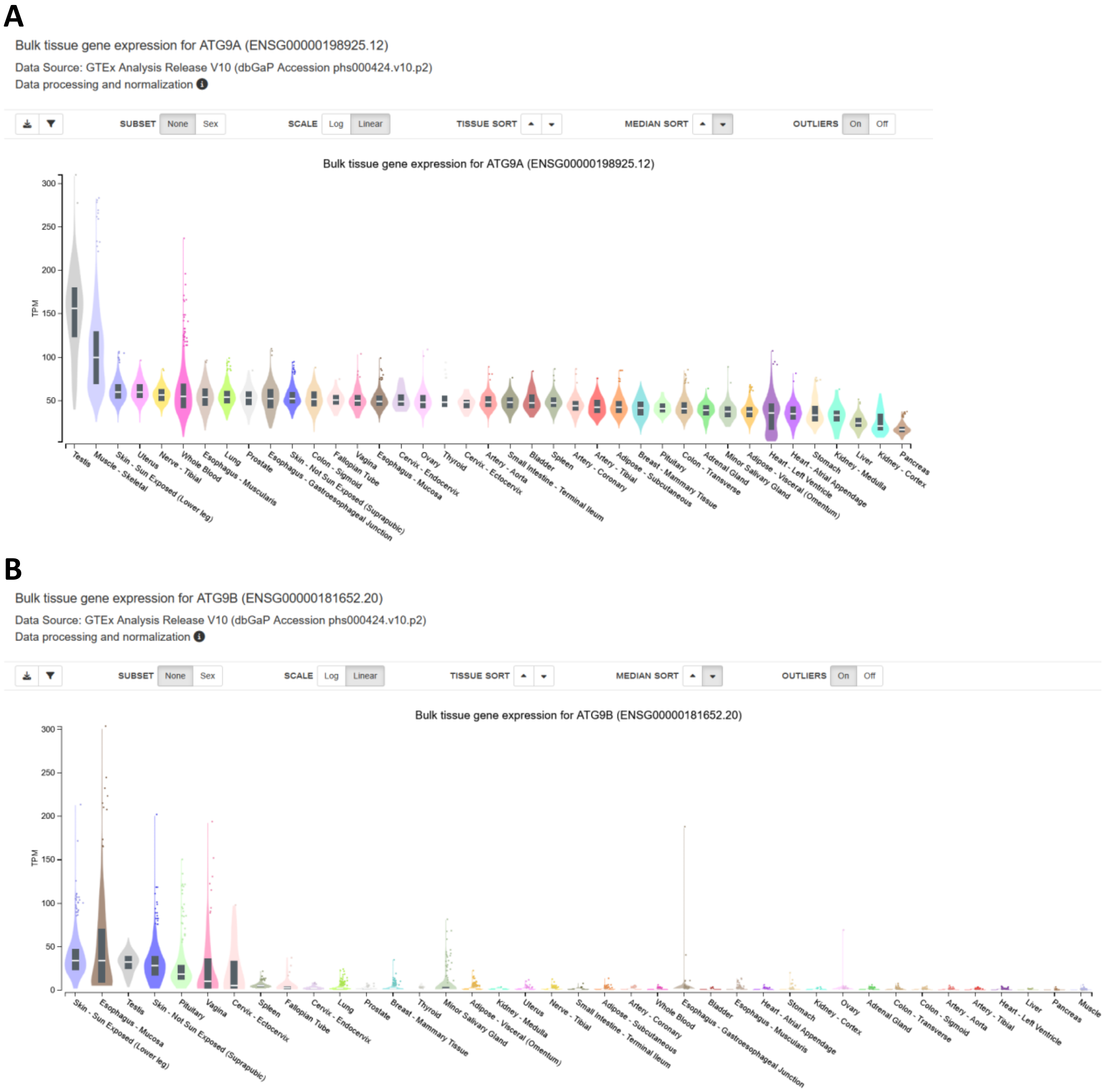

Figure S1. Expression levels of *ATG9A* and *ATG9B* in human tissues. The data used for this analysis and the images were obtained from the GTEx Portal, https://www.gtexportal.org/home/gene/ATG9B/geneExpressionTab, last accessed on 8 February 2026 (GTEx Consortium 2013). Data Source: GTEx Analysis Release V10 (dbGaP Accession phs000424.v10.p2). TPM, transcripts per million.

(A) Tropical clawed frog

```
LOCUS       NC_030682              16212 bp    DNA     linear   CON 18-DEC-2019
DEFINITION  Xenopus tropicalis strain Nigerian chromosome 6, UCB_Xtro_10.0,
            whole genome shotgun sequence.
ACCESSION   NC_030682 REGION: 1408653..1424864
VERSION     NC_030682.2
DBLINK      BioProject: PRJNA205740
            BioSample: SAMN13041969
            Assembly: GCF_000004195.4
KEYWORDS    WGS; RefSeq.
SOURCE      Xenopus tropicalis (tropical clawed frog)
  ORGANISM  Xenopus tropicalis
            Eukaryota; Metazoa; Chordata; Craniata; Vertebrata; Euteleostomi;
            Amphibia; Batrachia; Anura; Pipoidea; Pipidae; Xenopodinae;
            Xenopus; Silurana.
REFERENCE   1  (bases 1 to 16212)
  AUTHORS   Hellsten,U., Harland,R.M., Gilchrist,M.J., Hendrix,D., Jurka,J.,
            Kapitonov,V., Ovcharenko,I., Putnam,N.H., Shu,S., Taher,L.,
            Blitz,I.L., Blumberg,B., Dichmann,D.S., Dubchak,I., Amaya,E.,
            Detter,J.C., Fletcher,R., Gerhard,D.S., Goodstein,D., Graves,T.,
            Grigoriev,I.V., Grimwood,J., Kawashima,T., Lindquist,E.,
            Lucas,S.M., Mead,P.E., Mitros,T., Ogino,H., Ohta,Y., Poliakov,A.V.,
            Pollet,N., Robert,J., Salamov,A., Sater,A.K., Schmutz,J., Terry,A.,
            Vize,P.D., Warren,W.C., Wells,D., Wills,A., Wilson,R.K.,
            Zimmerman,L.B., Zorn,A.M., Grainger,R., Grammer,T., Khokha,M.K.,
            Richardson,P.M. and Rokhsar,D.S.
  TITLE     The genome of the Western clawed frog Xenopus tropicalis
  JOURNAL   Science 328 (5978), 633-636 (2010)
   PUBMED   20431018
COMMENT     REFSEQ INFORMATION: The reference sequence is identical to
            CM004448.2.

            On Dec 18, 2019 this sequence version replaced NC_030682.1.
            Assembly name: UCB_Xtro_10.0
            The genomic sequence for this RefSeq record is from the
            whole-genome assembly released by the University of California,
            Berkeley on 2019/11/14. The original whole-genome shotgun project
            has the accession AAMC00000000.4.

            ##Genome-Assembly-Data-START##
            Assembly Provider      :: University of California, Berkeley
            Assembly Date          :: APR-2019
            Assembly Method        :: Supernova v. 1.1.5; Canu v.
                                      1.6-132-gf9284f8; DBG2OLC v. commit
                                      1f7e752; 3D-DNA v. commit 2796c3b;
                                      quickmerge v. commit e4ea490
            Assembly Name          :: UCB_Xtro_10.0
            Genome Representation  :: Full
            Expected Final Version :: Yes
            Genome Coverage        :: 111.5x
            Sequencing Technology  :: PacBio Sequel; Illumina HiSeq
            ##Genome-Assembly-Data-END##

            ##Genome-Annotation-Data-START##
            Annotation Provider         :: NCBI
            Annotation Status           :: Full annotation
            Annotation Name             :: Xenopus tropicalis Annotation
                                           Release 104
            Annotation Version          :: 104
            Annotation Pipeline         :: NCBI eukaryotic genome annotation
                                           pipeline
            Annotation Software Version :: 8.3
            Annotation Method           :: Best-placed RefSeq; Gnomon
            Features Annotated          :: Gene; mRNA; CDS; ncRNA
            ##Genome-Annotation-Data-END##
FEATURES             Location/Qualifiers
     source          1..16212
                     /organism="Xenopus tropicalis"
                     /mol_type="genomic DNA"
                     /strain="Nigerian"
                     /db_xref="taxon:8364"
                     /chromosome="6"
                     /sex="female"
                     /tissue_type="liver and blood"
                     /dev_stage="adult"
                     /note="F17 inbred"
     gene            1..16212
```

```
                     /gene="atg9b"
                     /note="Derived by automated computational analysis using
                     gene prediction method: Gnomon."
                     /db_xref="GeneID:100486160"
                     /db_xref="Xenbase:XB-GENE-6257562"
     mRNA            join(1..1050,6915..7061,7267..7310,7834..7898,8598..8765,
                     8859..9000,9261..10009,10608..10761,10897..11076,
                     11535..11780,11993..12207,12370..12475,12632..12716,
                     12870..13035,13169..16212)
                     /gene="atg9b"
                     /product="autophagy related 9B, transcript variant X2"
                     /note="Derived by automated computational analysis using
                     gene prediction method: Gnomon. Supporting evidence
                     includes similarity to: 18 ESTs, 14 Proteins, and 100%
                     coverage of the annotated genomic feature by RNAseq
                     alignments, including 44 samples with support for all
                     annotated introns"
                     /transcript_id="XM_018094712.2"
                     /db_xref="GeneID:100486160"
                     /db_xref="Xenbase:XB-GENE-6257562"
     mRNA            join(4..1050,6915..7061,7267..7310,7834..7898,8598..8765,
                     8859..9000,9261..10009,10608..10761,10897..11076,
                     11535..11780,11993..12207,12370..12475,12632..12716,
                     12870..13035,13200..16212)
                     /gene="atg9b"
                     /product="autophagy related 9B, transcript variant X1"
                     /note="Derived by automated computational analysis using
                     gene prediction method: Gnomon. Supporting evidence
                     includes similarity to: 18 ESTs, 14 Proteins, and 100%
                     coverage of the annotated genomic feature by RNAseq
                     alignments, including 113 samples with support for all
                     annotated introns"
                     /transcript_id="XM_002943013.5"
                     /db_xref="GeneID:100486160"
                     /db_xref="Xenbase:XB-GENE-6257562"
     CDS             join(6953..7061,7267..7310,7834..7898,8598..8765,
                     8859..9000,9261..10009,10608..10761,10897..11076,
                     11535..11780,11993..12207,12370..12475,12632..12716,
                     12870..13015)
                     /gene="atg9b"
                     /note="Derived by automated computational analysis using
                     gene prediction method: Gnomon."
                     /codon_start=1
                     /product="autophagy-related protein 9B"
                     /protein_id="XP_017950201.1"
                     /db_xref="GeneID:100486160"
                     /db_xref="Xenbase:XB-GENE-6257562"
                     /translation="MEEQEQFREYQRLEDFEEDSPPGEDDPLVHVPDAVKDSWHHIKN
                     LDNFFTKIYHFHQKNGFACMVLSDFFELIQFLFVVTFTTFLFHCVEYDVLFANKPVNH
                     THSGGAPERNKVTLADAILPAQQCALRIQESGWIIFLLVMAAIFWLYRLIKMFCSLLS
                     YWEIRRFYIRALKIPSDELCNFSWQEVQSRLISLQREQPMCVHKKELTELDIYHRILR
                     FKNYMVAMVNKSLLPVRFRLPLIGEATFLTQGLKYNMEFLFFWGPGSLFQNKWNLQPK
                     YKRLGQRLELAQELNRTIVLLGLANLFLCPFILVWQILYAFFSYTEVIKREPGSLGAR
                     RWSLYGRLYLRHFNELTHELQARLSRGYKPATKYMNSFASPLLAIAAKNIAFFAGSLL
                     AVLIALTVYDEDVLTVQHILTAITVLGVMVTVARSFIPDEHMVWCPEQLLQCVLAHIH
                     YMPDHWQGHAHKSETRDELAQLFQYKAVFILEELLSPIITPFILIFSLRHKSLEIIDF
                     FRNFSVEVVGVGDICSFAQMDIRRHGNPQWLSEGQTQASVYQQAENGKTELSLMHFAI
                     TNPHWQPPLESSVFIGHLKEKVQQDAANAPPAQQILCDAPICSSFLSNESGTAPDNLL
                     ASVLVHPVLTASGLPTRDRRFAQPTTTASAAASVLASLSGSQFSGRHLGANAHSSMYR
                     SDQTLQQESMSQSDSQQKNMSRSSLFASEFASAEMSLHAIYMHEVHQNQGITQRPLGM
                     WQASELSPHQRQQTATRSGSRSGYRGLQLGGWEEEEQLHKVDERSPPQAPSVGPSTSG
                     SNSS"
     CDS             join(6953..7061,7267..7310,7834..7898,8598..8765,
                     8859..9000,9261..10009,10608..10761,10897..11076,
                     11535..11780,11993..12207,12370..12475,12632..12716,
                     12870..13015)
                     /gene="atg9b"
                     /note="Derived by automated computational analysis using
                     gene prediction method: Gnomon."
                     /codon_start=1
                     /product="autophagy-related protein 9B"
                     /protein_id="XP_002943059.2"
                     /db_xref="GeneID:100486160"
                     /db_xref="Xenbase:XB-GENE-6257562"
                     /translation="MEEQEQFREYQRLEDFEEDSPPGEDDPLVHVPDAVKDSWHHIKN
                     LDNFFTKIYHFHQKNGFACMVLSDFFELIQFLFVVTFTTFLFHCVEYDVLFANKPVNH
                     THSGGAPERNKVTLADAILPAQQCALRIQESGWIIFLLVMAAIFWLYRLIKMFCSLLS
                     YWEIRRFYIRALKIPSDELCNFSWQEVQSRLISLQREQPMCVHKKELTELDIYHRILR
```

```
                    FKNYMVAMVNKSLLPVRFRLPLIGEATFLTQGLKYNMEFLFFWGPGSLFQNKWNLQPK
                    YKRLGQRLELAQELNRTIVLLGLANLFLCPFILVWQILYAFFSYTEVIKREPGSLGAR
                    RWSLYGRLYLRHFNELTHELQARLSRGYKPATKYMNSFASPLLAIAAKNIAFFAGSLL
                    AVLIALTVYDEDVLTVQHILTAITVLGVMVTVARSFIPDEHMVWCPEQLLQCVLAHIH
                    YMPDHWQGHAHKSETRDELAQLFQYKAVFILEELLSPIITPFILIFSLRHKSLEIIDF
                    FRNFSVEVVGVGDICSFAQMDIRRHGNPQWLSEGQTQASVYQQAENGKTELSLMHFAI
                    TNPHWQPPLESSVFIGHLKEKVQQDAANAPPAQQILCDAPICSSFLSNESGTAPDNLL
                    ASVLVHPVLTASGLPTRDRRFAQPTTTASAAASVLASLSGSQFSGRHLGANAHSSMYR
                    SDQTLQQESMSQSDSQQKNMSRSSLFASEFASAEMSLHAIYMHEVHQNQGITQRPLGM
                    WQASELSPHQRQQTATRSGSRSGYRGLQLGGWEEEEQLHKVDERSPPQAPSVGPSTSG
                    SNSS"
```

(B) Axolotl

```
LOCUS       NC_090923             446942 bp    DNA     linear   CON 15-NOV-2024
DEFINITION  Ambystoma mexicanum isolate Mex_15411 chromosome 2p, UKY_AmexF1_1,
            whole genome shotgun sequence.
ACCESSION   NC_090923 REGION: complement(1434487455..1434934396)
VERSION     NC_090923.1
DBLINK      BioProject: PRJNA1165261
            BioSample: SAMN43142723
            Assembly: GCF_040938575.1
KEYWORDS    WGS; RefSeq.
SOURCE      Ambystoma mexicanum (axolotl)
  ORGANISM  Ambystoma mexicanum
            Eukaryota; Metazoa; Chordata; Craniata; Vertebrata; Euteleostomi;
            Amphibia; Batrachia; Caudata; Salamandroidea; Ambystomatidae;
            Ambystoma.
REFERENCE   1  (bases 1 to 446942)
  AUTHORS   Smith,J.J., Timoshevskaya,N., Timoshevskiy,V.A., Keinath,M.C.,
            Hardy,D. and Voss,S.R.
  TITLE     A chromosome-scale assembly of the axolotl genome
  JOURNAL   Genome Res 29 (2), 317-324 (2019)
   PUBMED   30679309
COMMENT     REFSEQ INFORMATION: The reference sequence is identical to
            CM082195.1.
            Assembly name: UKY_AmexF1_1
            The genomic sequence for this RefSeq record is from the
            whole-genome assembly released by the University of Kentucky on
            2024/07/30. The original whole-genome shotgun project has the
            accession JBEBLI000000000.1.

            ##Genome-Assembly-Data-START##
            Assembly Provider      :: University of Kentucky
            Assembly Date          :: JAN-2024
            Assembly Method        :: HiFiasm, YAHS, PreTextView v. JAN-2024
            Assembly Name          :: UKY_AmexF1_1
            Genome Representation  :: Full
            Expected Final Version :: Yes
            Genome Coverage        :: 48.0x
            Sequencing Technology  :: PacBio Sequel; Illumina HiSeq
            ##Genome-Assembly-Data-END##

            ##Genome-Annotation-Data-START##
            Annotation Provider         :: NCBI RefSeq
            Annotation Status           :: Full annotation
            Annotation Name             :: GCF_040938575.1-RS_2024_10
            Annotation Pipeline         :: NCBI eukaryotic genome annotation
                                           pipeline
            Annotation Software Version :: 10.3
            Annotation Method           :: Gnomon; cmsearch; tRNAscan-SE
            Features Annotated          :: Gene; mRNA; CDS; ncRNA
            Annotation Date             :: 10/03/2024
            ##Genome-Annotation-Data-END##
FEATURES             Location/Qualifiers
     source          1..446942
                     /organism="Ambystoma mexicanum"
                     /mol_type="genomic DNA"
                     /isolate="Mex_15411"
                     /db_xref="taxon:8296"
                     /chromosome="2p"
                     /sex="female"
                     /tissue_type="tail tip"
                     /geo_loc_name="USA: Kentucky, University of Kentucky"
                     /collection_date="2023-02"
     gene            1..446942
                     /gene="LOC138567588"
                     /note="autophagy-related protein 9A-like; Derived by
```

```
                     automated computational analysis using gene prediction
                     method: Gnomon."
                     /db_xref="GeneID:138567588"
     mRNA            join(1..168,29587..29642,148463..148610,168715..168758,
                     169973..170037,174977..175144,217744..217885,
                     236402..237150,279236..279389,291273..291452,
                     310218..310460,334041..334273,343357..343462,
                     375712..375799,397624..397808,444914..446942)
                     /gene="LOC138567588"
                     /product="autophagy-related protein 9A-like, transcript
                     variant X3"
                     /experiment="COORDINATES: polyA evidence [ECO:0006239]"
                     /transcript_id="XM_069645965.1"
                     /db_xref="GeneID:138567588"
     mRNA            join(50..168,148463..148610,168715..168760,169975..170037,
                     174977..175144,217744..217885,236402..237150,
                     279236..279389,291273..291452,310218..310460,
                     334041..334273,343357..343462,375712..375799,
                     397624..397808,444914..446942)
                     /gene="LOC138567588"
                     /product="autophagy-related protein 9A-like, transcript
                     variant X2"
                     /experiment="COORDINATES: polyA evidence [ECO:0006239]"
                     /transcript_id="XM_069645964.1"
                     /db_xref="GeneID:138567588"
     mRNA            join(50..168,28608..28759,29587..29642,148463..148610,
                     168715..168758,169973..170037,174977..175144,
                     217744..217885,236402..237150,279236..279389,
                     291273..291452,310218..310460,334041..334273,
                     343357..343462,375712..375799,397624..397808,
                     444914..446942)
                     /gene="LOC138567588"
                     /product="autophagy-related protein 9A-like, transcript
                     variant X4"
                     /experiment="COORDINATES: polyA evidence [ECO:0006239]"
                     /transcript_id="XM_069645966.1"
                     /db_xref="GeneID:138567588"
     mRNA            join(52..168,28605..28759,29587..29642,148463..148610,
                     168715..168760,169975..170037,174977..175144,
                     217744..217885,236402..237150,279236..279389,
                     291273..291452,310218..310460,334041..334273,
                     343357..343462,375712..375799,397624..397808,
                     444914..446942)
                     /gene="LOC138567588"
                     /product="autophagy-related protein 9A-like, transcript
                     variant X1"
                     /experiment="COORDINATES: polyA evidence [ECO:0006239]"
                     /transcript_id="XM_069645963.1"
                     /db_xref="GeneID:138567588"
     mRNA            join(148494..148618,168715..168760,169975..170037,
                     174977..175144,217744..217885,236402..237150,
                     279236..279389,291273..291452,310218..310460,
                     334041..334273,343357..343462,375712..375799,
                     397624..397808,444914..446942)
                     /gene="LOC138567588"
                     /product="autophagy-related protein 9A-like, transcript
                     variant X5"
                     /experiment="COORDINATES: polyA evidence [ECO:0006239]"
                     /transcript_id="XM_069645967.1"
                     /db_xref="GeneID:138567588"
     CDS             join(148502..148610,168715..168758,169973..170037,
                     174977..175144,217744..217885,236402..237150,
                     279236..279389,291273..291452,310218..310460,
                     334041..334273,343357..343462,375712..375799,
                     397624..397787)
                     /gene="LOC138567588"
                     /note="Derived by automated computational analysis using
                     gene prediction method: Gnomon."
                     /codon_start=1
                     /product="autophagy-related protein 9A-like isoform X2"
                     /protein_id="XP_069502066.1"
                     /db_xref="GeneID:138567588"
                     /translation="MSGREEDREYQRLEDFEEDSPPGEEDLLVHVPEGLKDSWHHIKN
                     LDNFFTKIYNFHQKNGFACMVLSDFFEMVQFVFVVTFTTFLFCCVEYDILFANKPVNH
                     SHSATSPESYKVTLTDAILPSPQCAKRIQDDSWIIFLLAMAAVFWLYRLVKMFCNLLS
                     YWEIRSFYIRALKIPSDELCNFTWQEVQDRLVSLQREQQMCVHKKELTELDIYHRILR
                     FKNYMVAMVNKSLLPIRFQLPLVGDVIFLTQGLKYNLELLFFWGPGSLFQNKWNLQPK
                     YKRMGNRLELAQQLSRTICLLGLANLLLCPFILVWQILYAFFSYTEVIKREPGSLGAR
```

```
                     RWSLYGRLYLRHFNELNHELHTRLSRGYKPATKYMNSFTSPLLTVIAKNVGFFSGSIL
                     AVLISLTVYDEDVLTVQHILTAITLLGILVTVARSFIPDEHMVWCPEQLLQCVLAHIH
                     YIPDHWQGHAHKSETRDEMAQLFQYKAVFILEELLSPIITPFILIFSLRHKSLEIVDF
                     FRNFSVEVVGVGDICSFAQMDIRKHGNPQWLSEGQTEASVYQQAENGKTELSLMHFAI
                     TNPHWQPPLESSAFIGHLKEKVHQDAALAPPAQRILSEAPICSSLLSESGTAPDNLLA
                     SVLIHPVLTASGLPTRDRRFVQPTTTASAAASVLASLSLSQPPGRTRGHSGLFGACAE
                     SPMYRSDQTIFEESLTQADSQHQSLSRSSLMVSEFASAEMSLQAIYMHEVHQQQQHGP
                     GQPLGLWQAASVQRGRSSDSAVGTELRSVSSDHLSAYRDLRLGGWEEEDGQHQLGEEA
                     HGEQELLRGQASNSTS"
     CDS             join(148502..148610,168715..168758,169973..170037,
                     174977..175144,217744..217885,236402..237150,
                     279236..279389,291273..291452,310218..310460,
                     334041..334273,343357..343462,375712..375799,
                     397624..397787)
                     /gene="LOC138567588"
                     /note="Derived by automated computational analysis using
                     gene prediction method: Gnomon."
                     /codon_start=1
                     /product="autophagy-related protein 9A-like isoform X2"
                     /protein_id="XP_069502067.1"
                     /db_xref="GeneID:138567588"
                     /translation="MSGREEDREYQRLEDFEEDSPPGEEDLLVHVPEGLKDSWHHIKN
                     LDNFFTKIYNFHQKNGFACMVLSDFFEMVQFVFVVTFTTFLFCCVEYDILFANKPVNH
                     SHSATSPESYKVTLTDAILPSPQCAKRIQDDSWIIFLLAMAAVFWLYRLVKMFCNLLS
                     YWEIRSFYIRALKIPSDELCNFTWQEVQDRLVSLQREQQMCVHKKELTELDIYHRILR
                     FKNYMVAMVNKSLLPIRFQLPLVGDVIFLTQGLKYNLELLFFWGPGSLFQNKWNLQPK
                     YKRMGNRLELAQQLSRTICLLGLANLLLCPFILVWQILYAFFSYTEVIKREPGSLGAR
                     RWSLYGRLYLRHFNELNHELHTRLSRGYKPATKYMNSFTSPLLTVIAKNVGFFSGSIL
                     AVLISLTVYDEDVLTVQHILTAITLLGILVTVARSFIPDEHMVWCPEQLLQCVLAHIH
                     YIPDHWQGHAHKSETRDEMAQLFQYKAVFILEELLSPIITPFILIFSLRHKSLEIVDF
                     FRNFSVEVVGVGDICSFAQMDIRKHGNPQWLSEGQTEASVYQQAENGKTELSLMHFAI
                     TNPHWQPPLESSAFIGHLKEKVHQDAALAPPAQRILSEAPICSSLLSESGTAPDNLLA
                     SVLIHPVLTASGLPTRDRRFVQPTTTASAAASVLASLSLSQPPGRTRGHSGLFGACAE
                     SPMYRSDQTIFEESLTQADSQHQSLSRSSLMVSEFASAEMSLQAIYMHEVHQQQQHGP
                     GQPLGLWQAASVQRGRSSDSAVGTELRSVSSDHLSAYRDLRLGGWEEEDGQHQLGEEA
                     HGEQELLRGQASNSTS"
     CDS             join(148502..148610,168715..168760,169975..170037,
                     174977..175144,217744..217885,236402..237150,
                     279236..279389,291273..291452,310218..310460,
                     334041..334273,343357..343462,375712..375799,
                     397624..397787)
                     /gene="LOC138567588"
                     /note="Derived by automated computational analysis using
                     gene prediction method: Gnomon."
                     /codon_start=1
                     /product="autophagy-related protein 9A-like isoform X1"
                     /protein_id="XP_069502064.1"
                     /db_xref="GeneID:138567588"
                     /translation="MSGREEDREYQRLEDFEEDSPPGEEDLLVHVPEGLKDSWHHIKN
                     LDNFFTKIYNFHQKNGFACMVLSDFFEMVQFVFVVTFTTFLFCCVEYDILFANKPVNH
                     SHSATSPESYKVTLTDAILPSPQCAKRIQDDSWIIFLLAMAAVFWLYRLVKMFCNLLS
                     YWEIRSFYIRALKIPSDELCNFTWQEVQDRLVSLQREQQMCVHKKELTELDIYHRILR
                     FKNYMVAMVNKSLLPIRFQLPLVGDVIFLTQGLKYNLELLFFWGPGSLFQNKWNLQPK
                     YKRMGNRLELAQQLSRTICLLGLANLLLCPFILVWQILYAFFSYTEVIKREPGSLGAR
                     RWSLYGRLYLRHFNELNHELHTRLSRGYKPATKYMNSFTSPLLTVIAKNVGFFSGSIL
                     AVLISLTVYDEDVLTVQHILTAITLLGILVTVARSFIPDEHMVWCPEQLLQCVLAHIH
                     YIPDHWQGHAHKSETRDEMAQLFQYKAVFILEELLSPIITPFILIFSLRHKSLEIVDF
                     FRNFSVEVVGVGDICSFAQMDIRKHGNPQWLSEGQTEASVYQQAENGKTELSLMHFAI
                     TNPHWQPPLESSAFIGHLKEKVHQDAALAPPAQRILSEAPICSSLLSESGTAPDNLLA
                     SVLIHPVLTASGLPTRDRRFVQPTTTASAAASVLASLSLSQPPGRTRGHSGLFGACAE
                     SPMYRSDQTIFEESLTQADSQHQSLSRSSLMVSEFASAEMSLQAIYMHEVHQQQQHGP
                     GQPLGLWQAASVQRGRSSDSAVGTELRSVSSDHLSAYRDLRLGGWEEEDGQHQLGEEA
                     HGEQELLRGQASNSTS"
     CDS             join(148502..148610,168715..168760,169975..170037,
                     174977..175144,217744..217885,236402..237150,
                     279236..279389,291273..291452,310218..310460,
                     334041..334273,343357..343462,375712..375799,
                     397624..397787)
                     /gene="LOC138567588"
                     /note="Derived by automated computational analysis using
                     gene prediction method: Gnomon."
                     /codon_start=1
                     /product="autophagy-related protein 9A-like isoform X1"
                     /protein_id="XP_069502065.1"
                     /db_xref="GeneID:138567588"
                     /translation="MSGREEDREYQRLEDFEEDSPPGEEDLLVHVPEGLKDSWHHIKN
                     LDNFFTKIYNFHQKNGFACMVLSDFFEMVQFVFVVTFTTFLFCCVEYDILFANKPVNH
                     SHSATSPESYKVTLTDAILPSPQCAKRIQDDSWIIFLLAMAAVFWLYRLVKMFCNLLS
```

```
                     YWEIRSFYIRALKIPSDELCNFTWQEVQDRLVSLQREQQMCVHKKELTELDIYHRILR
                     FKNYMVAMVNKSLLPIRFQLPLVGDVIFLTQGLKYNLELLFFWGPGSLFQNKWNLQPK
                     YKRMGNRLELAQQLSRTICLLGLANLLLCPFILVWQILYAFFSYTEVIKREPGSLGAR
                     RWSLYGRLYLRHFNELNHELHTRLSRGYKPATKYMNSFTSPLLTVIAKNVGFFSGSIL
                     AVLISLTVYDEDVLTVQHILTAITLLGILVTVARSFIPDEHMVWCPEQLLQCVLAHIH
                     YIPDHWQGHAHKSETRDEMAQLFQYKAVFILEELLSPIITPFILIFSLRHKSLEIVDF
                     FRNFSVEVVGVGDICSFAQMDIRKHGNPQWLSEGQTEASVYQQAENGKTELSLMHFAI
                     TNPHWQPPLESSAFIGHLKEKVHQDAALAPPAQRILSEAPICSSLLSESGTAPDNLLA
                     SVLIHPVLTASGLPTRDRRFVQPTTTASAAASVLASLSLSQPPGRTRGHSGLFGACAE
                     SPMYRSDQTIFEESLTQADSQHQSLSRSSLMVSEFASAEMSLQAIYMHEVHQQQQHGP
                     GQPLGLWQAASVQRGRSSDSAVGTELRSVSSDHLSAYRDLRLGGWEEEDGQHQLGEEA
                     HGEQELLRGQASNSTS"
     CDS             join(170009..170037,174977..175144,217744..217885,
                     236402..237150,279236..279389,291273..291452,
                     310218..310460,334041..334273,343357..343462,
                     375712..375799,397624..397787)
                     /gene="LOC138567588"
                     /note="Derived by automated computational analysis using
                     gene prediction method: Gnomon."
                     /codon_start=1
                     /product="autophagy-related protein 9A-like isoform X3"
                     /protein_id="XP_069502068.1"
                     /db_xref="GeneID:138567588"
                     /translation="MVLSDFFEMVQFVFVVTFTTFLFCCVEYDILFANKPVNHSHSAT
                     SPESYKVTLTDAILPSPQCAKRIQDDSWIIFLLAMAAVFWLYRLVKMFCNLLSYWEIR
                     SFYIRALKIPSDELCNFTWQEVQDRLVSLQREQQMCVHKKELTELDIYHRILRFKNYM
                     VAMVNKSLLPIRFQLPLVGDVIFLTQGLKYNLELLFFWGPGSLFQNKWNLQPKYKRMG
                     NRLELAQQLSRTICLLGLANLLLCPFILVWQILYAFFSYTEVIKREPGSLGARRWSLY
                     GRLYLRHFNELNHELHTRLSRGYKPATKYMNSFTSPLLTVIAKNVGFFSGSILAVLIS
                     LTVYDEDVLTVQHILTAITLLGILVTVARSFIPDEHMVWCPEQLLQCVLAHIHYIPDH
                     WQGHAHKSETRDEMAQLFQYKAVFILEELLSPIITPFILIFSLRHKSLEIVDFFRNFS
                     VEVVGVGDICSFAQMDIRKHGNPQWLSEGQTEASVYQQAENGKTELSLMHFAITNPHW
                     QPPLESSAFIGHLKEKVHQDAALAPPAQRILSEAPICSSLLSESGTAPDNLLASVLIH
                     PVLTASGLPTRDRRFVQPTTTASAAASVLASLSLSQPPGRTRGHSGLFGACAESPMYR
                     SDQTIFEESLTQADSQHQSLSRSSLMVSEFASAEMSLQAIYMHEVHQQQQHGPGQPLG
                     LWQAASVQRGRSSDSAVGTELRSVSSDHLSAYRDLRLGGWEEEDGQHQLGEEAHGEQE
                     LLRGQASNSTS"
     mRNA            join(183059..183208,217744..217885,236402..237150,
                     279236..279389,291273..291452,310218..310460,
                     334041..334273,343357..343462,375712..375799,
                     397624..397808,444914..446942)
                     /gene="LOC138567588"
                     /product="autophagy-related protein 9A-like, transcript
                     variant X6"
                     /experiment="COORDINATES: polyA evidence [ECO:0006239]"
                     /transcript_id="XM_069645968.1"
                     /db_xref="GeneID:138567588"
     CDS             join(183183..183208,217744..217885,236402..237150,
                     279236..279389,291273..291452,310218..310460,
                     334041..334273,343357..343462,375712..375799,
                     397624..397787)
                     /gene="LOC138567588"
                     /note="Derived by automated computational analysis using
                     gene prediction method: Gnomon."
                     /codon_start=1
                     /product="autophagy-related protein 9A-like isoform X4"
                     /protein_id="XP_069502069.1"
                     /db_xref="GeneID:138567588"
                     /translation="MWGCCHLHRIQDDSWIIFLLAMAAVFWLYRLVKMFCNLLSYWEI
                     RSFYIRALKIPSDELCNFTWQEVQDRLVSLQREQQMCVHKKELTELDIYHRILRFKNY
                     MVAMVNKSLLPIRFQLPLVGDVIFLTQGLKYNLELLFFWGPGSLFQNKWNLQPKYKRM
                     GNRLELAQQLSRTICLLGLANLLLCPFILVWQILYAFFSYTEVIKREPGSLGARRWSL
                     YGRLYLRHFNELNHELHTRLSRGYKPATKYMNSFTSPLLTVIAKNVGFFSGSILAVLI
                     SLTVYDEDVLTVQHILTAITLLGILVTVARSFIPDEHMVWCPEQLLQCVLAHIHYIPD
                     HWQGHAHKSETRDEMAQLFQYKAVFILEELLSPIITPFILIFSLRHKSLEIVDFFRNF
                     SVEVVGVGDICSFAQMDIRKHGNPQWLSEGQTEASVYQQAENGKTELSLMHFAITNPH
                     WQPPLESSAFIGHLKEKVHQDAALAPPAQRILSEAPICSSLLSESGTAPDNLLASVLI
                     HPVLTASGLPTRDRRFVQPTTTASAAASVLASLSLSQPPGRTRGHSGLFGACAESPMY
                     RSDQTIFEESLTQADSQHQSLSRSSLMVSEFASAEMSLQAIYMHEVHQQQQHGPGQPL
                     GLWQAASVQRGRSSDSAVGTELRSVSSDHLSAYRDLRLGGWEEEDGQHQLGEEAHGEQ
                     ELLRGQASNSTS"
```

(C) Caecilian

```
LOCUS       NC_084489               25906 bp    DNA     linear   CON 18-DEC-2023
DEFINITION  Rhineura floridana isolate rRhiFlo1 chromosome 10, rRhiFlo1.hap2,
            whole genome shotgun sequence.
ACCESSION   NC_084489 REGION: 90849802..90875707
```

```
VERSION     NC_084489.1
DBLINK      BioProject: PRJNA1052099
            BioSample: SAMN34105704
            Assembly: GCF_030035675.1
KEYWORDS    WGS; RefSeq.
SOURCE      Rhineura floridana
  ORGANISM  Rhineura floridana
            Eukaryota; Metazoa; Chordata; Craniata; Vertebrata; Euteleostomi;
            Lepidosauria; Squamata; Bifurcata; Unidentata; Episquamata;
            Laterata; Lacertibaenia; Amphisbaenia; Rhineuridae; Rhineura.
COMMENT     REFSEQ INFORMATION: The reference sequence is identical to
            CM057614.1.
            Assembly name: rRhiFlo1.hap2
            The genomic sequence for this RefSeq record is from the
            whole-genome assembly released by the Vertebrate Genomes Project on
            2023/05/22. The original whole-genome shotgun project has the
            accession JASFZN000000000.1.

            ##Genome-Assembly-Data-START##
            Assembly Provider      :: Vertebrate Genomes Project
            Assembly Date          :: 06-APR-2023
            Assembly Method        :: Hifiasm + Hi-C phasing v. 0.18.5 +
                                      galaxy1; Bionano Solve v. 3.7; yahs v.
                                      1.2a.2 + galaxy0
            Assembly Name          :: rRhiFlo1.hap2
            Diploid                :: Haplotype 2
            Genome Representation  :: Full
            Expected Final Version :: No
            Genome Coverage        :: 38.35x
            Sequencing Technology  :: PacBio Sequel II HiFi; Bionano DLS; Arima
                                      Hi-C v2
            ##Genome-Assembly-Data-END##

            ##Genome-Annotation-Data-START##
            Annotation Provider         :: NCBI RefSeq
            Annotation Status           :: Full annotation
            Annotation Name             :: GCF_030035675.1-RS_2023_12
            Annotation Pipeline         :: NCBI eukaryotic genome annotation
                                           pipeline
            Annotation Software Version :: 10.2
            Annotation Method           :: Gnomon; cmsearch; tRNAscan-SE
            Features Annotated          :: Gene; mRNA; CDS; ncRNA
            Annotation Date             :: 12/15/2023
            ##Genome-Annotation-Data-END##
FEATURES             Location/Qualifiers
     source          1..25906
                     /organism="Rhineura floridana"
                     /mol_type="genomic DNA"
                     /isolate="rRhiFlo1"
                     /db_xref="taxon:261503"
                     /chromosome="10"
                     /sex="female"
                     /tissue_type="blood"
                     /dev_stage="adult"
                     /geo_loc_name="USA: Gainsville, Florida"
                     /lat_lon="29.6595 N 82.3174 W"
                     /collection_date="2020-02-14"
                     /collected_by="David Blackburn"
     gene            complement(<1..956)
                     /gene="ABCB8"
                     /note="ATP binding cassette subfamily B member 8; Derived
                     by automated computational analysis using gene prediction
                     method: Gnomon."
                     /db_xref="GeneID:133364858"
     mRNA            complement(<1..956)
                     /gene="ABCB8"
                     /product="ATP binding cassette subfamily B member 8,
                     transcript variant X2"
                     /experiment="COORDINATES: polyA evidence [ECO:0006239]"
                     /transcript_id="XM_061585747.1"
                     /db_xref="GeneID:133364858"
     mRNA            complement(<1..956)
                     /gene="ABCB8"
                     /product="ATP binding cassette subfamily B member 8,
                     transcript variant X4"
                     /experiment="COORDINATES: polyA evidence [ECO:0006239]"
                     /transcript_id="XM_061585749.1"
                     /db_xref="GeneID:133364858"
```

```
mRNA            complement(<1..82)
                /gene="ABCB8"
                /product="ATP binding cassette subfamily B member 8,
                transcript variant X1"
                /experiment="COORDINATES: polyA evidence [ECO:0006239]"
                /transcript_id="XM_061585746.1"
                /db_xref="GeneID:133364858"
gene            1..25906
                /gene="ATG9B"
                /note="autophagy related 9B; Derived by automated
                computational analysis using gene prediction method:
                Gnomon."
                /db_xref="GeneID:133364857"
mRNA            join(1..1272,4592..4740,4844..4887,6029..6093,6669..6842,
                8546..8687,9289..10037,12238..12391,12544..12723,
                13430..13678,13806..14023,21399..21501,21759..21825,
                24740..24881,25412..25906)
                /gene="ATG9B"
                /product="autophagy related 9B, transcript variant X2"
                /experiment="COORDINATES: polyA evidence [ECO:0006239]"
                /transcript_id="XM_061585745.1"
                /db_xref="GeneID:133364857"
mRNA            join(2..1272,2509..2581,4592..4740,4844..4887,6029..6093,
                6669..6842,8546..8687,9289..10037,12238..12391,
                12544..12723,13430..13678,13806..14023,21399..21501,
                21759..21825,24740..24881,25412..25906)
                /gene="ATG9B"
                /product="autophagy related 9B, transcript variant X1"
                /experiment="COORDINATES: polyA evidence [ECO:0006239]"
                /transcript_id="XM_061585744.1"
                /db_xref="GeneID:133364857"
CDS             join(4626..4740,4844..4887,6029..6093,6669..6842,
                8546..8687,9289..10037,12238..12391,12544..12723,
                13430..13678,13806..14023,21399..21501,21759..21825,
                24740..24852)
                /gene="ATG9B"
                /note="Derived by automated computational analysis using
                gene prediction method: Gnomon."
                /codon_start=1
                /product="autophagy-related protein 9B"
                /protein_id="XP_061441728.1"
                /db_xref="GeneID:133364857"
                /translation="MAASRQHHGDYHCLPDYEDDSPPEDEKQLLVHVTEGLKDSWHHI
                KNLDNFFTKIYHFHQKNGFASMMLSDVFELVQFLFVVTFTTFLLCCVEYDVLFANRPV
                NHTHTGGSLAPDRNKVTLPDAVLPTPQCAQRIRSSGWIIFLLVMAATFWLYRLVKVLC
                SLLGYWEIRAFYTKALKIPSDELCNRSWQEVQARLISLQREQQMCVHKRELSELDIHH
                RILRFKNYLVAMVNKSLLPVRFHLPLLGEAVFLTQGLKYNLELLFFWGPGSLFQGKWN
                LQPQYKRAGARLELARRLGRSMVLLGLANLLLCPFILVWQVLYAFFSYTEVLKRQPGS
                LGARRWSLYGRFYLRHFNELDHELQARLSRGYKPASKYMNSFTSPFLTVLAKNVGFFA
                GSILAVLIALTVYDEDVLTVQHILTAITLLGLIVTVARSFIPDEHLVWCPEQLLQCVL
                AHIHYIPDHWQGNAHKSETREELAQLFQYKAVFILEELLSPIVTPFLLIFALRAKALD
                IVDFFRNFSVEVVGVGDICSFAQLDIRNHGNPQWLSQGRTEASVYQQAENGKTELSLV
                HFAIANPRWQPPPESSLFIGHLKEKVQQDAAHAPPAQRLLAEAPLGASFLSDEGPCTV
                PDALLASVLTHPVLAAGTLMPQGERHLFTQAGSAASAAASILASLSCSQLLRRTGPLG
                ENSVYRSDNTLLGDSVAPPETHQAPLARSVVLSEFASAEMSLHAIYMHELHHQQQQHP
                ACARTPLQHSAQAASTRPSSRVCSQDLALGGWEEEEEDEPQAQQQEREKS"
CDS             join(4626..4740,4844..4887,6029..6093,6669..6842,
                8546..8687,9289..10037,12238..12391,12544..12723,
                13430..13678,13806..14023,21399..21501,21759..21825,
                24740..24852)
                /gene="ATG9B"
                /note="Derived by automated computational analysis using
                gene prediction method: Gnomon."
                /codon_start=1
                /product="autophagy-related protein 9B"
                /protein_id="XP_061441729.1"
                /db_xref="GeneID:133364857"
                /translation="MAASRQHHGDYHCLPDYEDDSPPEDEKQLLVHVTEGLKDSWHHI
                KNLDNFFTKIYHFHQKNGFASMMLSDVFELVQFLFVVTFTTFLLCCVEYDVLFANRPV
                NHTHTGGSLAPDRNKVTLPDAVLPTPQCAQRIRSSGWIIFLLVMAATFWLYRLVKVLC
                SLLGYWEIRAFYTKALKIPSDELCNRSWQEVQARLISLQREQQMCVHKRELSELDIHH
                RILRFKNYLVAMVNKSLLPVRFHLPLLGEAVFLTQGLKYNLELLFFWGPGSLFQGKWN
                LQPQYKRAGARLELARRLGRSMVLLGLANLLLCPFILVWQVLYAFFSYTEVLKRQPGS
                LGARRWSLYGRFYLRHFNELDHELQARLSRGYKPASKYMNSFTSPFLTVLAKNVGFFA
                GSILAVLIALTVYDEDVLTVQHILTAITLLGLIVTVARSFIPDEHLVWCPEQLLQCVL
                AHIHYIPDHWQGNAHKSETREELAQLFQYKAVFILEELLSPIVTPFLLIFALRAKALD
                IVDFFRNFSVEVVGVGDICSFAQLDIRNHGNPQWLSQGRTEASVYQQAENGKTELSLV
                HFAIANPRWQPPPESSLFIGHLKEKVQQDAAHAPPAQRLLAEAPLGASFLSDEGPCTV
```

```
                  PDALLASVLTHPVLAAGTLMPQGERHLFTQAGSAASAAASILASLSCSQLLRRTGPLG
                  ENSVYRSDNTLLGDSVAPPETHQAPLARSVVLSEFASAEMSLHAIYMHELHHQQQQHP
                  ACARTPLQHSAQAASTRPSSRVCSQDLALGGWEEEEEDEPQAQQQEREKS"
```

(D) Gecko

```
LOCUS       NC_059435              12845 bp    DNA     linear   CON 02-JUN-2022
DEFINITION  Sphaerodactylus townsendi isolate TG3544 linkage group LG11,
            MPM_Stown_v2.3, whole genome shotgun sequence.
ACCESSION   NC_059435 REGION: 78450056..78462900
VERSION     NC_059435.1
DBLINK      BioProject: PRJNA788548
            BioSample: SAMN20179316
            Assembly: GCF_021028975.2
KEYWORDS    WGS; RefSeq.
SOURCE      Sphaerodactylus townsendi
  ORGANISM  Sphaerodactylus townsendi
            Eukaryota; Metazoa; Chordata; Craniata; Vertebrata; Euteleostomi;
            Lepidosauria; Squamata; Bifurcata; Gekkota; Sphaerodactylidae;
            Sphaerodactylus.
REFERENCE   1  (bases 1 to 12845)
  AUTHORS   Pinto,B.J., Keating,S.E., Nielsen,S.V., Scantlebury,D.P., Daza,J.D.
            and Gamble,T.
  TITLE     Chromosome-level genome assembly reveals dynamic sex chromosomes in
            Neotropical leaf-litter geckos (Sphaerodactylidae: Sphaerodactylus)
  JOURNAL   J Hered (2022) In press
   PUBMED   35363859
  REMARK    Publication Status: Available-Online prior to print
COMMENT     REFSEQ INFORMATION: The reference sequence is identical to
            CM037624.2.
            Assembly name: MPM_Stown_v2.3
            The genomic sequence for this RefSeq record is from the
            whole-genome assembly released by the Marquette University on
            2022/04/08. The original whole-genome shotgun project has the
            accession JAHVXK000000000.2.

            ##Genome-Assembly-Data-START##
            Assembly Provider      :: Marquette University
            Assembly Method        :: SuperNova v. 2.1.1
            Assembly Name          :: MPM_Stown_v2.3
            Genome Representation  :: Full
            Expected Final Version :: No
            Genome Coverage        :: 160.0x
            Sequencing Technology  :: Illumina HiSeq
            ##Genome-Assembly-Data-END##

            ##Genome-Annotation-Data-START##
            Annotation Provider         :: NCBI
            Annotation Status           :: Full annotation
            Annotation Name             :: Sphaerodactylus townsendi Annotation
                                           Release 100
            Annotation Version          :: 100
            Annotation Pipeline         :: NCBI eukaryotic genome annotation
                                           pipeline
            Annotation Software Version :: 9.0
            Annotation Method           :: Best-placed RefSeq; Gnomon
            Features Annotated          :: Gene; mRNA; CDS; ncRNA
            ##Genome-Annotation-Data-END##
FEATURES             Location/Qualifiers
     source          1..12845
                     /organism="Sphaerodactylus townsendi"
                     /mol_type="genomic DNA"
                     /isolate="TG3544"
                     /db_xref="taxon:933632"
                     /sex="male"
                     /tissue_type="blood, liver"
                     /dev_stage="adult"
                     /collection_date="2012/2019"
                     /collected_by="Pinto, Daza, Gamble"
                     /linkage_group="LG11"
     gene            1..12845
                     /gene="ATG9B"
                     /note="Derived by automated computational analysis using
                     gene prediction method: Gnomon."
                     /db_xref="GeneID:125441335"
     mRNA            join(1..138,2793..2984,3078..3121,3546..3610,3928..4101,
                     4754..4895,5638..6386,7028..7181,7277..7456,7797..8045,
                     8127..8326,8778..8880,9025..9106,11428..11555,
```

```
                     12285..12845)
                     /gene="ATG9B"
                     /product="autophagy related 9B, transcript variant X1"
                     /experiment="COORDINATES: polyA evidence [ECO:0006239]"
                     /transcript_id="XM_048511861.1"
                     /db_xref="GeneID:125441335"
     CDS             join(2870..2984,3078..3121,3546..3610,3928..4101,
                     4754..4895,5638..6386,7028..7181,7277..7456,7797..8045,
                     8127..8326,8778..8880,9025..9106,11428..11516)
                     /gene="ATG9B"
                     /note="Derived by automated computational analysis using
                     gene prediction method: Gnomon."
                     /codon_start=1
                     /product="autophagy-related protein 9B isoform X1"
                     /protein_id="XP_048367818.1"
                     /db_xref="GeneID:125441335"
                     /translation="MAVSQEHYGDYHRLPDYAEDSPPEEEEQLLVHVTEGLKDSWHHI
                     KNLDNFFTKIYHFHQKNGFACMMLSDIFELVQFLFVVTFTTFLFCCVEYDVLFANRPV
                     NHTQPGGSLAPDRGKVTLPDAILPTPQCAERIRASGWIIFLLVMAATFWLYRLAKVLC
                     SLLGYWEIRAFYTKALKIPSDELCNRSWQEVQARLISLQREQQMCVHKRELTELDIHH
                     RILRFKNYLVAMVNKSLLPVRFRLPLLGEVVFLTQGLKYNLELLFFWGPGSLFQSKWN
                     LQPQYKRLGARLLLARRLGRSMVLLGLANLLLCPFILVWQVLYAFFSYTEVLKREPGS
                     LGARRWSLYGRLYLRHFNELDHELHARLSRGYKPASKYMNSFTSPLLTVLAKNVGFFA
                     GSILAVLIALTVYDEDVLTVQHILTTLTLLGLVVTVARSFIPDEHMVWCPEQLLQCVL
                     AHIHYIPDHWPGNAHKSETREEMAQLFQYKAVFILEELLSPIVTPFLLIFALRAKALD
                     IIDFFRNFSVEVVGVGDICSFAQLDIRNHGNPQWLSQGRTEASVYQQAENGKTELSLV
                     HFAITNPCWQPPPESSLFIGHLKEKVQQDAAHAPPAQRLLAEAPFSTSLLSDEGPSTM
                     PDALLASVLAHPILASQADRQRPGSTASAAASILASLSCSQLPRRSYPPPLGENSVYR
                     SDSTLLGDSVAPSASHQGPLTRSVVLSEFASAEMSLHAIYMHELHQQQQQPPPPAPS
                     PASFRTSEQAQADATTPDLSLGGWEEEEDPEAQQQQEREKC"
     mRNA            join(3551..3610,3932..4101,4754..4895,5638..6386,
                     7028..7181,7277..7456,7797..8045,8127..8326,8778..8880,
                     9025..9106,11428..11555,12285..12845)
                     /gene="ATG9B"
                     /product="autophagy related 9B, transcript variant X2"
                     /experiment="COORDINATES: polyA evidence [ECO:0006239]"
                     /transcript_id="XM_048511862.1"
                     /db_xref="GeneID:125441335"
     CDS             join(4791..4895,5638..6386,7028..7181,7277..7456,
                     7797..8045,8127..8326,8778..8880,9025..9106,11428..11516)
                     /gene="ATG9B"
                     /note="Derived by automated computational analysis using
                     gene prediction method: Gnomon."
                     /codon_start=1
                     /product="autophagy-related protein 9B isoform X2"
                     /protein_id="XP_048367819.1"
                     /db_xref="GeneID:125441335"
                     /translation="MAATFWLYRLAKVLCSLLGYWEIRAFYTKALKIPSDELCNRSWQ
                     EVQARLISLQREQQMCVHKRELTELDIHHRILRFKNYLVAMVNKSLLPVRFRLPLLGE
                     VVFLTQGLKYNLELLFFWGPGSLFQSKWNLQPQYKRLGARLLLARRLGRSMVLLGLAN
                     LLLCPFILVWQVLYAFFSYTEVLKREPGSLGARRWSLYGRLYLRHFNELDHELHARLS
                     RGYKPASKYMNSFTSPLLTVLAKNVGFFAGSILAVLIALTVYDEDVLTVQHILTTLTL
                     LGLVVTVARSFIPDEHMVWCPEQLLQCVLAHIHYIPDHWPGNAHKSETREEMAQLFQY
                     KAVFILEELLSPIVTPFLLIFALRAKALDIIDFFRNFSVEVVGVGDICSFAQLDIRNH
                     GNPQWLSQGRTEASVYQQAENGKTELSLVHFAITNPCWQPPPESSLFIGHLKEKVQQD
                     AAHAPPAQRLLAEAPFSTSLLSDEGPSTMPDALLASVLAHPILASQADRQRPGSTASA
                     AASILASLSCSQLPRRSYPPPLGENSVYRSDSTLLGDSVAPSASHQGPLTRSVVLSEF
                     ASAEMSLHAIYMHELHQQQQQPPPPAPSPASFRTSEQAQADATTPDLSLGGWEEEED
                     PEAQQQQEREKC"
```

(E) Green anole lizard

```
LOCUS       NC_085846              26236 bp    DNA     linear   CON 09-FEB-2024
DEFINITION  Anolis carolinensis isolate JA03-04 chromosome 6, rAnoCar3.1.pri,
            whole genome shotgun sequence.
ACCESSION   NC_085846 REGION: 119731820..119758055
VERSION     NC_085846.1
DBLINK      BioProject: PRJNA1071582
            BioSample: SAMN37993897
            Assembly: GCF_035594765.1
KEYWORDS    WGS; RefSeq.
SOURCE      Anolis carolinensis (green anole)
  ORGANISM  Anolis carolinensis
            Eukaryota; Metazoa; Chordata; Craniata; Vertebrata; Euteleostomi;
            Lepidosauria; Squamata; Bifurcata; Unidentata; Episquamata;
            Toxicofera; Iguania; Dactyloidae; Anolis.
COMMENT     REFSEQ INFORMATION: The reference sequence is identical to
            CM069372.1.
```

```
            Assembly name: rAnoCar3.1.pri
            The genomic sequence for this RefSeq record is from the
            whole-genome assembly released by the Louisiana State University on
            2024/01/12. The original whole-genome shotgun project has the
            accession JAWQEF000000000.1.

            ##Genome-Assembly-Data-START##
            Assembly Provider      :: Louisiana State University
            Assembly Date          :: 27-FEB-2023
            Assembly Method        :: HiFiasm v. 0.16.0-r369; bionano solve v.
                                      3.7_03302022_283; yahs v. 1.1
            Assembly Name          :: rAnoCar3.1.pri
            Genome Representation  :: Full
            Expected Final Version :: No
            Genome Coverage        :: 35.0x
            Sequencing Technology  :: PacBio Sequel
            ##Genome-Assembly-Data-END##

            ##Genome-Annotation-Data-START##
            Annotation Provider         :: NCBI RefSeq
            Annotation Status           :: Full annotation
            Annotation Name             :: GCF_035594765.1-RS_2024_02
            Annotation Pipeline         :: NCBI eukaryotic genome annotation
                                           pipeline
            Annotation Software Version :: 10.2
            Annotation Method           :: Best-placed RefSeq; Gnomon;
                                           cmsearch; tRNAscan-SE
            Features Annotated          :: Gene; mRNA; CDS; ncRNA
            Annotation Date             :: 02/01/2024
            ##Genome-Annotation-Data-END##
FEATURES             Location/Qualifiers
     source          1..26236
                     /organism="Anolis carolinensis"
                     /mol_type="genomic DNA"
                     /isolate="JA03-04"
                     /specimen_voucher="GMNH<USA-GA>:53075"
                     /db_xref="taxon:28377"
                     /chromosome="6"
                     /sex="male"
                     /tissue_type="liver, testis, kidney, lung"
                     /dev_stage="adult"
                     /geo_loc_name="USA: South Carolina,Aiken"
                     /lat_lon="33.217442 N 81.768044 W"
                     /collection_date="2021-03-12"
     gene            1..26236
                     /gene="atg9b"
                     /note="autophagy related 9B; Derived by automated
                     computational analysis using gene prediction method:
                     Gnomon."
                     /db_xref="GeneID:100557398"
     mRNA            join(1..739,6105..6252,6346..6389,6990..7054,13579..13734,
                     14582..14723,15924..16672,19213..19366,21845..22024,
                     24023..24271,24372..24592,24769..24871,24960..25035,
                     25309..25448,25732..25805,25862..26236)
                     /gene="atg9b"
                     /product="autophagy related 9B, transcript variant X2"
                     /experiment="COORDINATES: polyA evidence [ECO:0006239]"
                     /transcript_id="XM_062958164.1"
                     /db_xref="GeneID:100557398"
     mRNA            join(2842..2947,6105..6252,6346..6389,6990..7054,
                     13579..13734,14582..14723,15924..16672,19213..19366,
                     21845..22024,24023..24271,24372..24592,24769..24871,
                     24960..25035,25309..25448,25732..25805,25862..26236)
                     /gene="atg9b"
                     /product="autophagy related 9B, transcript variant X1"
                     /experiment="COORDINATES: polyA evidence [ECO:0006239]"
                     /transcript_id="XM_062958163.1"
                     /db_xref="GeneID:100557398"
     CDS             join(6138..6252,6346..6389,6990..7054,13579..13734,
                     14582..14723,15924..16672,19213..19366,21845..22024,
                     24023..24271,24372..24592,24769..24871,24960..25035,
                     25309..25409)
                     /gene="atg9b"
                     /note="Derived by automated computational analysis using
                     gene prediction method: Gnomon."
                     /codon_start=1
                     /product="autophagy-related protein 9B"
                     /protein_id="XP_062814233.1"
```

```
                     /db_xref="GeneID:100557398"
                     /translation="MAVSQEHYGDYHRLPDYEDDSPPEEEEELLIHVTEGLKDSWHHI
                     KNLDNFFTKIYHFHQKNGFACMMLSDVFELVQFLFVVAFTTFLLCCVEYDVLFANRPL
                     NHTQLDRNKVTLPDAILPTAQCTQRIRSSGWLIFLLAMAAVFWLYRLVKVLCSLLGYW
                     EIRAFYTKALKIPSAELCNCSWQEVQSRLISLQREQQMCVHKRELSELDIHHRILRFK
                     NYLVALVNKGLLPLRFRLPLLGRGVFLTQGLKYNLELLFFWGPGSLFQGKWNLQPQYK
                     RAGARLELARRLSRAMLLLGLANLLLCPFVLVWQGLYAFFSYTEVLKREPGSLGARRW
                     SLYGRLYLRHFNELDHELQARLGRGYKPASKYMNSFASPLLTALAKNVAFFAGSILAV
                     LIALTVYDEDVLTVQHILTAITLLGLVVTVARSFIPDEHLVWCPEQLLQCVLAHIHYI
                     PDHWQGNAHKSETREELSQLFQYKAVFILEELLSPIVTPFLLIFPLRARALDIIDFFR
                     NFSVEVVGVGDICSFAQLDIRNHGNPQWLSQGQTEASVQQQAENGKTELSLVHFAISN
                     PRWQPPPESSLFIGHLKEKVQQDAAQAPPAQRLLAEAPLGASLLSDEGLGAVPDALLA
                     SVLAHPVLSAASVASHSERRLFAHPGSTAGAAASVLASLSCSQLHRRSSAPPAENSVF
                     RSDSTVLGGRVAPPEARQGPLSRSVALSEFASAEMSLHAIYMHELRHQQHHRPREEGS
                     PHSPPQDSAEAGGGTRASQHMDRGLGGWPEEEAPRSQRPEREKS"
     CDS             join(6138..6252,6346..6389,6990..7054,13579..13734,
                     14582..14723,15924..16672,19213..19366,21845..22024,
                     24023..24271,24372..24592,24769..24871,24960..25035,
                     25309..25409)
                     /gene="atg9b"
                     /note="Derived by automated computational analysis using
                     gene prediction method: Gnomon."
                     /codon_start=1
                     /product="autophagy-related protein 9B"
                     /protein_id="XP_062814234.1"
                     /db_xref="GeneID:100557398"
                     /translation="MAVSQEHYGDYHRLPDYEDDSPPEEEEELLIHVTEGLKDSWHHI
                     KNLDNFFTKIYHFHQKNGFACMMLSDVFELVQFLFVVAFTTFLLCCVEYDVLFANRPL
                     NHTQLDRNKVTLPDAILPTAQCTQRIRSSGWLIFLLAMAAVFWLYRLVKVLCSLLGYW
                     EIRAFYTKALKIPSAELCNCSWQEVQSRLISLQREQQMCVHKRELSELDIHHRILRFK
                     NYLVALVNKGLLPLRFRLPLLGRGVFLTQGLKYNLELLFFWGPGSLFQGKWNLQPQYK
                     RAGARLELARRLSRAMLLLGLANLLLCPFVLVWQGLYAFFSYTEVLKREPGSLGARRW
                     SLYGRLYLRHFNELDHELQARLGRGYKPASKYMNSFASPLLTALAKNVAFFAGSILAV
                     LIALTVYDEDVLTVQHILTAITLLGLVVTVARSFIPDEHLVWCPEQLLQCVLAHIHYI
                     PDHWQGNAHKSETREELSQLFQYKAVFILEELLSPIVTPFLLIFPLRARALDIIDFFR
                     NFSVEVVGVGDICSFAQLDIRNHGNPQWLSQGQTEASVQQQAENGKTELSLVHFAISN
                     PRWQPPPESSLFIGHLKEKVQQDAAQAPPAQRLLAEAPLGASLLSDEGLGAVPDALLA
                     SVLAHPVLSAASVASHSERRLFAHPGSTAGAAASVLASLSCSQLHRRSSAPPAENSVF
                     RSDSTVLGGRVAPPEARQGPLSRSVALSEFASAEMSLHAIYMHELRHQQHHRPREEGS
                     PHSPPQDSAEAGGGTRASQHMDRGLGGWPEEEAPRSQRPEREKS"
```

(F) Asian vine snake

```
LOCUS       NC_080542              18292 bp    DNA     linear   CON 17-JUL-2023
DEFINITION  Ahaetulla prasina isolate Xishuangbanna chromosome 4, ASM2864084v1,
            whole genome shotgun sequence.
ACCESSION   NC_080542 REGION: 150282262..150300553
VERSION     NC_080542.1
DBLINK      BioProject: PRJNA993715
            BioSample: SAMN32882022
            Assembly: GCF_028640845.1
KEYWORDS    WGS; RefSeq.
SOURCE      Ahaetulla prasina
  ORGANISM  Ahaetulla prasina
            Eukaryota; Metazoa; Chordata; Craniata; Vertebrata; Euteleostomi;
            Lepidosauria; Squamata; Bifurcata; Unidentata; Episquamata;
            Toxicofera; Serpentes; Colubroidea; Colubridae; Ahaetuliinae;
            Ahaetulla.
REFERENCE   1  (bases 1 to 18292)
  AUTHORS   Tang,C.Y., Zhang,X., Xu,X., Sun,S., Peng,C., Song,M.H., Yan,C.,
            Sun,H., Liu,M., Xie,L., Luo,S.J. and Li,J.T.
  TITLE     Genetic mapping and molecular mechanism behind color variation in
            the Asian vine snake
  JOURNAL   Genome Biol 24 (1), 46 (2023)
   PUBMED   36895044
  REMARK    Publication Status: Online-Only
COMMENT     REFSEQ INFORMATION: The reference sequence is identical to
            CM054097.1.
            Assembly name: ASM2864084v1
            The genomic sequence for this RefSeq record is from the
            whole-genome assembly released by the Chinese Academy of Sciences
            on 2023/02/22. The original whole-genome shotgun project has the
            accession JAQQSC000000000.1.

            ##Genome-Assembly-Data-START##
            Assembly Provider      :: Chinese Academy of Sciences
            Assembly Method        :: NextDenovo v. 1.0; wtdbg2 v. 2.0;
                                      NextPolish v. 1.0; bowtie2 v. 2.3.2
```

```
            Genome Representation  :: Full
            Expected Final Version :: Yes
            Genome Coverage        :: 140.0x
            Sequencing Technology  :: MGISEQ-2000; Oxford Nanopore PromethION;
                                      Illumina HiSeq
            ##Genome-Assembly-Data-END##

            ##Genome-Annotation-Data-START##
            Annotation Provider         :: NCBI RefSeq
            Annotation Status           :: Full annotation
            Annotation Name             :: GCF_028640845.1-RS_2023_07
            Annotation Pipeline         :: NCBI eukaryotic genome annotation
                                           pipeline
            Annotation Software Version :: 10.1
            Annotation Method           :: Gnomon; cmsearch; tRNAscan-SE
            Features Annotated          :: Gene; mRNA; CDS; ncRNA
            Annotation Date             :: 07/13/2023
            ##Genome-Annotation-Data-END##
FEATURES             Location/Qualifiers
     source          1..18292
                     /organism="Ahaetulla prasina"
                     /mol_type="genomic DNA"
                     /isolate="Xishuangbanna"
                     /db_xref="taxon:499056"
                     /chromosome="4"
                     /sex="female"
                     /tissue_type="muscle"
                     /dev_stage="adult"
     gene            1..18292
                     /gene="ATG9B"
                     /note="autophagy related 9B; Derived by automated
                     computational analysis using gene prediction method:
                     Gnomon."
                     /db_xref="GeneID:131196511"
     mRNA            join(1..209,967..1183,1278..1321,2316..2380,3632..3814,
                     5535..5676,9892..10640,12356..12509,14317..14556,
                     14638..14831,15078..15180,15285..15339,15623..15769,
                     16251..18292)
                     /gene="ATG9B"
                     /product="autophagy related 9B, transcript variant X3"
                     /note="Derived by automated computational analysis using
                     gene prediction method: Gnomon. Supporting evidence
                     includes similarity to: 1 Protein"
                     /transcript_id="XM_058179323.1"
                     /db_xref="GeneID:131196511"
     mRNA            join(1..209,967..1183,1278..1321,2316..2380,3632..3814,
                     5535..5676,9892..10640,12356..12509,13748..13927,
                     14317..14556,14638..14831,15078..15180,15285..15339,
                     15623..15769,16251..18291)
                     /gene="ATG9B"
                     /product="autophagy related 9B, transcript variant X1"
                     /note="Derived by automated computational analysis using
                     gene prediction method: Gnomon. Supporting evidence
                     includes similarity to: 25 Proteins"
                     /transcript_id="XM_058179321.1"
                     /db_xref="GeneID:131196511"
     mRNA            join(236..388,967..1183,1278..1321,2316..2380,3632..3814,
                     5535..5676,9892..10640,12356..12509,13748..13927,
                     14317..14556,14638..14831,15078..15180,15285..15339,
                     15623..15769,16251..18291)
                     /gene="ATG9B"
                     /product="autophagy related 9B, transcript variant X2"
                     /note="Derived by automated computational analysis using
                     gene prediction method: Gnomon. Supporting evidence
                     includes similarity to: 25 Proteins"
                     /transcript_id="XM_058179322.1"
                     /db_xref="GeneID:131196511"
     CDS             join(1081..1183,1278..1321,2316..2380,3632..3814,
                     5535..5676,9892..10640,12356..12509,13748..13927,
                     14317..14556,14638..14831,15078..15180,15285..15339,
                     15623..15732)
                     /gene="ATG9B"
                     /note="Derived by automated computational analysis using
                     gene prediction method: Gnomon."
                     /codon_start=1
                     /product="autophagy-related protein 9B isoform X1"
                     /protein_id="XP_058035304.1"
                     /db_xref="GeneID:131196511"
```

```
                     /translation="MAGSQEHSGEYHPLPAYEDEDDEELLVHVTEGLKDSWHHIKNLD
                     NFFTKIYHFHQRNGFGCMMLSDVFELVQFLFVATFSTFLLCCVEYDILFANQPVNHTH
                     PDGGGRGFAPDRSKVTLPDAILPAAQCAERIQANSGIIFLLVMAATFWLYRLVKVLCS
                     LLSYWEIRSFYTKALKIPSEQLCNCSWQEVQARLISLQQEQQLCVHRRELTELDIHHR
                     ILRFKNYLVAMVNKSLLPVRFQLPLLGRGVFLTQGLKYNLELLLFWGPGSLFQGKWNL
                     QPQYKRAGARLELARRLERSLLLLGIANLLLCPFILVWQVLYAFFSYTEALKREPGSL
                     GARRWSLYGRLYLRHFNELDHELHARLSRGYKPASKYMNSFANPLLAVVARNIGFFAG
                     SLLAVLITLTVYDEDVLTVQHILTAITLLGLVVTLARAFIPDEHLVWCPEQLLQCVLA
                     HIHYIPDHWQGNAHKSETREELAQLFQYKAVFILEELLSPIVTPFLLIFALRAKALEI
                     VDFFRNFSVEVVGVGDICSFAQLDIRNHGNPQWLSQGRTEASVYQQAENGKTELSLVH
                     FAIANPHWQPPPESSLFIGHLKEKVQQDASHAQRLLAEGPLAASLLSDEGPGPVPDAL
                     LASVLTHPVLTERRLVAPPGSTASVTASILASLSSSQPSRHRSRPGEASVCNSQSPLL
                     ENSTAPSAPRQAPLSRSVLLSELASAEMSLHALYMHELHQQQAAARLALQPSAQTASS
                     RRLFDSRSQDRSLEVWEEESDEMQQQQQEPEKS"
     CDS             join(1081..1183,1278..1321,2316..2380,3632..3814,
                     5535..5676,9892..10640,12356..12509,13748..13927,
                     14317..14556,14638..14831,15078..15180,15285..15339,
                     15623..15732)
                     /gene="ATG9B"
                     /note="Derived by automated computational analysis using
                     gene prediction method: Gnomon."
                     /codon_start=1
                     /product="autophagy-related protein 9B isoform X1"
                     /protein_id="XP_058035305.1"
                     /db_xref="GeneID:131196511"
                     /translation="MAGSQEHSGEYHPLPAYEDEDDEELLVHVTEGLKDSWHHIKNLD
                     NFFTKIYHFHQRNGFGCMMLSDVFELVQFLFVATFSTFLLCCVEYDILFANQPVNHTH
                     PDGGGRGFAPDRSKVTLPDAILPAAQCAERIQANSGIIFLLVMAATFWLYRLVKVLCS
                     LLSYWEIRSFYTKALKIPSEQLCNCSWQEVQARLISLQQEQQLCVHRRELTELDIHHR
                     ILRFKNYLVAMVNKSLLPVRFQLPLLGRGVFLTQGLKYNLELLLFWGPGSLFQGKWNL
                     QPQYKRAGARLELARRLERSLLLLGIANLLLCPFILVWQVLYAFFSYTEALKREPGSL
                     GARRWSLYGRLYLRHFNELDHELHARLSRGYKPASKYMNSFANPLLAVVARNIGFFAG
                     SLLAVLITLTVYDEDVLTVQHILTAITLLGLVVTLARAFIPDEHLVWCPEQLLQCVLA
                     HIHYIPDHWQGNAHKSETREELAQLFQYKAVFILEELLSPIVTPFLLIFALRAKALEI
                     VDFFRNFSVEVVGVGDICSFAQLDIRNHGNPQWLSQGRTEASVYQQAENGKTELSLVH
                     FAIANPHWQPPPESSLFIGHLKEKVQQDASHAQRLLAEGPLAASLLSDEGPGPVPDAL
                     LASVLTHPVLTERRLVAPPGSTASVTASILASLSSSQPSRHRSRPGEASVCNSQSPLL
                     ENSTAPSAPRQAPLSRSVLLSELASAEMSLHALYMHELHQQQAAARLALQPSAQTASS
                     RRLFDSRSQDRSLEVWEEESDEMQQQQQEPEKS"
     CDS             join(1081..1183,1278..1321,2316..2380,3632..3814,
                     5535..5676,9892..10640,12356..12509,14317..14556,
                     14638..14831,15078..15180,15285..15339,15623..15732)
                     /gene="ATG9B"
                     /note="Derived by automated computational analysis using
                     gene prediction method: Gnomon."
                     /codon_start=1
                     /product="autophagy-related protein 9B isoform X2"
                     /protein_id="XP_058035306.1"
                     /db_xref="GeneID:131196511"
                     /translation="MAGSQEHSGEYHPLPAYEDEDDEELLVHVTEGLKDSWHHIKNLD
                     NFFTKIYHFHQRNGFGCMMLSDVFELVQFLFVATFSTFLLCCVEYDILFANQPVNHTH
                     PDGGGRGFAPDRSKVTLPDAILPAAQCAERIQANSGIIFLLVMAATFWLYRLVKVLCS
                     LLSYWEIRSFYTKALKIPSEQLCNCSWQEVQARLISLQQEQQLCVHRRELTELDIHHR
                     ILRFKNYLVAMVNKSLLPVRFQLPLLGRGVFLTQGLKYNLELLLFWGPGSLFQGKWNL
                     QPQYKRAGARLELARRLERSLLLLGIANLLLCPFILVWQVLYAFFSYTEALKREPGSL
                     GARRWSLYGRLYLRHFNELDHELHARLSRGYKPASKYMNSFANPLLAVVARNIGFFAG
                     SLLAVLITLTVYDEDVLTVQHILTAITLLGLVVTLARAFIPDEHLVWCPEQLLQCVLA
                     HIHYIPDHWQGNAHKSETREELAQLFQYKAWLSQGRTEASVYQQAENGKTELSLVHFA
                     IANPHWQPPPESSLFIGHLKEKVQQDASHAQRLLAEGPLAASLLSDEGPGPVPDALLA
                     SVLTHPVLTERRLVAPPGSTASVTASILASLSSSQPSRHRSRPGEASVCNSQSPLLEN
                     STAPSAPRQAPLSRSVLLSELASAEMSLHALYMHELHQQQAAARLALQPSAQTASSRR
                     LFDSRSQDRSLEVWEEESDEMQQQQQEPEKS"
```

(G) Chicken

```
LOCUS       NC_052533               8085 bp    DNA     linear   CON 01-MAR-2022
DEFINITION  Gallus gallus isolate bGalGal1 chromosome 2,
            bGalGal1.mat.broiler.GRCg7b, whole genome shotgun sequence.
ACCESSION   NC_052533 REGION: 333168..341252
VERSION     NC_052533.1
DBLINK      BioProject: PRJNA698609
            BioSample: SAMN15960293
            Assembly: GCF_016699485.2
KEYWORDS    WGS; RefSeq.
SOURCE      Gallus gallus (chicken)
  ORGANISM  Gallus gallus
            Eukaryota; Metazoa; Chordata; Craniata; Vertebrata; Euteleostomi;
            Archelosauria; Archosauria; Dinosauria; Saurischia; Theropoda;
```

```
            Coelurosauria; Aves; Neognathae; Galloanserae; Galliformes;
            Phasianidae; Phasianinae; Gallus.
COMMENT     REFSEQ INFORMATION: The reference sequence is identical to
            CM028483.1.
            Assembly name: bGalGal1.mat.broiler.GRCg7b
            The genomic sequence for this RefSeq record is from the
            whole-genome assembly released by the Vertebrate Genomes Project on
            2021/01/19. The original whole-genome shotgun project has the
            accession JAENSK000000000.1.

            ##Genome-Assembly-Data-START##
            Assembly Provider      :: Vertebrate Genomes Project
            Assembly Date          :: 22-JUL-2020
            Assembly Method        :: TrioCanu v. 1.8; purge_dups v. 1.0.0;
                                      Scaff 10x v. 4.1.0; Bionano solve v.
                                      3.2.1_04122018; Salsa2 HiC v. 2.2; Arrow
                                      polishing and gap filling v.
                                      SMRTLink7.0.1; Freebayes v. 1.3.1; gEVAL
                                      manual curation v. 2020-07-22; VGP trio
                                      assembly pipeline v. 1.6
            Assembly Name          :: bGalGal1.mat.broiler.GRCg7b
            Diploid                :: Principal pseudohaplotype
            Genome Representation  :: Full
            Expected Final Version :: No
            Genome Coverage        :: 102.01x
            Sequencing Technology  :: PacBio Sequel I CLR; Illumina NovaSeq;
                                      Arima Genomics Hi-C; Bionano Genomics DLS
            ##Genome-Assembly-Data-END##

            ##Genome-Annotation-Data-START##
            Annotation Provider         :: NCBI
            Annotation Status           :: Full annotation
            Annotation Name             :: Gallus gallus Annotation Release 106
            Annotation Version          :: 106
            Annotation Pipeline         :: NCBI eukaryotic genome annotation
                                           pipeline
            Annotation Software Version :: 9.0
            Annotation Method           :: Best-placed RefSeq; Gnomon
            Features Annotated          :: Gene; mRNA; CDS; ncRNA
            ##Genome-Annotation-Data-END##
FEATURES             Location/Qualifiers
     source          1..8085
                     /organism="Gallus gallus"
                     /mol_type="genomic DNA"
                     /isolate="bGalGal1"
                     /db_xref="taxon:9031"
                     /chromosome="2"
                     /sex="female"
                     /tissue_type="blood"
                     /geo_loc_name="USA: Fayetteville"
                     /lat_lon="36.0822 N 94.1719 W"
                     /collection_date="20-May-2019"
                     /collected_by="Nick Anthony"
     gene            complement(<1..119)
                     /gene="ABCB8"
                     /note="Derived by automated computational analysis using
                     gene prediction method: Gnomon."
                     /db_xref="CGNC:70051"
                     /db_xref="GeneID:107055879"
     mRNA            complement(<1..119)
                     /gene="ABCB8"
                     /product="ATP binding cassette subfamily B member 8,
                     transcript variant X1"
                     /experiment="COORDINATES: polyA evidence [ECO:0006239]"
                     /transcript_id="XM_046933446.1"
                     /db_xref="GeneID:107055879"
                     /db_xref="CGNC:70051"
     CDS             complement(<1..119)
                     /gene="ABCB8"
                     /note="Derived by automated computational analysis using
                     gene prediction method: Gnomon."
                     /codon_start=1
                     /product="mitochondrial potassium channel ATP-binding
                     subunit isoform X1"
                     /protein_id="XP_046789402.1"
                     /db_xref="GeneID:107055879"
                     /db_xref="CGNC:70051"
                     /translation="MAALPHRSGLRDGGGCGGAPRSGPRGAVRGLGRRWRRRAVQLRP
```

```
                     PHRPFRGAAMLLLCAGRAGCGLRAVREVLRSCRNGGSALRSAPRPAPRPAPLLVPAGL
                     ALGCVGAALGAAVRCQESDVRAAPTAAAPAAVPAAPRPEAEFQWAAFWALLRPQLLAL
                     SAAVVLALGAALLNVRIPVLLGQLVDVVAREARTHLEGYLRAARPPALRLLGLYSLQA
                     LLTFGYIALLSRVGEQVAASMRKALFVSLLRQDVAFFDAHRTGQLVARLTADVQEFKS
                     SFKLVISQGLRSSTQAAGCVLSLYLLSPRLTALLLLVLPALVSAGAALGSVLRALSRR
                     AQEQVAKATGVADEVLGNVRTVRAFAMEEQQAGLYCAEAERSSGMSQRLGLGIAAFQG
                     LSNLALNGIVLGTIFVGGSLMAGDQLSPGDLMSFLVASQTVQRSLANISILFGQVVRG
                     LSAGARVFEFMTLEPQVPLRGGDTIPSHSLLGHVAFRHVSFSYPTRPGHPVLQDFSLT
                     LPPGETVAIVGPSGGGKSTVAALLERFYEPTHGSITLDGRDIASLDPSWLRGSVIGFI
                     SQEPVLFGTTIMENIRFGKPGASDEEVFAAARLADADAFIRAFPDGYGTVVGERGAAL
                     SGGQRQRVALARALLKAPAVLVLDEATSALDAEAERAVQAALERAARGRTVLLIAHRL
                     STVRGANRIVVLAGGRVAEVGTHEELLRRGGLYAQLMRQQAEERRGAE"
     gene            1..8085
                     /gene="LOC107055991"
                     /note="Derived by automated computational analysis using
                     gene prediction method: Gnomon."
                     /db_xref="CGNC:78945"
                     /db_xref="GeneID:107055991"
     mRNA            join(1..183,1917..2018,2096..2139,2243..2307,2384..2545,
                     2628..2769,3453..4201,4669..4822,4891..5070,5135..5362,
                     6553..6749,7207..7309,7519..7597,7673..8085)
                     /gene="LOC107055991"
                     /product="autophagy-related protein 9A-like, transcript
                     variant X1"
                     /experiment="COORDINATES: cap analysis [ECO:0007248] and
                     polyA evidence [ECO:0006239]"
                     /transcript_id="XM_025147384.3"
                     /db_xref="GeneID:107055991"
                     /db_xref="CGNC:78945"
     mRNA            join(899..956,1917..2018,2096..2139,2243..2307,2384..2545,
                     2628..2769,3453..4201,4669..4822,4891..5070,5135..5362,
                     6553..6749,7207..7309,7519..7597,7673..8085)
                     /gene="LOC107055991"
                     /product="autophagy-related protein 9A-like, transcript
                     variant X2"
                     /experiment="COORDINATES: cap analysis [ECO:0007248] and
                     polyA evidence [ECO:0006239]"
                     /transcript_id="XM_025147383.3"
                     /db_xref="GeneID:107055991"
                     /db_xref="CGNC:78945"
     misc_RNA        join(1892..2018,2096..2139,2243..2307,2384..2545,
                     2628..2769,3453..4201,4669..4822,4891..5070,5135..5362,
                     7207..7309,7519..7597)
                     /gene="LOC107055991"
                     /product="autophagy-related protein 9A-like, transcript
                     variant X3"
                     /note="Derived by automated computational analysis using
                     gene prediction method: Gnomon. Supporting evidence
                     includes similarity to: 1 mRNA, 2 ESTs, 15 Proteins, and
                     100% coverage of the annotated genomic feature by RNAseq
                     alignments, including 55 samples with support for all
                     annotated introns"
                     /transcript_id="XR_005857934.2"
                     /db_xref="GeneID:107055991"
                     /db_xref="CGNC:78945"
     CDS             join(1922..2018,2096..2139,2243..2307,2384..2545,
                     2628..2769,3453..4201,4669..4822,4891..5070,5135..5362,
                     6553..6749,7207..7309,7519..7597,7673..7782)
                     /gene="LOC107055991"
                     /note="Derived by automated computational analysis using
                     gene prediction method: Gnomon."
                     /codon_start=1
                     /product="autophagy-related protein 9B"
                     /protein_id="XP_025003151.1"
                     /db_xref="GeneID:107055991"
                     /db_xref="CGNC:78945"
                     /translation="MAEVEYHRVEDPEEDSPPGEEELLLHVTEGRQGSWHHIKNLDDF
                     FTKIYHFHQKNGFACMLLSDLFELGQFVFVVAFSAFLLCCVRYDVLFADRPLNRSHVP
                     AERSKVTLPDAVLPAPQCARRIAASGWLVFLLAMAALFWLCRLLKVLRGLLSYWDIRR
                     FYGEALRIPAGELCSYSWQEVQARLMALQREQPLCVHKRELTELDIHHRILRFTNYAV
                     AMVNKSLLPVRFRLPLLGPVVFLTRGLHYNLELLLFRGPGALFLNSWSLRPQCKRAGE
                     RLALARHLARGMVLLGAANLLLCPLVLVWQLLYAFFSYAEVLRRRPGVLGTRRWSLYG
                     RLYLRHFNELQHELTARLGRGHRPATRYMNSFASPMLAVLARHVAFFAGSVLAVLIAL
                     TVYDEDVLTVQHILTAITLLGLLLTVARSFIPDEHSVQCPEQLLQRVLAHTHYMPQHW
                     QGSAGAPETRNEMAQLFQYKAVFILEELLSPVLTPLILIFALPSRALDIVDFFRNFTV
                     EVVGVGDVCSFAQLDVRRHGNPQWLSEGQTEASVYQRAENGKTELSLMHFAISNPRWQ
                     PPPHSGLFLNHVKERLQRDAAPHGPAEGALRASLLGDGSAAPDALLSSVLTHPLLAAS
                     GLLPWDRRFSQPCSTASAAAGVLLSLSAPLPGRGRGPLDSPERPLPEESPVLSESRLL
```

```
                     SLSRSAVLAEVASAEMSLHAIYIHELHLQQQQSPVGPWVAAGAPKRPSVTTGSAARAS
                     QHELREMPLGGWAEEDEEDEDEEEEQSAM"
     CDS             join(1922..2018,2096..2139,2243..2307,2384..2545,
                     2628..2769,3453..4201,4669..4822,4891..5070,5135..5362,
                     6553..6749,7207..7309,7519..7597,7673..7782)
                     /gene="LOC107055991"
                     /note="Derived by automated computational analysis using
                     gene prediction method: Gnomon."
                     /codon_start=1
                     /product="autophagy-related protein 9B"
                     /protein_id="XP_025003152.1"
                     /db_xref="GeneID:107055991"
                     /db_xref="CGNC:78945"
                     /translation="MAEVEYHRVEDPEEDSPPGEEELLLHVTEGRQGSWHHIKNLDDF
                     FTKIYHFHQKNGFACMLLSDLFELGQFVFVVAFSAFLLCCVRYDVLFADRPLNRSHVP
                     AERSKVTLPDAVLPAPQCARRIAASGWLVFLLAMAALFWLCRLLKVLRGLLSYWDIRR
                     FYGEALRIPAGELCSYSWQEVQARLMALQREQPLCVHKRELTELDIHHRILRFTNYAV
                     AMVNKSLLPVRFRLPLLGPVVFLTRGLHYNLELLLFRGPGALFLNSWSLRPQCKRAGE
                     RLALARHLARGMVLLGAANLLLCPLVLVWQLLYAFFSYAEVLRRRPGVLGTRRWSLYG
                     RLYLRHFNELQHELTARLGRGHRPATRYMNSFASPMLAVLARHVAFFAGSVLAVLIAL
                     TVYDEDVLTVQHILTAITLLGLLLTVARSFIPDEHSVQCPEQLLQRVLAHTHYMPQHW
                     QGSAGAPETRNEMAQLFQYKAVFILEELLSPVLTPLILIFALPSRALDIVDFFRNFTV
                     EVVGVGDVCSFAQLDVRRHGNPQWLSEGQTEASVYQRAENGKTELSLMHFAISNPRWQ
                     PPPHSGLFLNHVKERLQRDAAPHGPAEGALRASLLGDGSAAPDALLSSVLTHPLLAAS
                     GLLPWDRRFSQPCSTASAAAGVLLSLSAPLPGRGRGPLDSPERPLPEESPVLSESRLL
                     SLSRSAVLAEVASAEMSLHAIYIHELHLQQQQSPVGPWVAAGAPKRPSVTTGSAARAS
                     QHELREMPLGGWAEEDEEDEDEEEEQSAM"
```

(H) Ostrich

```
LOCUS       NC_090943               7467 bp    DNA     linear   CON 04-OCT-2024
DEFINITION  Struthio camelus isolate bStrCam1 chromosome 2, bStrCam1.hap1,
            whole genome shotgun sequence.
ACCESSION   NC_090943 REGION: 528202..535668
VERSION     NC_090943.1
DBLINK      BioProject: PRJNA1165802
            BioSample: SAMN42123890
            Assembly: GCF_040807025.1
KEYWORDS    WGS; RefSeq.
SOURCE      Struthio camelus (African ostrich)
  ORGANISM  Struthio camelus
            Eukaryota; Metazoa; Chordata; Craniata; Vertebrata; Euteleostomi;
            Archelosauria; Archosauria; Dinosauria; Saurischia; Theropoda;
            Coelurosauria; Aves; Palaeognathae; Struthioniformes;
            Struthionidae; Struthio.
COMMENT     REFSEQ INFORMATION: The reference sequence is identical to
            CM081587.1.
            Assembly name: bStrCam1.hap1
            The genomic sequence for this RefSeq record is from the
            whole-genome assembly released by the Vertebrate Genomes Project on
            2024/07/22. The original whole-genome shotgun project has the
            accession JBFMIB000000000.1.

            ##Genome-Assembly-Data-START##
            Assembly Provider      :: Vertebrate Genomes Project
            Assembly Date          :: 15-JUN-2024
            Assembly Method        :: Hifiasm + Hi-C phasing v. 0.19.8 +
                                      galaxy0; yahs v. 1.2a.2 + galaxy1
            Assembly Name          :: bStrCam1.hap1
            Diploid                :: Haplotype 1
            Genome Representation  :: Full
            Expected Final Version :: No
            Genome Coverage        :: 80.9x
            Sequencing Technology  :: PacBio Revio HiFi; Arima Hi-C v2
            ##Genome-Assembly-Data-END##

            ##Genome-Annotation-Data-START##
            Annotation Provider         :: NCBI RefSeq
            Annotation Status           :: Full annotation
            Annotation Name             :: GCF_040807025.1-RS_2024_10
            Annotation Pipeline         :: NCBI eukaryotic genome annotation
                                           pipeline
            Annotation Software Version :: 10.3
            Annotation Method           :: Gnomon; cmsearch; tRNAscan-SE
            Features Annotated          :: Gene; mRNA; CDS; ncRNA
            Annotation Date             :: 10/02/2024
            ##Genome-Annotation-Data-END##
FEATURES             Location/Qualifiers
```

```
source          1..7467
                /organism="Struthio camelus"
                /mol_type="genomic DNA"
                /isolate="bStrCam1"
                /db_xref="taxon:8801"
                /chromosome="2"
                /sex="female"
                /tissue_type="embryonic fibroblast cells"
                /dev_stage="embryo"
                /geo_loc_name="USA: New York, NY"
                /lat_lon="40.750519 N 73.967474 W"
                /collection_date="2022-09-29"
                /collected_by="Anna Keyte, Matthew Biegler"
gene            1..7467
                /gene="ATG9B"
                /note="autophagy related 9B; Derived by automated
                computational analysis using gene prediction method:
                Gnomon."
                /db_xref="GeneID:138066234"
mRNA            join(1..495,2138..2255,3514..3557,3701..3765,3866..4036,
                4127..4268,4375..5123,5236..5389,5471..5896,5970..6181,
                6346..6448,6895..6985,7084..7202,7336..7467)
                /gene="ATG9B"
                /product="autophagy related 9B, transcript variant X2"
                /note="Derived by automated computational analysis using
                gene prediction method: Gnomon. Supporting evidence
                includes similarity to: 17 Proteins"
                /transcript_id="XM_068933882.1"
                /db_xref="GeneID:138066234"
mRNA            join(1786..1880,2138..2255,3514..3557,3701..3765,
                3866..4036,4127..4268,4375..5123,5236..5389,5471..5896,
                5970..6181,6346..6448,6895..6985,7084..7202,7336..7467)
                /gene="ATG9B"
                /product="autophagy related 9B, transcript variant X1"
                /note="Derived by automated computational analysis using
                gene prediction method: Gnomon. Supporting evidence
                includes similarity to: 17 Proteins"
                /transcript_id="XM_068933881.1"
                /db_xref="GeneID:138066234"
CDS             join(2159..2255,3514..3557,3701..3765,3866..4036,
                4127..4268,4375..5123,5236..5389,5471..5896,5970..6181,
                6346..6448,6895..6985,7084..7187)
                /gene="ATG9B"
                /note="Derived by automated computational analysis using
                gene prediction method: Gnomon."
                /codon_start=1
                /product="autophagy-related protein 9B"
                /protein_id="XP_068789982.1"
                /db_xref="GeneID:138066234"
                /translation="MAAQGEYRRLEDEEDSPPGEEELLVHVSEGLRDSWHHVKNLDNF
                FTKIYHFHQKNGFACMMLSDLFELVQFLFVVTFTTFLLCCVEYDVLFANRPLNHSHAG
                GLAPDRSKVTLPDAILPAPRCAQRIRANGWIIFLLVMAAVFWLYRLVKVLCSLLSYWE
                IRTFYIKALNIPSDGLCNYSWQEVQARLISLQREQQMCVHKKELTELDIYHRILRFKN
                YTVAMVNKSLLPVRFRLPLLGHVVFLTQGLKYNLELLFFWGPGSLFQNKWNLQPQYKR
                AGSRLELAQRLARTMVLLGLANLLLCPFVLVWQVLYAFFSYTEIIKREPGSLGARRWS
                LYGRHYLRHFNELNHELQARLSRGYKPATKYMNSFTSPLLTVLAKNVGFFAGSILAVL
                IVLTVYDEDVLTVQHILTAITLLGLVVTLARSFIPDEHMAWCPEQLLQRVLAHIHYMP
                DHWQGNASKSETRNEMAQLFQYKAVFILEELLSPILTPLILIFALPARALDIVDFFRN
                FTVEVVGVGDICSFAQLDVRNHGNPQWLSAGQTEASLYQQAENGKTELSLMHFAITNP
                RWQPPPQSELFLSHLKEKVQQDAAAAPPAQRILAEGPLGASLLSDESAAAPDGLLASL
                LARPLLSASGLVARDRRFVQPCSAASAAASLLASLASPPPGRTRVPATDSPGGRSDRR
                LPEESLLLSESRLLSLSRSALLSEVASAEMSLHAIYMHELHQQQQQGPGPHTVGVCMA
                TGAPKLPTVTAGSAARASQAQHRELPLGGWAEEDEEDEEEEEKTG"
CDS             join(2159..2255,3514..3557,3701..3765,3866..4036,
                4127..4268,4375..5123,5236..5389,5471..5896,5970..6181,
                6346..6448,6895..6985,7084..7187)
                /gene="ATG9B"
                /note="Derived by automated computational analysis using
                gene prediction method: Gnomon."
                /codon_start=1
                /product="autophagy-related protein 9B"
                /protein_id="XP_068789983.1"
                /db_xref="GeneID:138066234"
                /translation="MAAQGEYRRLEDEEDSPPGEEELLVHVSEGLRDSWHHVKNLDNF
                FTKIYHFHQKNGFACMMLSDLFELVQFLFVVTFTTFLLCCVEYDVLFANRPLNHSHAG
                GLAPDRSKVTLPDAILPAPRCAQRIRANGWIIFLLVMAAVFWLYRLVKVLCSLLSYWE
                IRTFYIKALNIPSDGLCNYSWQEVQARLISLQREQQMCVHKKELTELDIYHRILRFKN
                YTVAMVNKSLLPVRFRLPLLGHVVFLTQGLKYNLELLFFWGPGSLFQNKWNLQPQYKR
```

```
                    AGSRLELAQRLARTMVLLGLANLLLCPFVLVWQVLYAFFSYTEIIKREPGSLGARRWS
                    LYGRHYLRHFNELNHELQARLSRGYKPATKYMNSFTSPLLTVLAKNVGFFAGSILAVL
                    IVLTVYDEDVLTVQHILTAITLLGLVVTLARSFIPDEHMAWCPEQLLQRVLAHIHYMP
                    DHWQGNASKSETRNEMAQLFQYKAVFILEELLSPILTPLILIFALPARALDIVDFFRN
                    FTVEVVGVGDICSFAQLDVRNHGNPQWLSAGQTEASLYQQAENGKTELSLMHFAITNP
                    RWQPPPQSELFLSHLKEKVQQDAAAAPPAQRILAEGPLGASLLSDESAAAPDGLLASL
                    LARPLLSASGLVARDRRFVQPCSAASAAASLLASLASPPPGRTRVPATDSPGGRSDRR
                    LPEESLLLSESRLLSLSRSALLSEVASAEMSLHAIYMHELHQQQQQGPGPHTVGVCMA
                    TGAPKLPTVTAGSAARASQAQHRELPLGGWAEEDEEDEEEEEKTG"
```

(I) Echidna

```
LOCUS       NC_052078              20791 bp    DNA     linear   CON 06-JAN-2021
DEFINITION  Tachyglossus aculeatus isolate mTacAcu1 chromosome 13,
            mTacAcu1.pri, whole genome shotgun sequence.
ACCESSION   NC_052078 REGION: complement(4516689..4537479)
VERSION     NC_052078.1
DBLINK      BioProject: PRJNA687514
            BioSample: SAMN12634963
            Assembly: GCF_015852505.1
KEYWORDS    WGS; RefSeq.
SOURCE      Tachyglossus aculeatus (Australian echidna)
  ORGANISM  Tachyglossus aculeatus
            Eukaryota; Metazoa; Chordata; Craniata; Vertebrata; Euteleostomi;
            Mammalia; Monotremata; Tachyglossidae; Tachyglossus.
COMMENT     REFSEQ INFORMATION: The reference sequence is identical to
            CM027612.1.
            Assembly name: mTacAcu1.pri
            The genomic sequence for this RefSeq record is from the
            whole-genome assembly released by the Vertebrate Genomes Project on
            2020/12/09. The original whole-genome shotgun project has the
            accession JADRJE000000000.1.

            ##Genome-Assembly-Data-START##
            Assembly Provider      :: Vertebrate Genomes Project
            Assembly Date          :: 26-OCT-2020
            Assembly Method        :: FALCON v. falcon-2018.31.08-03.06;
                                      FALCON-Unzip v. 6.0.0.47841; purge_dups
                                      v. github
                                      ca23030ccf4254dfd2d3a5ea90d0eed41c24f88b
                                      ca23030ccf4254dfd2d3a5ea90d0eed41c24f88b;
                                      Scaff10x v. 4.1.0; Bionano Solve v.
                                      3.2.1_04122018; Salsa2 HiC v. 2.2; Polish
                                      pbgcpp v. 1.9.0; Longranger Align v.
                                      2.2.2; freebayes v. 1.3.1; gEVAL manual
                                      curation v. 2020-09-17; Manual Curation
                                      v. 2020-10-26; VGP assembly pipeline v.
                                      1.6
            Assembly Name          :: mTacAcu1.pri
            Diploid                :: Principal pseudohaplotype
            Genome Representation  :: Full
            Expected Final Version :: No
            Genome Coverage        :: 58.29x
            Sequencing Technology  :: PacBio Sequel I CLR; Illumina NovaSeq;
                                      Bionano Genomics DLS; Phase Genomics
            ##Genome-Assembly-Data-END##

            ##Genome-Annotation-Data-START##
            Annotation Provider         :: NCBI
            Annotation Status           :: Full annotation
            Annotation Name             :: Tachyglossus aculeatus Annotation
                                           Release 100
            Annotation Version          :: 100
            Annotation Pipeline         :: NCBI eukaryotic genome annotation
                                           pipeline
            Annotation Software Version :: 8.5
            Annotation Method           :: Best-placed RefSeq; Gnomon
            Features Annotated          :: Gene; mRNA; CDS; ncRNA
            ##Genome-Annotation-Data-END##
FEATURES             Location/Qualifiers
     source          1..20791
                     /organism="Tachyglossus aculeatus"
                     /mol_type="genomic DNA"
                     /isolate="mTacAcu1"
                     /db_xref="taxon:9261"
                     /chromosome="13"
                     /sex="male"
                     /tissue_type="liver"
```

```
                     /dev_stage="adult"
                     /geo_loc_name="Australia: Upper Barnard River, New South
                     Wales"
                     /lat_lon="31.647305 S 151.500011 E"
                     /collected_by="Tasman Daish, Frank Grutzner"
     gene            complement(<1..34)
                     /gene="ABCB8"
                     /note="Derived by automated computational analysis using
                     gene prediction method: Gnomon."
                     /db_xref="GeneID:119936213"
     mRNA            complement(<1..31)
                     /gene="ABCB8"
                     /product="ATP binding cassette subfamily B member 8,
                     transcript variant X1"
                     /note="Derived by automated computational analysis using
                     gene prediction method: Gnomon. Supporting evidence
                     includes similarity to: 12 Proteins, and 100% coverage of
                     the annotated genomic feature by RNAseq alignments,
                     including 9 samples with support for all annotated
                     introns"
                     /transcript_id="XM_038755702.1"
                     /db_xref="GeneID:119936213"
     mRNA            complement(<1..34)
                     /gene="ABCB8"
                     /product="ATP binding cassette subfamily B member 8,
                     transcript variant X2"
                     /note="Derived by automated computational analysis using
                     gene prediction method: Gnomon. Supporting evidence
                     includes similarity to: 100% coverage of the annotated
                     genomic feature by RNAseq alignments, including 7 samples
                     with support for all annotated introns"
                     /transcript_id="XM_038755703.1"
                     /db_xref="GeneID:119936213"
     gene            1..20791
                     /gene="ATG9B"
                     /note="Derived by automated computational analysis using
                     gene prediction method: Gnomon."
                     /db_xref="GeneID:119936092"
     mRNA            join(1..40,3390..3515,3664..3737,4305..4467,4655..4698,
                     4923..4987,5637..5825,6910..7051,8323..9077,10593..10800,
                     18146..18325,18726..18974,19188..19333,19502..19598,
                     20180..20321,20416..20565,20700..20791)
                     /gene="ATG9B"
                     /product="autophagy related 9B"
                     /note="Derived by automated computational analysis using
                     gene prediction method: Gnomon. Supporting evidence
                     includes similarity to: 9 Proteins, and 42% coverage of
                     the annotated genomic feature by RNAseq alignments"
                     /transcript_id="XM_038755472.1"
                     /db_xref="GeneID:119936092"
     CDS             join(1..40,3390..3515,3664..3737,4305..4467,4655..4698,
                     4923..4987,5637..5825,6910..7051,8323..9077,10593..10800,
                     18146..18325,18726..18974,19188..19333,19502..19598,
                     20180..20321,20416..20552)
                     /gene="ATG9B"
                     /note="Derived by automated computational analysis using
                     gene prediction method: Gnomon."
                     /codon_start=1
                     /product="autophagy-related protein 9B"
                     /protein_id="XP_038611400.1"
                     /db_xref="GeneID:119936092"
                     /translation="MAAAVTLTRPWAPCSAIRNSAYPASPSARIPSPWLGLRSGLRKA
                     SLPPEWALPPKSSDGARLRSPEPSSPAQRTEPVSPMDVLRMGPLVPQQDYERLEDCDP
                     DRDPGYDPPDSQESPLPGEEEELLLHVPEGLRGSWHHIKNLDSFFTKIYNYHQRSGFV
                     CMLLEDAFQLGQFLFIVTFTTFLLRCVDYDVLFANRPSNRTWPGPPGSRRPPGPLHSK
                     VTLSDAILPAAQCSQRIRSCSPLLFLLVLATGYWLFRLLRSLCSLLGYWDIRAFYREA
                     LKILPGEVSTVPWTEVQSRLLDLQRGGGLCVLPRPLSELDVHHRILRFPNYLVALANK
                     GLLPARCPLPWGGSAAFLSRGLALNLDLLLFRGPASLFRGGWALPAAYKRGARRRALA
                     ARLRRTLLLLALANLLLCPLVLAWQGLRAFFSHAELLRREPGALGTRRWSRLARLQLR
                     HFNELPHSLRARLARAYRPAAGYLRAASPPGPLAALLARHAAFFAGALLAALLALTVY
                     DEDVLAVEHVLTAITGLGVAVTVARRFIPEEELCGRSPEPLLQAALAHMHYLPEGPEE
                     AEEQQQQQTGPGPLRTPATRAARIRRQMAQLLQYKAVSLLEELLSPIVTPLTLLFWFR
                     PRALEIIDFFHHFTVDVAGVGDVCSFALMDIQRHGHPEWLSAGQTEASVSQRAEGGKT
                     ELSLMRFALAHPRWRPPGHSSRFLGQLRDRVQQDAAAWGATSPRSPLTPGLGPSLPTD
                     SITSTPEALLTSLLVHPLMPPREAALAPPCPAAATASLLASLSSSTSRLAADESCVSL
                     GATGVRSPVLLSELASAEMSLHAIYLHQLHQQQQQQELLGETAPTLPALWPSDPSGPT
                     PPLSPSLEEQNQPPPGHGLSSDPGPGPERRWGPDGERALPLGGWQDDDAQQEPATAPS
                     SSSP"
```

(J) Opossum

```
LOCUS       NC_077231              11982 bp    DNA     linear   CON 05-JUN-2023
DEFINITION  Monodelphis domestica isolate mMonDom1 chromosome 5, mMonDom1.pri,
            whole genome shotgun sequence.
ACCESSION   NC_077231 REGION: complement(228007519..228019500)
VERSION     NC_077231.1
DBLINK      BioProject: PRJNA967365
            BioSample: SAMN31801966
            Assembly: GCF_027887165.1
KEYWORDS    WGS; RefSeq.
SOURCE      Monodelphis domestica (gray short-tailed opossum)
  ORGANISM  Monodelphis domestica
            Eukaryota; Metazoa; Chordata; Craniata; Vertebrata; Euteleostomi;
            Mammalia; Metatheria; Didelphimorphia; Didelphidae; Monodelphis.
COMMENT     REFSEQ INFORMATION: The reference sequence is identical to
            CM051227.1.
            Assembly name: mMonDom1.pri
            The genomic sequence for this RefSeq record is from the
            whole-genome assembly released by the Vertebrate Genomes Project on
            2023/01/18. The original whole-genome shotgun project has the
            accession JAQIHD000000000.1.

            ##Genome-Assembly-Data-START##
            Assembly Provider      :: Vertebrate Genomes Project
            Assembly Date          :: 05-MAY-2022
            Assembly Method        :: HiFiasm v. 0.15.4 + galaxy; purge_dups v.
                                      1.2.5 + galaxy3; Bionano Solve v. 3.6.1.
                                      + galaxy2; salsa v. 2.3 + galaxy0
            Assembly Name          :: mMonDom1.pri
            Diploid                :: Principal Haplotype
            Genome Representation  :: Full
            Expected Final Version :: No
            Genome Coverage        :: 32.9x
            Sequencing Technology  :: PacBio Sequel II HiFi; Bionano Genomics
                                      DLS; Arima Hi-C v2
            ##Genome-Assembly-Data-END##

            ##Genome-Annotation-Data-START##
            Annotation Provider         :: NCBI RefSeq
            Annotation Status           :: Full annotation
            Annotation Name             :: GCF_027887165.1-RS_2023_05
            Annotation Pipeline         :: NCBI eukaryotic genome annotation
                                           pipeline
            Annotation Software Version :: 10.1
            Annotation Method           :: Best-placed RefSeq; Gnomon;
                                           cmsearch; tRNAscan-SE
            Features Annotated          :: Gene; mRNA; CDS; ncRNA
            Annotation Date             :: 05/17/2023
            ##Genome-Annotation-Data-END##
FEATURES             Location/Qualifiers
     source          1..11982
                     /organism="Monodelphis domestica"
                     /mol_type="genomic DNA"
                     /isolate="mMonDom1"
                     /db_xref="taxon:13616"
                     /chromosome="5"
                     /sex="male"
                     /tissue_type="spleen, liver"
                     /dev_stage="adult"
                     /geo_loc_name="USA: Davis, California"
                     /lat_lon="38.544907 N 121.740517 W"
                     /collection_date="2020-11-01"
                     /collected_by="Leah Krubitzer, Rebecca Hodge"
     gene            1..11982
                     /gene="ATG9B"
                     /note="autophagy related 9B; Derived by automated
                     computational analysis using gene prediction method:
                     Gnomon."
                     /db_xref="GeneID:100018064"
     mRNA            join(1..1808,2166..2325,2692..2735,2986..3050,3336..3515,
                     3937..4078,6350..7104,7656..7809,8129..8308,9138..9386,
                     9582..9727,9991..10087,10331..10475,11002..11157,
                     11281..11978)
                     /gene="ATG9B"
                     /product="autophagy related 9B, transcript variant X1"
                     /note="Derived by automated computational analysis using
```

```
                     gene prediction method: Gnomon. Supporting evidence
                     includes similarity to: 2 Proteins"
                     /transcript_id="XM_007504619.2"
                     /db_xref="GeneID:100018064"
     mRNA            join(1716..2325,2692..2735,2986..3050,3336..3515,
                     3937..4078,6350..7104,7656..7809,8129..8308,9138..9386,
                     9582..9727,9991..10087,10331..10475,11002..11157,
                     11281..11982)
                     /gene="ATG9B"
                     /product="autophagy related 9B, transcript variant X2"
                     /note="Derived by automated computational analysis using
                     gene prediction method: Gnomon. Supporting evidence
                     includes similarity to: 3 Proteins"
                     /transcript_id="XM_007504618.2"
                     /db_xref="GeneID:100018064"
     CDS             join(1806..1808,2166..2325,2692..2735,2986..3050,
                     3336..3515,3937..4078,6350..7104,7656..7809,8129..8308,
                     9138..9386,9582..9727,9991..10087,10331..10475,
                     11002..11141)
                     /gene="ATG9B"
                     /note="Derived by automated computational analysis using
                     gene prediction method: Gnomon."
                     /codon_start=1
                     /product="autophagy-related protein 9B isoform X1"
                     /protein_id="XP_007504681.1"
                     /db_xref="GeneID:100018064"
                     /translation="MWSLQNSSGLRMGPLIPEQDYERLEDCDPDCDNTQNSPLPEDQE
                     PLLHVPEGLKGSWHHIQNLDSFFTKLYNYHQRNGFACMFLEDAFQLGQFVFVVTFTTF
                     LLRCVDYDILFANRPVNHTRPGSHQNPGILQSKVTLSDAILPAAQCAQRVRSSSILIF
                     LLFLASGFWLFRLLRSLCNLLNYWDIRTFYKEALHIPPEDLSKVPWGEVQSRLLALQR
                     GGGLCVLPRPLTELDVHHRILRYPNYLVALANKGLLPARFPLPWGGSATFLSQGLALN
                     LDLLLFRGPISLFRGGWALPSTYKRSCQRRTLAIRLRWTLLFLALVNLALCPLVLAWQ
                     GLHAFFSHAELLRREPGALGMRRWSRLARLQLRHFNELPHELRARLARAYRPAAHYLR
                     AAAPPAPLPALLARHAGFFAGALLAALLVLTVYDEDVLAVEHVLTAMTGLGVALTVAR
                     CFIPEEELLGRSPKPLLQAALAHMHYLPEEPGSVREANTHHQMSQLLQYKAISLLEEL
                     LSPVLTPLALLLWFRPRALEIIDFFRHFTVDVSGVGDVCSFALMDVRRHGHPQWLSAG
                     QTEASISQRAEGGKTELSLMRFSLAHPRWRPPGHSSRFLEQLRDRVQQDAALWAATPI
                     RSPPTPGLGPSFLSDSTTSAPEALLASLLLHPLLPPRELSPVASCPAAATASLLASLS
                     GSMSQAGHDQSCVSPGGPGVQNPALFSEIASAEMSLHAIYLHQLHQQQQQQQQQQQQQQE
                     PWGETSATPLPRLWPSPQRPLTPEDETLTWHSDGTSPASSSSSSPRQQWGPKGASNLP
                     LGGWQEEENVQMEPVSNPGSG"
     CDS             join(1806..2325,2692..2735,2986..3050,3336..3515,
                     3937..4078,6350..7104,7656..7809,8129..8308,9138..9386,
                     9582..9727,9991..10087,10331..10475,11002..11141)
                     /gene="ATG9B"
                     /note="Derived by automated computational analysis using
                     gene prediction method: Gnomon."
                     /codon_start=1
                     /product="autophagy-related protein 9B isoform X2"
                     /protein_id="XP_007504680.1"
                     /db_xref="GeneID:100018064"
                     /translation="MVRRMGWGRTKRHQERWGDLGATLLPPLLPASRCLGPGRGRISV
                     FSMSPTPYTRSSPSSFLASIPWPLSPARTSGVTQPLSALETSSPHGESSGTPSSAPSG
                     PRSYSTPCLASVTPLPSQWSLQNSSGLRMGPLIPEQDYERLEDCDPDCDNTQNSPLPE
                     DQEPLLHVPEGLKGSWHHIQNLDSFFTKLYNYHQRNGFACMFLEDAFQLGQFVFVVTF
                     TTFLLRCVDYDILFANRPVNHTRPGSHQNPGILQSKVTLSDAILPAAQCAQRVRSSSI
                     LIFLLFLASGFWLFRLLRSLCNLLNYWDIRTFYKEALHIPPEDLSKVPWGEVQSRLLA
                     LQRGGGLCVLPRPLTELDVHHRILRYPNYLVALANKGLLPARFPLPWGGSATFLSQGL
                     ALNLDLLLFRGPISLFRGGWALPSTYKRSCQRRTLAIRLRWTLLFLALVNLALCPLVL
                     AWQGLHAFFSHAELLRREPGALGMRRWSRLARLQLRHFNELPHELRARLARAYRPAAH
                     YLRAAAPPAPLPALLARHAGFFAGALLAALLVLTVYDEDVLAVEHVLTAMTGLGVALT
                     VARCFIPEEELLGRSPKPLLQAALAHMHYLPEEPGSVREANTHHQMSQLLQYKAISLL
                     EELLSPVLTPLALLLWFRPRALEIIDFFRHFTVDVSGVGDVCSFALMDVRRHGHPQWL
                     SAGQTEASISQRAEGGKTELSLMRFSLAHPRWRPPGHSSRFLEQLRDRVQQDAALWAA
                     TPIRSPPTPGLGPSFLSDSTTSAPEALLASLLLHPLLPPRELSPVASCPAAATASLLA
                     SLSGSMSQAGHDQSCVSPGGPGVQNPALFSEIASAEMSLHAIYLHQLHQQQQQQQQQQQQ
                     QQEPWGETSATPLPRLWPSPQRPLTPEDETLTWHSDGTSPASSSSSSPRQQWGPKGAS
                     NLPLGGWQEEENVQMEPVSNPGSG"
```

(K) Manatee

```
LOCUS       NW_004443956           10346 bp    DNA     linear   CON 29-JAN-2024
DEFINITION  Trichechus manatus latirostris isolate Lorelei unplaced genomic
            scaffold, TriManLat1.0 scaffold00020, whole genome shotgun
            sequence.
ACCESSION   NW_004443956 REGION: complement(12474436..12484781)
VERSION     NW_004443956.1
DBLINK      BioProject: PRJNA189960
```

```
            BioSample: SAMN00632092
            Assembly: GCF_000243295.1
KEYWORDS    WGS; RefSeq.
SOURCE      Trichechus manatus latirostris (Florida manatee)
  ORGANISM  Trichechus manatus latirostris
            Eukaryota; Metazoa; Chordata; Craniata; Vertebrata; Euteleostomi;
            Mammalia; Eutheria; Afrotheria; Sirenia; Trichechidae; Trichechus.
COMMENT     REFSEQ INFORMATION: The reference sequence is identical to
            JH594626.1.
            Assembly name: TriManLat1.0
            The genomic sequence for this RefSeq record is from the
            whole-genome assembly released by the Broad Institute on
            2012/01/23. The original whole-genome shotgun project has the
            accession AHIN00000000.1.

            ##Genome-Assembly-Data-START##
            Assembly Provider     :: Broad Institute
            Assembly Method       :: AllPaths v. R38542
            Assembly Name         :: TriManLat1.0
            Genome Coverage       :: 150x
            Sequencing Technology :: Illumina HiSeq
            ##Genome-Assembly-Data-END##

            ##Genome-Annotation-Data-START##
            Annotation Provider         :: NCBI RefSeq
            Annotation Status           :: Updated annotation
            Annotation Name             :: GCF_000243295.1-RS_2024_01
            Annotation Pipeline         :: NCBI eukaryotic genome annotation
                                           pipeline
            Annotation Software Version :: 10.2
            Annotation Method           :: Best-placed RefSeq; Gnomon;
                                           cmsearch; tRNAscan-SE
            Features Annotated          :: Gene; mRNA; CDS; ncRNA
            Annotation Date             :: 01/26/2024
            ##Genome-Annotation-Data-END##
FEATURES             Location/Qualifiers
     source          1..10346
                     /organism="Trichechus manatus latirostris"
                     /mol_type="genomic DNA"
                     /isolate="Lorelei"
                     /sub_species="latirostris"
                     /db_xref="taxon:127582"
                     /chromosome="Unknown"
                     /sex="female"
     gene            complement(<1..95)
                     /gene="ABCB8"
                     /note="ATP binding cassette subfamily B member 8; Derived
                     by automated computational analysis using gene prediction
                     method: Gnomon."
                     /db_xref="GeneID:101357200"
     mRNA            complement(<1..95)
                     /gene="ABCB8"
                     /product="ATP binding cassette subfamily B member 8,
                     transcript variant X1"
                     /note="Derived by automated computational analysis using
                     gene prediction method: Gnomon. Supporting evidence
                     includes similarity to: 8 mRNAs, 15 Proteins, and 80%
                     coverage of the annotated genomic feature by RNAseq
                     alignments"
                     /transcript_id="XM_004372735.3"
                     /db_xref="GeneID:101357200"
     mRNA            complement(<1..95)
                     /gene="ABCB8"
                     /product="ATP binding cassette subfamily B member 8,
                     transcript variant X2"
                     /note="Derived by automated computational analysis using
                     gene prediction method: Gnomon. Supporting evidence
                     includes similarity to: 2 mRNAs, 8 Proteins, and 81%
                     coverage of the annotated genomic feature by RNAseq
                     alignments"
                     /transcript_id="XM_023727942.1"
                     /db_xref="GeneID:101357200"
     mRNA            complement(<1..89)
                     /gene="ABCB8"
                     /product="ATP binding cassette subfamily B member 8,
                     transcript variant X3"
                     /note="Derived by automated computational analysis using
                     gene prediction method: Gnomon. Supporting evidence
```

```
                     includes similarity to: 1 mRNA, 11 Proteins, and 75%
                     coverage of the annotated genomic feature by RNAseq
                     alignments"
                     /transcript_id="XM_023727943.1"
                     /db_xref="GeneID:101357200"
     CDS             complement(<1..2)
                     /gene="ABCB8"
                     /note="Derived by automated computational analysis using
                     gene prediction method: Gnomon."
                     /codon_start=1
                     /product="mitochondrial potassium channel ATP-binding
                     subunit isoform X1"
                     /protein_id="XP_004372792.1"
                     /db_xref="GeneID:101357200"
                     /translation="MLVHLFRVGIQGGPVPGRPLLLRLQTFTAVRHSDSHHNSYLIRS
                     VAQLRSQLRPHLPRAPPAPSRSPLSAWHWVGGVLLGPVVLRKCSRLCLVARCEAEETE
                     EAPAISFRPYVEESRFNWKLFWQFLRPHLLVLAAAIVLALGAALVNVQIPLLLGQLVE
                     IVAKYTREHMGSFMTESRNLSTRLLLLYGIQGLLTFGYLVLLSRIGERMAVDMRRTLF
                     CSLLRQDIAFFDAKKTGQLVSRLTTDVQEFKSSFKLVISQGLRSCTQVAGCLVSLSML
                     STRLTLLLMVATPTLMGVGTLMGSALRKLSRQSQEQIARATGVADEALGNVRTVRAFA
                     MEHREEERYGAELKVSCCKAEELGRGIALFQGLSNIAFNCMVLGTLFIGGSLVAGQQL
                     TGGDLMSFLVASQTVQRSMANLSVLFGQVVRGLSAGARVFEYMTLSPCIPLSGGSFIP
                     KEDLHGAITFQNVGFSYPCRPGFEVLKDFTLTLPPGKTVALVGQSGGGKTTVASLLER
                     FYDPTAGVVLLDGRDLRTLDPSWLRGQVIGFISQEPVLFGTTIMENIRFGKLEASDEE
                     VYAAAREANAHDFITSFPDGYGTIVGERGATLSGGQKQRLAIARALIKQPTVLIMDEA
                     TSALDTESEGVVQEALDRASAGRTVLIIAHRLSTVRGAHHIVVMADGRVWEAGTHEEL
                     LKKRGLYAELIRRQALEAPLTSTPPLEKPKGPRNRHP"
     CDS             complement(<1..2)
                     /gene="ABCB8"
                     /note="Derived by automated computational analysis using
                     gene prediction method: Gnomon."
                     /codon_start=1
                     /product="mitochondrial potassium channel ATP-binding
                     subunit isoform X2"
                     /protein_id="XP_023583710.1"
                     /db_xref="GeneID:101357200"
                     /translation="MLVHLFRVGIQGGPVPGRPLLLRLQTFTAVRHSDSHHNSYLIRS
                     VAQLRSQLRPHLPRAPPAPSRSPLSAWHWVGGVLLGPVVLRKCSRLCLVARCEAEETE
                     EAPAISFRPYVEESRFNWKLFWQFLRPHLLVLAAAIVLALGAALVNVQIPLLLGQLVE
                     IVAKYTREHMGSFMTESRNLSTRLLLLYGIQGLLTFGYLVLLSRIGERMAVDMRRTLF
                     CSLLRQDIAFFDAKKTGQLVSRLTTDVQEFKSSFKLVISQGLRSCTQVAGCLVSLSML
                     STRLTLLLMVATPTLMGVGTLMGSALRKLSRQSQEQIARATGVADEALGNVRTVRAFA
                     MEHREEERYGAELKVSCCKAEELGRGIALFQGLSNIAFNCMVLGTLFIGGSLVAGQQL
                     TGGDLMSFLVASQTVQRSMANLSVLFGQVVRGLSAGARVFEYMTLSPCIPLSGGSFIP
                     KEDLHGAITFQNVGFSYPCRPGFEVLKDFTLTLPPGKTVALVGQSGGGKTTVASLLER
                     FYDPTAGVVLLDGRDLRTLDPSWLRGQVIGFISQEPVLFGTTIMENIRFGKLEASDEE
                     VYAAAREANAHDFITSFPDGYGTIVGERGATLSGGQKQRLAIARALIKQPTVLIMDEA
                     TSALDTESEGVVQEALDRASAGRTVLIIAGTHEELLKKRGLYAELIRRQALEAPLTST
                     PPLEKPKGPRNRHP"
     gene            1..10346
                     /gene="ATG9B"
                     /note="autophagy related 9B; Derived by automated
                     computational analysis using gene prediction method:
                     Gnomon."
                     /db_xref="GeneID:101346894"
     mRNA            join(1..73,3055..3585,3719..3762,3926..3990,4213..4371,
                     5144..5285,7062..7816,7919..8072,8260..8439,8983..9219,
                     9411..9544,9712..9808,10009..10132,10219..10346)
                     /gene="ATG9B"
                     /product="autophagy related 9B"
                     /note="Derived by automated computational analysis using
                     gene prediction method: Gnomon. Supporting evidence
                     includes similarity to: 11 Proteins, and 33% coverage of
                     the annotated genomic feature by RNAseq alignments"
                     /transcript_id="XM_012554477.1"
                     /db_xref="GeneID:101346894"
     CDS             join(1..73,3055..3585,3719..3762,3926..3990,4213..4371,
                     5144..5285,7062..7816,7919..8072,8260..8439,8983..9219,
                     9411..9544,9712..9808,10009..10132,10219..10346)
                     /gene="ATG9B"
                     /note="Derived by automated computational analysis using
                     gene prediction method: Gnomon."
                     /codon_start=1
                     /product="autophagy-related protein 9B"
                     /protein_id="XP_012409931.1"
                     /db_xref="GeneID:101346894"
                     /translation="MMGQEGRSSQQQQFHPRPTEGSMLAEWVVGGKRGRLGQWGDLGS
                     ASVPLLPISPPPPCPPSCWGPGGGRISIFSLSSAPHPRSSPSLFLPPALGSPCPMLQV
```

```
                     SGPSQPHHSALSTPARAQPVMTSTSASPSWGSHSIPPLASVTPPPSHRCPQDPPGLRI
                     GPLIPEQDYERLEDCDPEGSQDSPLHGEEQQPLLHVPEGLRCSWHHIQNLDSFFTKIY
                     SYHQRNGFACILLEDVFQLGQFIFIVTFTTFLICCVDYNILFTNQPNNHTSGPLHSKV
                     TLSDAILSSAQCAERIRSSPLLVFLLVQSVGFWLFQLLRSVCNLFSYWDIQVFYRQAL
                     HIPPEELSSMPWAEVQSRLLALQRSGGLCVQPRPLTELDVHHRILRYTNYQVALANKG
                     LLPARCALPCGGSVAFLSRGLALNVDLLLFRGPFSLFRGGWELPDAYKRSEQRGALAA
                     RLGRAALLLAAANLALSPLVLAWQVLHAFYSHAELLRREPGALGAQRWSRFARLQLRH
                     FNELPHELCARLDRAYRPATAFLRAAAQPAPMLALLARQLVFYAGTLFAALVVLTIYD
                     EDVLAVQHVLTAMTVLGVTATVARSFLPEEHCQGRSAPLLLQAALAHMHYLPEEPSPT
                     RNAGAYRQMAQLLQYRAVSLLEELLSPLLTPLFLYFWFRPRALEIIDFFHHFTVDVAG
                     VGDICSFALMDVKRHGHPQWLSAGQTEVSLAQRAEDGKTELSLMRFFLAHPQWRPPGH
                     SSKFLGHLRGRVQQDAATWGATSVRSPTTPGLLSDSTSSLPEVFLANLLVHPLLPPKD
                     LSPTSPCPAAATASLLASISRISQDPSCVSPGGPGGQKLTQLPELASAEMSLHAIYLH
                     QLHQQQHQELWGEASASSLSRPWSSPFHPLSPDEGKPSWSSDGSSPASSPRQQWRTQR
                     AQNLFPGGFQETTETQQQPGQAPSTD"
```

(L) Elephant

```
LOCUS       NC_087363             12488 bp    DNA     linear   CON 05-APR-2024
DEFINITION  Loxodonta africana isolate mLoxAfr1 chromosome 22, mLoxAfr1.hap2,
            whole genome shotgun sequence.
ACCESSION   NC_087363 REGION: complement(64928179..64940666)
VERSION     NC_087363.1
DBLINK      BioProject: PRJNA1092696
            BioSample: SAMN32756554
            Assembly: GCF_030014295.1
KEYWORDS    WGS; RefSeq.
SOURCE      Loxodonta africana (African savanna elephant)
  ORGANISM  Loxodonta africana
            Eukaryota; Metazoa; Chordata; Craniata; Vertebrata; Euteleostomi;
            Mammalia; Eutheria; Afrotheria; Proboscidea; Elephantidae;
            Loxodonta.
COMMENT     REFSEQ INFORMATION: The reference sequence is identical to
            CM057440.1.
            Assembly name: mLoxAfr1.hap2
            The genomic sequence for this RefSeq record is from the
            whole-genome assembly released by the Vertebrate Genomes Project on
            2023/05/16. The original whole-genome shotgun project has the
            accession JASCQR000000000.1.

            ##Genome-Assembly-Data-START##
            Assembly Provider      :: Vertebrate Genomes Project
            Assembly Date          :: 27-MAR-2023
            Assembly Method        :: Hifiasm + Hi-C phasing v. 0.16.1 +
                                      galaxy3; Bionano Solve v. 3.7; yahs v.
                                      1.2a.2 + galaxy0
            Assembly Name          :: mLoxAfr1.hap2
            Diploid                :: Haplotype 2
            Genome Representation  :: Full
            Expected Final Version :: No
            Genome Coverage        :: 43.0x
            Sequencing Technology  :: PacBio Sequel II HiFi; Bionano DLS; Arima
                                      Hi-C v2
            ##Genome-Assembly-Data-END##

            ##Genome-Annotation-Data-START##
            Annotation Provider         :: NCBI RefSeq
            Annotation Status           :: Full annotation
            Annotation Name             :: GCF_030014295.1-RS_2024_04
            Annotation Pipeline         :: NCBI eukaryotic genome annotation
                                           pipeline
            Annotation Software Version :: 10.2
            Annotation Method           :: Best-placed RefSeq; Gnomon;
                                           cmsearch; tRNAscan-SE
            Features Annotated          :: Gene; mRNA; CDS; ncRNA
            Annotation Date             :: 04/02/2024
            ##Genome-Annotation-Data-END##
FEATURES             Location/Qualifiers
     source          1..12488
                     /organism="Loxodonta africana"
                     /mol_type="genomic DNA"
                     /isolate="mLoxAfr1"
                     /db_xref="taxon:9785"
                     /chromosome="22"
                     /sex="male"
                     /cell_line="Cultured endothelial cells from umbilical
                     cord; passage 7"
                     /tissue_type="Original tissue source of cells is from
```

```
                     umbilical cord"
                     /dev_stage="juvenile"
                     /geo_loc_name="USA: Louisville Zoo, Kentucky"
                     /lat_lon="38.2057 N 85.7071 W"
                     /collection_date="2022-02-22"
                     /collected_by="Austin Bow"
     gene            complement(<1..119)
                     /gene="ABCB8"
                     /note="ATP binding cassette subfamily B member 8; Derived
                     by automated computational analysis using gene prediction
                     method: Gnomon."
                     /db_xref="GeneID:100669470"
     mRNA            complement(<1..119)
                     /gene="ABCB8"
                     /product="ATP binding cassette subfamily B member 8,
                     transcript variant X1"
                     /experiment="COORDINATES: polyA evidence [ECO:0006239]"
                     /transcript_id="XM_003410012.4"
                     /db_xref="GeneID:100669470"
     CDS             complement(<1..2)
                     /gene="ABCB8"
                     /note="Derived by automated computational analysis using
                     gene prediction method: Gnomon."
                     /codon_start=1
                     /product="mitochondrial potassium channel ATP-binding
                     subunit isoform X1"
                     /protein_id="XP_003410060.2"
                     /db_xref="GeneID:100669470"
                     /translation="MLVHLFRVGIRGGPAPGRPLLLRLQTFTAVRHSDGHHSSNLLRS
                     VARLRSQLQPRLPRAPPAPSRSPLSAWHWVGGILLGPVVLRKCSHLCLVARCEAEEAP
                     PVSSRPYVEESHFNWKLFWQFLRPHLLVLGVAIVLALGAALVNVQIPLLLGQLVEIVA
                     KYTREHAGSFMTESRSLGTRLLLLYGIQGLLTFGYLVLLSRIGERMAVDMRRTLFCSL
                     LRQDIAFFDAKKTGQLVSRLTTDVQEFKSSFKLVISQGLRSCTQVAGCLVSLSMLSTR
                     LTLLLMVATPTLMGVGTLMGSALRKLSRQSQEQIARATGVADEALGNVRTVRAFAMEQ
                     REEERYGAELEASCCKAEELGRGIALFQGLSNIAFNCMVLGTLFIGGSLVAGQELTGG
                     DLMSFLVASQTVQRSMANLSILFGQVVRGLSAGARVFEYMTLSPCIPLTGGCCIPKED
                     LHGTITFQNVGFSYPCRPGFEVLKDFTLTLPPGKTVALVGQSGGGKTTVASLLERFYD
                     PTAGVVLLDGRDLRTLDPSWLRGQVIGFISQEPVLFGTTIMENIRFGKLEASDEEVYA
                     AAREANAHDFITSFPDGYGTVVGERGAALSGGQKQRLAIARALIKQPTVLIMDEATSA
                     LDTESEQVVQEALDRASAGRTVLIIAHRLSTVRGAHRIIVMADGRVWEAGTHEELLKK
                     RGLYAELIRRQALDSPLTSTPPLEKPKGPRNRHPEA"
     mRNA            complement(<1..119)
                     /gene="ABCB8"
                     /product="ATP binding cassette subfamily B member 8,
                     transcript variant X2"
                     /note="Derived by automated computational analysis using
                     gene prediction method: Gnomon. Supporting evidence
                     includes similarity to: 1 Protein"
                     /transcript_id="XM_064275038.1"
                     /db_xref="GeneID:100669470"
     CDS             complement(<1..2)
                     /gene="ABCB8"
                     /note="Derived by automated computational analysis using
                     gene prediction method: Gnomon."
                     /codon_start=1
                     /product="mitochondrial potassium channel ATP-binding
                     subunit isoform X2"
                     /protein_id="XP_064131108.1"
                     /db_xref="GeneID:100669470"
                     /translation="MLVHLFRVGIRGGPAPGRPLLLRLQTFTAVRHSDGHHSSNLLRS
                     VARLRSQLQPRLPRAPPAPSRSPLSAWHWVGGILLGPVVLRKCSHLCLVARCEAEEAP
                     PVSSRPYVEESHFNWKLFWQFLRPHLLVLGVAIVLALGAALVNVQIPLLLGQLVEIVA
                     KYTREHAGSFMTESRSLGTRLLLLYGIQGLLTFGYLVLLSRIGERMAVDMRRTLFCSL
                     LRQDIAFFDAKKTGQLVSRLTTDVQEFKSSFKLVISQGLRSCTQVAGCLVSLSMLSTR
                     LTLLLMVATPTLMGVGTLMGSALRKLSRQSQEQIARATGVADEALGNVRTVRAFAMEQ
                     REEERYGAELEASCCKAEELGRGIALFQGLSNIAFNCMVLGTLFIGGSLVAGQELTGG
                     DLMSFLVASQTVQRSMANLSILFGQVVRGLSAGARVFEYMTLSPCIPLTGGCCIPKED
                     LHGTITFQNVGFSYPCRPGFEVLKDFTLTLPPGKTVALVGQSGGGKTTVASLLERFYD
                     PTAGVVLLDGRDLRTLDPSWLRGQVIGFISQEPVLFGTTIMENIRFGKLEASDEEVYA
                     AAREANAHDFITSFPDGYGTVVGWDP"
     gene            1..12488
                     /gene="ATG9B"
                     /note="autophagy related 9B; Derived by automated
                     computational analysis using gene prediction method:
                     Gnomon."
                     /db_xref="GeneID:100669187"
     mRNA            join(1..68,2386..2939,3073..3118,3300..3362,3578..3739,
                     4528..4669,8385..9139,9242..9395,9558..9737,10239..10475,
```

```
                     10667..10800,10969..11065,11256..11379,11465..12488)
                     /gene="ATG9B"
                     /product="autophagy related 9B"
                     /note="Derived by automated computational analysis using
                     gene prediction method: Gnomon. Supporting evidence
                     includes similarity to: 10 Proteins"
                     /transcript_id="XM_064274259.1"
                     /db_xref="GeneID:100669187"
     CDS             join(1..68,2386..2939,3073..3118,3300..3362,3578..3739,
                     4528..4669,8385..9139,9242..9395,9558..9737,10239..10475,
                     10667..10800,10969..11065,11256..11379,11465..11592)
                     /gene="ATG9B"
                     /note="Derived by automated computational analysis using
                     gene prediction method: Gnomon."
                     /codon_start=1
                     /product="autophagy-related protein 9B"
                     /protein_id="XP_064130329.1"
                     /db_xref="GeneID:100669187"
                     /translation="MMGRERMSSQQQRFHTRPTEGSIEATSLTVSQMGWGGNRGRLGR
                     WGDLGSGSVPLLPTPSPPPRPPSCWRPGGGRISIFSLSSAPHTRSSPSAFLPPGLGSH
                     CPVLQVLGPSQPHHSALSTPTWAQPMKTSTSASPSWGSHSIPPLASVTPPPSRRCPQD
                     PPGLRMGPLIPEQDYERLEDCDPEGSQDSPLHGEEQQPLLHVPEGPRGSWHHIQNLDS
                     FFTKIYNYHQRNGFACILLEDVFQLGQFVFIVTFTTFLICCVDYNVLFANQPNNHTTS
                     GPLHSKVTLSDAILSSAQCAERIRSSPLLVFLLVLASGFWLFQLLRSVCNLFSYWDIR
                     VFYREALHIPPEELSSMPWAEVQSRLLALQRSGGLCVQPRPLTELDVHHRILRYTNYQ
                     VALANKGLLPARCPLPCGGSVAFLSRGLALNVDLLLFRGPFSLFRGGWELPDAYKRSE
                     QRGALAARLGRTALLLAVANLALSPLVLAWQVLHAFYSHAELLRREPGALGARRWSRL
                     ARLQLRHFNELPHELRARLARAYRPAAAFLRAAAQPAPLLELLARQVVFYAGALFAAL
                     LLLTVYDEDVLSVQHVLAATTVLGVTATVARSFLPEEQLQGRSAPLLLQAALAHMHYL
                     PEEPGLASQAGAYRQMAQLLQYRAVSLLEELLSPLLAPLFLYFWFRPRALEIIDFFHH
                     FTVDVAGVGDICSFALMDVKRHGHPQWLSAGQTEASLAQRAEDGKTELSLMRFSLAHP
                     QWRPPGHSSKFLGHLWGRVQQDAATWGATSVRSPTTPGLLSDSASSLPEAFLANLLVH
                     PLLPPRDLSPTAPCPAAATASLLASISRIPQDPSCVSPGGTGGQKLTQLPDLASAEMS
                     LHAIYLHQLHQQQQQELWGEVSASSLSRPWSSPSHPPSPDEEKPSWSSDGSSPASSPR
                     QQWRTQTAQNLFPGGIQETMETQQEPGQAPSTD"
```

(M) Cattle

```
LOCUS       NC_037331               9849 bp    DNA     linear   CON 26-DEC-2024
DEFINITION  Bos taurus isolate L1 Dominette 01449 registration number 42190680
            breed Hereford chromosome 4, ARS-UCD2.0, whole genome shotgun
            sequence.
ACCESSION   NC_037331 REGION: complement(113593467..113603315)
VERSION     NC_037331.1
DBLINK      BioProject: PRJNA450837
            BioSample: SAMN03145444
            Assembly: GCF_002263795.3
KEYWORDS    WGS; RefSeq.
SOURCE      Bos taurus (domestic cattle)
  ORGANISM  Bos taurus
            Eukaryota; Metazoa; Chordata; Craniata; Vertebrata; Euteleostomi;
            Mammalia; Eutheria; Laurasiatheria; Artiodactyla; Ruminantia;
            Pecora; Bovidae; Bovinae; Bos.
COMMENT     REFSEQ INFORMATION: The reference sequence is identical to
            CM008171.2.
            Assembly name: ARS-UCD2.0
            The genomic sequence for this RefSeq record is from the
            whole-genome assembly released by the USDA ARS on 2018/04/11. The
            original whole-genome shotgun project has the accession
            NKLS00000000.2.

            ##Genome-Assembly-Data-START##
            Assembly Provider      :: USDA ARS
            Assembly Date          :: DEC-2017
            Assembly Method        :: Falcon v. FEB-2016
            Genome Representation  :: Full
            Expected Final Version :: Yes
            Genome Coverage        :: 80.0x
            Sequencing Technology  :: PacBio; Illumina NextSeq 500; Illumina
                                      HiSeq; Illumina GAII
            ##Genome-Assembly-Data-END##

            ##Genome-Annotation-Data-START##
            Annotation Provider         :: NCBI RefSeq
            Annotation Status           :: Updated annotation
            Annotation Name             :: GCF_002263795.3-RS_2024_12
            Annotation Pipeline         :: NCBI eukaryotic genome annotation
                                           pipeline
```

```
            Annotation Software Version :: 10.3
            Annotation Method           :: Best-placed RefSeq; Gnomon;
                                           cmsearch; tRNAscan-SE
            Features Annotated          :: Gene; mRNA; CDS; ncRNA
            Annotation Date             :: 12/22/2024
            ##Genome-Annotation-Data-END##
FEATURES             Location/Qualifiers
     source          1..9849
                     /organism="Bos taurus"
                     /mol_type="genomic DNA"
                     /isolate="L1 Dominette 01449 registration number 42190680"
                     /db_xref="taxon:9913"
                     /chromosome="4"
                     /sex="female"
                     /tissue_type="left lung"
                     /breed="Hereford"
     gene            1..9849
                     /gene="ATG9B"
                     /note="autophagy related 9B; Derived by automated
                     computational analysis using gene prediction method:
                     Gnomon."
                     /db_xref="BGD:BT19074"
                     /db_xref="GeneID:506862"
                     /db_xref="VGNC:VGNC:26263"
     mRNA            join(1..550,706..749,893..957,1080..1241,1785..1926,
                     3464..4218,4384..4537,4672..4851,5555..5791,5953..6086,
                     6239..6335,6533..6659,6745..6887,7044..8686,8992..9849)
                     /gene="ATG9B"
                     /product="autophagy related 9B"
                     /experiment="COORDINATES: polyA evidence [ECO:0006239]"
                     /transcript_id="XM_010804677.3"
                     /db_xref="GeneID:506862"
                     /db_xref="BGD:BT19074"
                     /db_xref="VGNC:VGNC:26263"
     CDS             join(1..550,706..749,893..957,1080..1241,1785..1926,
                     3464..4218,4384..4537,4672..4851,5555..5791,5953..6086,
                     6239..6335,6533..6659,6745..6872)
                     /gene="ATG9B"
                     /note="Derived by automated computational analysis using
                     gene prediction method: Gnomon."
                     /codon_start=1
                     /product="autophagy-related protein 9B"
                     /protein_id="XP_010802979.1"
                     /db_xref="GeneID:506862"
                     /db_xref="BGD:BT19074"
                     /db_xref="VGNC:VGNC:26263"
                     /translation="MVRRMGLGGTRGRLGRWGDLGPGSVPLLPTPLPPLPPPPCRGPG
                     AGRVSFFSLSPVPPTRRAPSSASPSASGLPCPAVQAPGASQPCHSALPTPATPPVQPQ
                     PLMTPVSAPPLWGSHSAPPPASGTPPPPRRCPQDSPGLRIGPLIPEQDYEQLEDGDPE
                     GSQDSPLHGEEQQPLLHVPEGLRGSWHHIQNLDSFFTKIYSYHQRNGFTCILLEDVFQ
                     LGQFIFIVTFTTFLLRCVDYSVLFANQLSNRTRPEPLHSKVTLSDAVLPSSQCAQRIC
                     SSPLLVFLLILAAAFWLFQLLRSVCNLFSYWDIQVFYREALHIPPEELSSVPWAEVQS
                     RLLALQRSGGLCVQPRPLTELDVHHRILRYTNYQVALANKGLLPARAALPWGGGAAFL
                     SRGLALNVDLLLFRGPFSLFRGGWELPDSYKRGDRRAALAARWRRTVLLLAAANLALS
                     PLVLAWQVLQAFYSHAELLRREPGALGTRRWSRLARLQLRHFNELPHELRARLARAYR
                     PAAAFLRAAAPPAPLLALLARQLVFFAGAPLAALLVLTVYDEDVLAVEHVLTAMTALG
                     IAATVARSFIPDEQGQGRSPQLLLQAALAHMHYLPEETGPAGRTSSYRQMARLLQYRA
                     VSLVEELLSPVLTPLFLIFWFRPRALEIIDFFRHFTVDVAGVGDICSFALMDVKRHGH
                     PQWLSEGQTEASLSQRAEDGKTELSLMRFSLVHPQWRPPGHSSKFLGHLRGRVQQDAA
                     SWGASSLRSPPAPGTLGNSPSPLPEAFLANLLVQPLRPPQDLSPTAPCPAAATASLLA
                     SLSRITQDSSCVSPGGTGGQKLAQLPELASAEMSLHAIYLHQLHQQQQQELWGEASA
                     SPLSRAWPSPPQTLSPDEEKPCWSSDGSSPASSPRQQWSAQRTQSSFPGGFQEPTDTQ
                     KEPGQATSTD"
     gene            complement(7789..>9849)
                     /gene="NOS3"
                     /note="nitric oxide synthase 3; Derived by automated
                     computational analysis using gene prediction method:
                     BestRefSeq,Gnomon."
                     /db_xref="BGD:BT18122"
                     /db_xref="GeneID:287024"
                     /db_xref="VGNC:VGNC:32174"
     mRNA            complement(join(7789..8382,8502..8696,9027..>9175))
                     /gene="NOS3"
                     /product="nitric oxide synthase 3"
                     /inference="similar to RNA sequence, mRNA (same
                     species):RefSeq:NM_181037.3"
                     /exception="annotated by transcript or proteomic data"
                     /note="The RefSeq transcript has 6 substitutions compared
```

```
                     to this genomic sequence; Derived by automated
                     computational analysis using gene prediction method:
                     BestRefSeq."
                     /transcript_id="NM_181037.3"
                     /db_xref="GeneID:287024"
                     /db_xref="BGD:BT18122"
                     /db_xref="VGNC:VGNC:32174"
     mRNA            complement(join(7789..8382,8502..8696,9027..>9175))
                     /gene="NOS3"
                     /product="nitric oxide synthase 3, transcript variant X1"
                     /experiment="COORDINATES: cap analysis [ECO:0007248] and
                     polyA evidence [ECO:0006239]"
                     /transcript_id="XM_024990490.2"
                     /db_xref="GeneID:287024"
                     /db_xref="BGD:BT18122"
                     /db_xref="VGNC:VGNC:32174"
     CDS             complement(join(8221..8382,8502..8696,9027..>9175))
                     /gene="NOS3"
                     /note="Derived by automated computational analysis using
                     gene prediction method: BestRefSeq."
                     /codon_start=1
                     /product="nitric oxide synthase, endothelial"
                     /protein_id="NP_851380.2"
                     /db_xref="GeneID:287024"
                     /db_xref="BGD:BT18122"
                     /db_xref="VGNC:VGNC:32174"
                     /translation="MGNLKSVGQEPGPPCGLGLGLGLGLCGKQGPASPAPEPSRAPAP
                     ATPHAPDHSPAPNSPTLTRPPEGPKFPRVKNWELGSITYDTLCAQSQQDGPCTPRRCL
                     GSLVLPRKLQTRPSPGPPPAEQLLSQARDFINQYYSSIKRSGSQAHEERLQEVEAEVA
                     STGTYHLRESELVFGAKQAWRNAPRCVGRIQWGKLQVFDARDCSSAQEMFTYICNHIK
                     YATNRGNLRSAITVFPQRAPGRGDFRIWNSQLVRYAGYRQQDGSVRGDPANVEITELC
                     IQHGWTPGNGRFDVLPLLLQAPDEAPELFVLPPELVLEVPLEHPTLEWFAALGLRWYA
                     LPAVSNMLLEIGGLEFSAAPFSGWYMSTEIGTRNLCDPHRYNILEDVAVCMDLDTRTT
                     SSLWKDKAAVEINLAVLHSFQLAKVTIVDHHAATVSFMKHLDNEQKARGGCPADWAWI
                     VPPISGSLTPVFHQEMVNYILSPAFRYQPDPWKGSATKGAGITRKKTFKEVANAVKIS
                     ASLMGTLMAKRVKATILYASETGRAQSYAQQLGRLFRKAFDPRVLCMDEYDVVSLEHE
                     ALVLVVTSTFGNGDPPENGESFAAALMEMSGPYNSSPRPEQHKSYKIRFNSVSCSDPL
                     VSSWRRKRKESSNTDSAGALGTLRFCVFGLGSRAYPHFCAFARAVDTRLEELGGERLL
                     QLGQGDELCGQEEAFRGWAKAAFQASCETFCVGEEAKAAAQDIFSPKRSWKRQRYRLS
                     TQAEGLQLLPGLIHVHRRKMFQATVLSVENLQSSKSTRATILVRLDTAGQEGLQYQPG
                     DHIGICPPNRPGLVEALLSRVEDPPPPTESVAVEQLEKGSPGGPPPSWVRDPRLPPCT
                     LRQALTFFLDITSPPSPRLLRLLSTLAEEPSEQQELETLSQDPRRYEEWKWFRCPTLL
                     EVLEQFPSVALPAPLLLTQLPLLQPRYYSVSSAPNAHPGEVHLTVAVLAYRTQDGLGP
                     LHYGVCSTWLSQLKTGDPVPCFIRGAPSFRLPPDPYVPCILVGPGTGIAPFRGFWQER
                     LHDIESKGLQPAPMTLVFGCRCSQLDHLYRDEVQDAQERGVFGRVLTAFSREPDSPKT
                     YVQDILRTELAAEVHRVLCLERGHMFVCGDVTMATSVLQTVQRILATEGDMELDEAGD
                     VIGVLRDQQRYHEDIFGLTLRTQEVTSRIRTQSFSLQERHLRGAVPWAFDPPGPDTPG
                     P"
     CDS             complement(join(8221..8382,8502..8696,9027..>9175))
                     /gene="NOS3"
                     /note="Derived by automated computational analysis using
                     gene prediction method: Gnomon."
                     /codon_start=1
                     /product="nitric oxide synthase 3 isoform X1"
                     /protein_id="XP_024846258.1"
                     /db_xref="GeneID:287024"
                     /db_xref="BGD:BT18122"
                     /db_xref="VGNC:VGNC:32174"
                     /translation="MFTYICNHIKYATNRGNLRSAITVFPQRAPGRGDFRIWNSQLVR
                     YAGYRQQDGSVRGDPANVEITELCIQHGWTPGNGRFDVLPLLLQAPDEAPELFVLPPE
                     LVLEVPLEHPTLEWFAALGLRWYALPAVSNMLLEIGGLEFSAAPFSGWYMSTEIGTRN
                     LCDPHRYNILEDVAVCMDLDTRTTSSLWKDKAAVEINLAVLHSFQLAKVTIVDHHAAT
                     VSFMKHLDNEQKARGGCPADWAWIVPPISGSLTPVFHQEMVNYILSPAFRYQPDPWKG
                     SATKGAGITRKKTFKEVANAVKISASLMGTLMAKRVKATILYASETGRAQSYAQQLGR
                     LFRKAFDPRVLCMDEYDVVSLEHEALVLVVTSTFGNGDPPENGESFAAALMEMSGPYN
                     SSPRPEQHKSYKIRFNSVSCSDPLVSSWRRKRKESSNTDSAGALGTLRFCVFGLGSRA
                     YPHFCAFARAVDTRLEELGGERLLQLGQGDELCGQEEAFRGWAKAAFQASCETFCVGE
                     EAKAAAQDIFSPKRSWKRQRYRLSTQAEGLQLLPGLIHVHRRKMFQATVLSVENLQSS
                     KSTRATILVRLDTAGQEGLQYQPGDHIGICPPNRPGLVEALLSRVEDPPPPTESVAVE
                     QLEKGSPGGPPPSWVRDPRLPPCTLRQALTFFLDITSPPSPRLLRLLSTLAEEPSEQQ
                     ELETLSQDPRRYEEWKWFRCPTLLEVLEQFPSVALPAPLLLTQLPLLQPRYYSVSSAP
                     NAHPGEVHLTVAVLAYRTQDGLGPLHYGVCSTWLSQLKTGDPVPCFIRGAPSFRLPPD
                     PYVPCILVGPGTGIAPFRGFWQERLHDIESKGLQPAPMTLVFGCRCSQLDHLYRDEVQ
                     DAQERGVFGRVLTAFSREPDSPKTYVQDILRTELAAEVHRVLCLERGHMFVCGDVTMA
                     TSVLQTVQRILATEGDMELDEAGDVIGVLRDQQRYHEDIFGLTLRTQEVTSRIRTQSF
                     SLQERHLRGAVPWAFDPPGPDTPGP"
```

(N) Grey whale

```
LOCUS       NC_090831               7599 bp    DNA     linear   CON 19-SEP-2024
DEFINITION  Eschrichtius robustus isolate mEscRob2 chromosome 8, mEscRob2.pri,
            whole genome shotgun sequence.
ACCESSION   NC_090831 REGION: complement(124314329..124321927)
VERSION     NC_090831.1
DBLINK      BioProject: PRJNA1160892
            BioSample: SAMN32629250
            Assembly: GCF_028021215.1
KEYWORDS    WGS; RefSeq.
SOURCE      Eschrichtius robustus (grey whale)
  ORGANISM  Eschrichtius robustus
            Eukaryota; Metazoa; Chordata; Craniata; Vertebrata; Euteleostomi;
            Mammalia; Eutheria; Laurasiatheria; Artiodactyla; Whippomorpha;
            Cetacea; Mysticeti; Eschrichtiidae; Eschrichtius.
COMMENT     REFSEQ INFORMATION: The reference sequence is identical to
            CM051672.1.
            Assembly name: mEscRob2.pri
            The genomic sequence for this RefSeq record is from the
            whole-genome assembly released by the Vertebrate Genomes Project on
            2023/01/31. The original whole-genome shotgun project has the
            accession JAQMHY000000000.1.

            ##Genome-Assembly-Data-START##
            Assembly Provider      :: Vertebrate Genomes Project
            Assembly Date          :: 01-DEC-2022
            Assembly Method        :: HiFiasm v. 0.16.1 + galaxy3; yahs v. 1.2a
                                      + galaxy2
            Assembly Name          :: mEscRob2.pri
            Diploid                :: Principal Haplotype
            Genome Representation  :: Full
            Expected Final Version :: No
            Genome Coverage        :: 29.61x
            Sequencing Technology  :: PacBio Sequel II HiFi; 3D-DNA Hi-C
            ##Genome-Assembly-Data-END##

            ##Genome-Annotation-Data-START##
            Annotation Provider         :: NCBI RefSeq
            Annotation Status           :: Full annotation
            Annotation Name             :: GCF_028021215.1-RS_2024_09
            Annotation Pipeline         :: NCBI eukaryotic genome annotation
                                           pipeline
            Annotation Software Version :: 10.3
            Annotation Method           :: Gnomon; cmsearch; tRNAscan-SE
            Features Annotated          :: Gene; mRNA; CDS; ncRNA
            Annotation Date             :: 09/18/2024
            ##Genome-Annotation-Data-END##
FEATURES             Location/Qualifiers
     source          1..7599
                     /organism="Eschrichtius robustus"
                     /mol_type="genomic DNA"
                     /isolate="mEscRob2"
                     /db_xref="taxon:9764"
                     /chromosome="8"
                     /sex="male"
                     /cell_line="mEscRob2 fibroblast"
                     /tissue_type="Cells grown from a skin dart biopsy of
                     epidermis and blubber"
                     /dev_stage="adult"
                     /geo_loc_name="USA: North Pacific Ocean, near Crescent
                     City, California"
                     /lat_lon="41.8349 N 124.2969 W"
                     /collection_date="2016-03-18"
                     /collected_by="Jeffrey K. Jacobsen"
     gene            1..7599
                     /gene="ATG9B"
                     /note="autophagy related 9B; Derived by automated
                     computational analysis using gene prediction method:
                     Gnomon."
                     /db_xref="GeneID:137768632"
     mRNA            join(1..538,665..708,854..918,1105..1266,1789..1930,
                     3465..4219,4384..4537,4717..4896,5616..5852,6018..6151,
                     6331..6427,7001..7116,7200..7353,7511..7599)
                     /gene="ATG9B"
                     /product="autophagy related 9B, transcript variant X2"
                     /note="Derived by automated computational analysis using
                     gene prediction method: Gnomon. Supporting evidence
                     includes similarity to: 13 Proteins"
```

```
                     /transcript_id="XM_068550187.1"
                     /db_xref="GeneID:137768632"
     mRNA            join(1..538,665..708,854..918,1105..1266,1789..1930,
                     3465..4219,4384..4537,4717..4896,5616..5852,6018..6151,
                     6331..6427,7001..7116,7200..7402)
                     /gene="ATG9B"
                     /product="autophagy related 9B, transcript variant X1"
                     /note="Derived by automated computational analysis using
                     gene prediction method: Gnomon. Supporting evidence
                     includes similarity to: 13 Proteins"
                     /transcript_id="XM_068550184.1"
                     /db_xref="GeneID:137768632"
     CDS             join(1..538,665..708,854..918,1105..1266,1789..1930,
                     3465..4219,4384..4537,4717..4896,5616..5852,6018..6151,
                     6331..6427,7001..7116,7200..7335)
                     /gene="ATG9B"
                     /note="Derived by automated computational analysis using
                     gene prediction method: Gnomon."
                     /codon_start=1
                     /product="autophagy-related protein 9B"
                     /protein_id="XP_068406285.1"
                     /db_xref="GeneID:137768632"
                     /translation="MVRQMGGGWGGVWTTGRLGRWGDLGRGSVPLLPVPLPPLPPPPG
                     RGPGGGRVSIFSLAPAPHTKSTPSSAPSSAPGPPFPAVQAPGASQPRSSTLPTPATPA
                     TQPRPAMTPISAPPSWGSHSAPPPRRCPQDPPGLRIGPLVPEQVYERLDDCDPEGSQD
                     SALHGEEQQPLLHVPEGLRGSWHHVQNLDSFFTKIYSYHQRSGFACILLEDVFQLGQF
                     IFIVIFTTFLLRCVDYSVLFANQPKNRTRPGSLHSKVTLSDAIVPSSQCAQRIRSSPL
                     LVFLLILAAAFWLFQLLRSVCKLFSYWDIRVFYREALRMPPEELSSVPWAEVQSRLLA
                     LQRSGGLCVQPRPLTELDVHHRILRYTNYQVALANKGLLPARCAVPWGGSAAFLSRGL
                     ALNVDLLLFRGPFSFFRGGWELPDAYKRSDRRAALAARWRRTVLLLAAVNLALSPLVL
                     AWQLLHAFYSRAEVLRREPGALGTRRWSRLARLQLRHFNELPHELRARLARAYCPAAA
                     FLRAAAPPAPLLALLARQLLFFAGALFAALLVLTVYDEDVLSVEHVLTAMTALGIVAT
                     VARSFIPGEQGQGRPPQILLPAALAHMHYLPEETGPAGRTSAYRQMARLLQYRAVSLA
                     EELLSPLLTPLFLLFWFRPRALEIIDFFRHFTVDVAGVGDICSFALMDVKRHGHPQWL
                     SEGQTEASLSERAEDGKTELSLMRFSLLHPQWCPPGHSSKFLGHLRGRVQQDAAAWDA
                     SSVRSPPSPGILSNSASPLQEAFPANLSMQPLGPPQDLSPIVPCPAAATASLLASISR
                     MTQDSSCVSPGGTGGQKLAQLPEVASADMSLHAIYLHQLHRQQQQELWGEASASSLSR
                     PWSSLLQTLSPEEEKPSWSSPGSSPASSPRQQWRTQRTRNLFPGGFQEPTDTQKEPGQ
                     ATSTD"
     CDS             join(1..538,665..708,854..918,1105..1266,1789..1930,
                     3465..4219,4384..4537,4717..4896,5616..5852,6018..6151,
                     6331..6427,7001..7116,7200..7335)
                     /gene="ATG9B"
                     /note="Derived by automated computational analysis using
                     gene prediction method: Gnomon."
                     /codon_start=1
                     /product="autophagy-related protein 9B"
                     /protein_id="XP_068406288.1"
                     /db_xref="GeneID:137768632"
                     /translation="MVRQMGGGWGGVWTTGRLGRWGDLGRGSVPLLPVPLPPLPPPPG
                     RGPGGGRVSIFSLAPAPHTKSTPSSAPSSAPGPPFPAVQAPGASQPRSSTLPTPATPA
                     TQPRPAMTPISAPPSWGSHSAPPPRRCPQDPPGLRIGPLVPEQVYERLDDCDPEGSQD
                     SALHGEEQQPLLHVPEGLRGSWHHVQNLDSFFTKIYSYHQRSGFACILLEDVFQLGQF
                     IFIVIFTTFLLRCVDYSVLFANQPKNRTRPGSLHSKVTLSDAIVPSSQCAQRIRSSPL
                     LVFLLILAAAFWLFQLLRSVCKLFSYWDIRVFYREALRMPPEELSSVPWAEVQSRLLA
                     LQRSGGLCVQPRPLTELDVHHRILRYTNYQVALANKGLLPARCAVPWGGSAAFLSRGL
                     ALNVDLLLFRGPFSFFRGGWELPDAYKRSDRRAALAARWRRTVLLLAAVNLALSPLVL
                     AWQLLHAFYSRAEVLRREPGALGTRRWSRLARLQLRHFNELPHELRARLARAYCPAAA
                     FLRAAAPPAPLLALLARQLLFFAGALFAALLVLTVYDEDVLSVEHVLTAMTALGIVAT
                     VARSFIPGEQGQGRPPQILLPAALAHMHYLPEETGPAGRTSAYRQMARLLQYRAVSLA
                     EELLSPLLTPLFLLFWFRPRALEIIDFFRHFTVDVAGVGDICSFALMDVKRHGHPQWL
                     SEGQTEASLSERAEDGKTELSLMRFSLLHPQWCPPGHSSKFLGHLRGRVQQDAAAWDA
                     SSVRSPPSPGILSNSASPLQEAFPANLSMQPLGPPQDLSPIVPCPAAATASLLASISR
                     MTQDSSCVSPGGTGGQKLAQLPEVASADMSLHAIYLHQLHRQQQQELWGEASASSLSR
                     PWSSLLQTLSPEEEKPSWSSPGSSPASSPRQQWRTQRTRNLFPGGFQEPTDTQKEPGQ
                     ATSTD"
```

(O) Common bottlenose dolphin

```
LOCUS       NC_047042               9376 bp    DNA     linear   CON 07-MAY-2025
DEFINITION  Tursiops truncatus isolate mTurTru1 chromosome 9, mTurTru1.mat.Y,
            whole genome shotgun sequence.
ACCESSION   NC_047042 REGION: complement(99227014..99236389)
VERSION     NC_047042.1
DBLINK      BioProject: PRJNA625792
            BioSample: SAMN12326775
            Assembly: GCF_011762595.2
KEYWORDS    WGS; RefSeq.
```

```
SOURCE      Tursiops truncatus (common bottlenose dolphin)
  ORGANISM  Tursiops truncatus
            Eukaryota; Metazoa; Chordata; Craniata; Vertebrata; Euteleostomi;
            Mammalia; Eutheria; Laurasiatheria; Artiodactyla; Whippomorpha;
            Cetacea; Odontoceti; Delphinidae; Tursiops.
COMMENT     REFSEQ INFORMATION: The reference sequence is identical to
            CM022282.1.
            This is the trio assembly merged haplotype data. The maternal and
            paternal haplotype sequences are in WGS projects JAAOMB000000000
            and JAAOMC000000000 respectively.

            ##Genome-Assembly-Data-START##
            Assembly Provider      :: Vertebrate Genomes Project
            Assembly Date          :: 15-JAN-2020
            Assembly Method        :: TrioCanu v. 1.8; purge_dups v. 1.0.0;
                                      Scaff 10x v. 4.1.0; Bionano solve v.
                                      3.2.1_04122018; Salsa2 HiC v. 2.2; Arrow
                                      polishing and gap filling v.
                                      smrtlink_6.0.0.47841; Freebayes v. 1.3.1;
                                      gEVAL manual curation v. 2020-01-15; VGP
                                      trio assembly pipeline v. 1.6
            Assembly Name          :: mTurTru1.mat.Y
            Genome Representation  :: Full
            Expected Final Version :: No
            Genome Coverage        :: 63.7x
            Sequencing Technology  :: PacBio Sequel I CLR; Illumina NovaSeq;
                                      Arima Genomics Hi-C; Bionano Genomics DLS
            ##Genome-Assembly-Data-END##

            ##Genome-Annotation-Data-START##
            Annotation Provider         :: NCBI RefSeq
            Annotation Status           :: Full annotation
            Annotation Name             :: GCF_011762595.2-RS_2025_04
            Annotation Pipeline         :: NCBI eukaryotic genome annotation
                                           pipeline
            Annotation Software Version :: 10.3
            Annotation Method           :: Best-placed RefSeq; Gnomon;
                                           cmsearch; tRNAscan-SE
            Features Annotated          :: Gene; mRNA; CDS; ncRNA
            Annotation Date             :: 04/30/2025
            ##Genome-Annotation-Data-END##
FEATURES             Location/Qualifiers
     source          1..9376
                     /organism="Tursiops truncatus"
                     /mol_type="genomic DNA"
                     /isolate="mTurTru1"
                     /db_xref="taxon:9739"
                     /chromosome="9"
                     /sex="male"
                     /tissue_type="spleen"
                     /geo_loc_name="USA: Baltimore, Maryland"
                     /lat_lon="39.2851 N 76.6083 W"
                     /collection_date="11-Nov-2018"
                     /collected_by="Leigh Clayton, Jill Arnold, Winston Timp,
                     Norah Hilger"
     gene            1..9376
                     /gene="ATG9B"
                     /note="autophagy related 9B; Derived by automated
                     computational analysis using gene prediction method:
                     Gnomon."
                     /db_xref="GeneID:117313525"
     mRNA            join(1..5,2112..2607,2722..2765,2911..2975,3167..3338,
                     3841..3975,5736..6524,6641..6749,6930..7021,7820..7943,
                     8030..9376)
                     /gene="ATG9B"
                     /product="autophagy related 9B"
                     /experiment="COORDINATES: polyA evidence [ECO:0006239]"
                     /exception="unclassified transcription discrepancy"
                     /note="The sequence of the model RefSeq transcript was
                     modified relative to this genomic sequence to represent
                     the inferred CDS: inserted 4 bases in 3 codons; Derived by
                     automated computational analysis using gene prediction
                     method: Gnomon. Supporting evidence includes similarity
                     to: 2 Proteins"
                     /transcript_id="XM_073809474.1"
                     /db_xref="GeneID:117313525"
     CDS             join(1..5,2112..2607,2722..2765,2911..2975,3167..3338,
                     3841..3975,5736..6524,6641..6749,6930..7021,7820..7943,
```

```
                     8030..8157)
                     /gene="ATG9B"
                     /exception="unclassified translation discrepancy"
                     /note="The sequence of the model RefSeq protein was
                     modified relative to this genomic sequence to represent
                     the inferred CDS: inserted 4 bases in 3 codons;
                     substituted 2 bases at 2 genomic stop codons; Derived by
                     automated computational analysis using gene prediction
                     method: Gnomon."
                     /codon_start=1
                     /transl_except=(pos:complement(99233058..99233060),
                     aa:OTHER)
                     /transl_except=(pos:complement(99230371..99230373),
                     aa:OTHER)
                     /product="LOW QUALITY PROTEIN: autophagy-related protein
                     9B"
                     /protein_id="XP_073665575.1"
                     /db_xref="GeneID:117313525"
                     /translation="MGWAGGGDRGQLGRGSVPLLPVPLPPLSPPPGRGPGGGRVSIFS
                     LAPAPHTKSTPSSAPPSAPGPPYPAVQAPGASQLRYSTLPTPATPATQXRPAMMPISA
                     PHSWGSHPAPAPCRCPQDPPGLRRGPLIPEQDSERLEDCDPEGSQDSALHGEGQQPLL
                     HVPEGLRGSWHHVQNLDSFFTKMYSYHQRSGFACILLEDVFQLGRFIFIVTFTTFLLR
                     CVDYSVLFANQPKNRTRPGSLHSKVTLSDANLPSSQCAQRXVTDPLLLVFLLILAAAV
                     WLFQLLCSVCKLFSYWDIQVFSREALHIPPEELSSVPWAEVQSRLLALQRSGGLCGQP
                     RPLTELDAHHRILRYTNYQVALASKGLLPARRAVPRGGRAAFLSCGLAPNXDLLLSRG
                     PFSFFRGGXELPDAYKRSDRRAALAARWRRTVLLLAAVNLALSPLVLAWQVLHALYSR
                     AELLRREPGALATRRWSRLARQQLRHFNELPHELRARLARASRPAAAFLRAPAPVLAL
                     LARQLVFFAGALFAALLVLTVYYKDVLYVEHVLTAMTALGIVASGQVRWGRCPGRGIL
                     LSWGLSFRAGRAKVVRRKPCPAAATASLLASISRMTQDSSCVSPGGSGGQKLAQLPEL
                     VSAEXLHTIYLHQLHRQQQQELWGEASAFSLSRPWASPPQTLSPDEEKPSWSSDGSSP
                     ASSPRQQWRTQRTQNLFPGGFQEPTDTQKEAGQATSTD"
ORIGIN
        1 atggggtgac gttttgaggg tgtgcagagt gcgtcccccc gccctggggt atccatagta
       61 acgcgtcagc tgctgacctc cgggtcattc tcttatgggc gctcctggcg gactctagat
      121 gttccggagt ctccagggag tgtagaccgc tggttctggg agactaaggg acaaaaaccg
      181 tttggagttt tttcaaaaca tgtgcacgtg ctttgccacc caccccgctc tcctaggcga
      241 gtcagagctc aggggacggc agccacatat atttgggaga aaaaaaaccc tggtgattct
      301 gataagctgc ccctactcaa tggtttagaa ccgctggcgc gaccctaaat ccatctcccc
      361 cacagatgcc ttcctaccgc agttaggctg actgtgcctg aagctgccac cgcacagctc
      421 ttacacctct gtttcactct tagttatttc ttctgtgtaa ttacttgttt gcatagctgc
      481 ctcccctctg ggctgtgact tctttttggg tagtggctct gtctcattca tccgtcccct
      541 ccttcccctc caaatgcctg gcagggaaga gtgtccttaa taataaattg acgtgaattg
      601 gggccggtgg tagggaggct gttcatcgat gaatctggag tgaaactggg taggaaaacg
      661 tgtcaggatt agtctgaggt tgggctgggt gaggaggctg tatgggaggg tcctaaagtg
      721 agtatggtgg aatatcaggg tgagtgaggc tggggagaga ccagaggaaa ccccacatat
      781 ctgattttga caggtgtctc agagagatca tgggaatttc ctagcccctc tgcagtgtgg
      841 gattccattt tgggcaccag gaatcttgtt ttcatagctc tgcctcctgc tcggggataa
      901 aactatgcaa gtcctgagct accatgacaa ttcccctccc aggaaggaag cacaggttcc
      961 tcctctaagt tcctccagtg ggcaatctgg gatgtactct gttcctctgt ttccatgtct
     1021 tggatactgg tccctacatc agcgcttggg cttaagccct ggaggccctg acacatgggt
     1081 ggctgagtca gcacctcagg aatggacttc agtaggaaac ctgatgattc agggcctcaa
     1141 gaatggcctt ggacaagcta ccataccagc tgcctgggcc tccgcctctt ccttgaggtg
     1201 gcaacaagga actcaactca gtcagctcat ccctggggca cccaaattac ctgcccttcc
     1261 aaatgcctga ccttcagctg ccctctgctg ggtcagccct gatgcatctc agggggctcc
     1321 caaaactgga tcagaacttg gtagaggggg tttcaagagg ataaataagc aaacatactg
     1381 gagcttccaa ggaaccccac tttcctactg taaaatctcc gagaaccacc tctcgtccca
     1441 aataactgcc tctcctccct tcacttgctc cctctcagtc tcccccaccc tggctgcaaa
     1501 gtcagacggt ctcccctttt ccattcctta gcactgtcct acaagcaaaa catgcagccg
     1561 ttctcgtttc cttttttctt ttttgtgttt ggtcttcaag tgtttctccg tggatcactc
     1621 tgtgtgtgtg tatataggga ggggagataa gttgttttgt tttattctga gataaggttt
     1681 taagaaacta aagatgtata tcttggcccc atagccctat aatcctaact agattctttc
     1741 tgtctttgtt ctcggcttct gctctctggc ctcccgtttt aagtccgctg gggtctgaa
     1801 ggtgggaccg gggggatctc cttccatctg ccgctagtct gctcctgcct ctccaggtat
     1861 tgccctgcca ggcatggagg cggggcccta agaggtttgg ccgcgggttg ccctggaagg
     1921 ggtgtgggtg gtcgggctgg tgactgcgag ttgggacgcc tgaggcctgc cccaactggg
     1981 tcaccgggct gttgttgtca ctgtcctggt tgctgaagtc cccgctcagc aacgcagacc
     2041 tcttcggaaa cagcagcagc tcccatcttt ccaagctttc aggagatgcc gctggcctga
     2101 tggtgaggca gatgggcggg gggtggggac cgggggcagc tggggcgtgg atccgtgccc
     2161 ctcctccccg tgccactgcc tcctctttct cctcctccgg gtcggggacc tgggggaggg
     2221 agggtctcca tcttctctct ggcccctgca cctcacacaa aaagcacgcc ttcctcagct
     2281 cctccttcag ccccggggcc cccctaccca gcggtgcagg ccccaggggc ttctcagctt
     2341 cgctatagta ccctccccac tcccgctacc cccgccacgc agcccgaccc gcgatgatgc
     2401 ccatctctgc tccccattcc tggggttccc accctgcccc tgccccatgc cgatgccccc
     2461 aggaccctcc tggctgcgg agaggccctc tgatccctga gcaggattct gagaggctgg
     2521 aggactgtga ccctgagggg tcccaagatt cagccctcca tggggagggg cagcagcccc
     2581 tgcttcacgt gcctgaaggg ctccgaggtg aggggctggg gagggcctca gggctccaga
     2641 gtgtcccctg ggctgggcct ctggcagagg cttgagggca cggaggcagg tgagtcccaa
     2701 cgctctcccc tctgtgccca ggctcctggc accacgtcca gaacctggac agcttcttca
```

2761 ccaagatatc cttttgtgct ctgcctggca gaaggggcac gggagacagg gagacaggga
2821 ggcagtgggc tggggacttg gggctgagg agtcggcgtc ctcaggaaag gcccatcatg
2881 cctcaggctt aattcttgac gccctgccac atgtacagct accaccagag gagtggcttt
2941 gcctgtatcc tgctggagga tgtcttccag ctggggtgag atgcttgtcc cagactccaa
3001 gctcttcccc ttgcttcctc agtagtgcct gctgcttctg ggcctccagt gccttcccag
3061 gcaacctagg ggctcagcag gatgggttaa tccgggcggg cccggaggct ggggcaggag
3121 ccctcccagc ccgcaggcct catcactgca ctccctcctg tcccagacga ttcattttca
3181 ttgtcacctt cacaaccttc cttcttcgct gcgtggatta cagcgttctc tttgccaacc
3241 aaccaaaaaa ccgaacaaga cctgggtcgc tccacagcaa agtgaccttg tcagatgcta
3301 acctaccctc atcccagtgt gcccagcggt gagtgacagc tggtggggga ggtgatgggg
3361 gagagcaact gacaaagaca aaagcctccc ctccttttgt tccttggttt agatcagcca
3421 ttctccaagt ttttagtctc aggccacctt tgatacactt aaaattgatt gagaacccaa
3481 ctcccgagac cttttgttga tgagaatcct atctagatcc accttgttag agattaaagc
3541 tgtccctaag aacggacaaa catacattcc atcgactgac cgtgatgtca cgtagccgct
3601 ggaaaaggcc actgcgtact cttgggagaa ggagaatgaa aatggcagaa aaccttaata
3661 ttatatgaaa agttttgact ttgcagacac tcacaagggt ctggggaccc ctagggccca
3721 cactttgaga accactggtc tggctgggtc ccctctccag gaccccccttg ctccccaagc
3781 caggtctccc ttgctatggg gccgctgctt tctctcttcc gtctgtcccg cctggcacag
3841 gatccgctcc tgcttgtctt cctgctgatc ctggcggctg ccgtctggct cttccagctg
3901 ctttgctcgg tctgcaaact cttcagctac tgggacatcc aggtgtttc cagggaggcc
3961 ctgcacatcc ccccagtgag ttgggtgagc gtgtctgtgg aggcgtgcca tgtcaggggg
4021 cggagggtgg gaaggagtag ggagctgtac tgacagaagg atccactggg aagagggcag
4081 aagcccttag agacacctac ccttggcccc gaataatgtt aggaggtggg gtaccgcccc
4141 ttagaacaca aggtccttcc tgttgtggag cttagttgat gtagatggtt atggaagatg
4201 atgcacagtc acgtgcttgt ggtccaaaca atgggtcttt agaggaaggg cagtcacctg
4261 actttccaga gcaggaggaa ctgggcttgg gctggggagg ataggaggcg gggaggcatt
4321 ccaaacccca ggcaggaagg gggcagggga agcaatgcgc atctttgggc agcagtaagg
4381 agactgaccc accagcctgc aactgagtgt tcaggccccc gtcagtgagc ccaccccccct
4441 ttctggcccc tgtttgcagg aagctacttc gtcggcccct tcactgtccc tggtcacgct
4501 ttgctctttc ctccccatct cgctcatgct ccttctggct ctgtacccac gttctttctt
4561 tctttctttt tttttttttc gcggtacgcg ggcctctcac tgttgtggcc tctcccgttg
4621 tggagcacag actccggacg cgcaggctca gtggccatgg ctcatgggcc cagccactcc
4681 acggcatgtg ggatcttccc ggaccggggc acgaacccgc gtcccctgca tcggcaggcg
4741 gactcccaac cactgcgcca ccagggaagc cccccaagtt ctttctttaa ggcacggcgc
4801 aaaggctact ttcagcagcc agctttctct gctcctctca gaagtgcaga gacttttaca
4861 cagccctcct gtgcagcaaa ggggagaggt tctcgaaagc ccacctgagt atggaagcac
4921 aagccatagc acaggaaaga gtcgcgaggg ctggggatgg aggcagggaa gctgaggagc
4981 tggaccactg ctcctccagg aggtgcctga cccctctttc tccttccttc tttcccctct
5041 tccttccagg ggaaagggag accctgggat ggactgcaga ggctggcctg gagggacttg
5101 actaagctgg actggggagg ctgggtgaaa ctgggctaaa atgaggtttg cagatgagct
5161 ccaagtgaga tctgacagcc tccaccccga cggggacaag gccactgtct taggaaggtt
5221 aatctgttcg agtgggatgg ggcggggtgt gtgtgtgtgt gtctcaaagc atgctgaggt
5281 aaaagtttgg cggcttccgg gcccagcggg ctggtacccc aggaacggga aagagactgc
5341 cttgttacct gcagctctga tgtaccttgt atgtgtaggg ctgcgggcac gggtgggcga
5401 gcggtggcca ggaaaacctg ctcttacgga gctgagctca gatacatgta gagttggttt
5461 atgagctctt cctgggaagc acatgactca cggcctcagg aggcgcaggg gcccgaggaa
5521 cacagttcag aagagggtga ggggaactaa gaggtgcccc ttgttggtgg caaggcgaag
5581 ggagggcgag ggggcagaaa gcgaaaggtg gcagctttcg tgcccgagag acactgggtg
5641 aggacggcgg ggatacatct ctggtcacgg ggagtttggg catcgccgtg gagaggaatc
5701 caggtggctg gcgctcaggc gtcccctctc cgcaggagga gctcagctcg gtgccctggg
5761 ccgaggtgca gtcccgcctc ctggcgctgc agaggagcgg agggttgtgc gggcagccga
5821 ggccgctgac ggagctggac gcccaccacc gcatcctgcg ctacaccaac taccaggtgg
5881 cgctggccag caagggcctg ctgccggccc gccgcgccgt gccccgggga ggcagggcgg
5941 ccttcctcag ctgcggcctg gcgcccaact cgatctgctc ctctcccgcg ggcccttctc
6001 gttcttccgc ggcggctagg agctgcccga cgcctacaag cgcagcgacc ggcgggccgc
6061 cctggccgcg cgctggcggc gcacggtgct gctgctggcg gccgtgaacc tggcgctgag
6121 cccgctggtg ctggcctggc aagtgctgca cgccctctac agccgcgctg agctgctgcg
6181 gcgcgagccc ggcgcgctgg cgacgcgccg ctggtcccgc ctggcccgcc agcagctgcg
6241 ccacttcaac gagctgccgc acgagctgcg cgcgcgcctg gcccgcgcct cccgccccgc
6301 cgccgccttc ctgcgcgccc ccgcgcccgt gctcgcgctg ctggcccgcc agctcgtctt
6361 cttcgccggc gcgctcttcg ccgcgctgct cgtgctcacc gtctactaca aggatgtgct
6421 ctacgtggag cacgtgctca ccgccatgac cgcgctcggg atagtggcca gtggccaggt
6481 gcgatggggg agatgtccgg ggaggggcat tcttctgtcc tggggctgag cctcccgcgc
6541 atccctaccc cttccaccgc tagtggcccc ccagcccggc atcctagccg cacatcctct
6601 aaacggcagg ccccttttcc atccccctcc cgccctctag gtctttcatt ccgggcgggc
6661 aggccaaggt cgttcgccg caaaccctgc ccagctgcag ccacggccag cctcctggcc
6721 tccatttccc gaatgaccca ggactcaagg tgaggggtgg gggcctgaga cgcagggagc
6781 agggctggtg gggctgatcc tccaggagaa gggtgcaggg gcatgggggc gggggtgacc
6841 agaaaggaga ggtgaagcct ggccctccct ggggcatgtg ggatggggg gacctgtctt
6901 tttttcatcc actgtgccct ctgttctagc tgtgtgtccc caggaggcag tgggggccag
6961 aagctggccc agctcccaga gcttgtgtct gctgagtctc cacaccattt acctgcacca
7021 ggtgagtaga ggggtgccag aaagagaaat gcggggagaa agcagctgaa caaaatggag
7081 agtgtggagg ggctggagtg taaggaattt acccaacagg aaagaacaca gttcagaaga
7141 gggtgagggg aactaagagg tggcccttgt tggtgacaag gcgaagggag ggcgaggggg
7201 cagaaaggga aagaaaggtg caggtttcgt gccgagggac actgggcgag gatgcgagga
7261 catgtgggtg ttcgcactcc cctcctggga tcctctcaac agccccgccc actggctagt
7321 cactgctctt tttttttttt tttttttttg cggtacgcgg gcctctcact gttgtggcct
7381 ctcccgttgc ggagcacagg ctccggatgc gcaggctcag ccgccatggc tcacgggccc

```
7441 agccgctccg cggcatgtgg gatcttcccg gaccggggca cgaacccatg tcccctgcgt
7501 cggcaggcgg actctcgacc actgcgccac cagggaagcc ccattgtcct catctgacaa
7561 atgagggccc taaggctagg ggaagtgaag tggctagctc gaggtcgcag atggcaagca
7621 gtggagctgg gggtggaacg cagctctgct ttgagtgagg caggcaaact cctaccaaac
7681 cacagccccg cacagccagg gacaggctcc aagctgaggg tgggggagga gatttcagca
7741 ccctctgcac cgagcccagg ggctgggcct caggctgcag ggccggaggg agcccttcat
7801 ccttcttctc tccatttagc tccatcggca gcagcagcag gaactgtggg gcgaggcttc
7861 agccttctcc ctgtccaggc cctgggccag cccgccgcag acactctcgc cggacgagga
7921 gaagccatcc tggtccagtg atggtgaggc ggtcagggtg tgtgggggac agaccctggg
7981 tgaactgcag gtatcatggc actgcccagc tgatggcttt tctcaccagg ctccagtcct
8041 gcttccagcc ccagacagca gtggagaacc cagaggaccc agaatctgtt ccctggaggg
8101 tttcaggagc ccacagacac ccagaaggag gctggccagg ccactagcac tgactgagag
8161 ggctgctcac ggccagtatt gtctaactgg ggtggaggca gggcaacgag taggacactg
8221 ggtggactca gactcagagg tatgggaacg gtgatgggga tggtcatctc tggaacctaa
8281 accccctggac tgcttgtcaa gtgtttttcc tcccccaggt tccctggctt cttctccagc
8341 cacaggagat cggatgaggt agagtggaag ggcacgccga gccctcttta aagaccccca
8401 tggatgcttt agctgggcgg aacttgggag tcaattagga actcgccccg gagaagagag
8461 aaagggtagg agaggagacg cccaaggcat cgggaaacat gtcttagaga cagtcctcac
8521 acggaggggc tgagagacaa ctatctgtcc tggagtggtc gcctgggaag aagtacccaa
8581 gtgacctcac gttggagggc agagccaaga tggaggccaa gctgcacata agggagccat
8641 gaggggctgg acccgaaagg cagtaggttc tttttttttt ttttgcggta cgtgggcctc
8701 tcactgttgt ggcctctccc gttgcggagc acaggctccg gacgcgcagg ctcagcggcc
8761 atggctcacg ggcgcagccg ctccgcggca cgtgggatcc tcccggaccg gggcacgaac
8821 ccgcgtcccc tgcatcggca ggcggactct caaccactgc gccaccaggg aagccccagt
8881 aggttctttg gacacatctg gactttaggt tcccatggga cttgcggaag ggcatgggga
8941 ctgggggtgg gaagactaga gaggcctgac gttttcggag ttcacgttgg cgtgacatct
9001 gaggaagcgg gtctggcccc ttgtcctgtc cgaaacaagc agtaaagttt gctcatggct
9061 gtgctgcaca gctttgcgac cttagtcctg gaatccccac tcctgtcagc tgacgactgc
9121 acctgcttct cagtccagat cagggagcaa cacagaagaa ctgcccgtga ttggctggag
9181 accaccactg ttctccagga ctccctgtca aagaattacc acctccacac tgtctgcacg
9241 ggttcaaaga caaagcttgt cattcattct gtaactgtga ttgacagcgc tgggttaggc
9301 atcttagtag ctctcataac ttctgatctg gtctcttatg gtgaatcgtc agtaataaat
9361 atttcaatga tttaga
```

Figure S2. *ATG9B* genes and proteins of tetrapods. ATG9B gene and protein information of (A) the tropical clawed frog, *Xenopus tropicalis*, (B) axolotl, *Ambystoma mexicanum*, (C) caecilian, *Rhineura floridana*, (D) gecko, *Sphaerodactylus townsendi*, (E) green anole lizard, *Anolis carolinensis*, (F) Asian vine snake, *Ahaetulla prasina*, (G) chicken, *Gallus gallus*, (H) ostrich, *Struthio camelus*, (I) echidna, *Tachyglossus aculeatus*, (J) opossum, *Monodelphis domestica*, (K) manatee, *Trichechus manatus latirostris*, (L) elephant, *Loxodonta africana*, (M) cattle, *Bos taurus*, (N) grey whale, *Eschrichtius robustus*, and (O) common bottlenose dolphin (*Tursiops truncatus*) were downloaded from GenBank (https://www.ncbi.nlm.nih.gov/gene/, last accessed on February 8, 2026). Note that information relevant for interpretation of mRNA and protein predictions from dolphin *ATG9B* pseudogene is highlighted by red fonts in panel O. For a sequence correction of *ATG9B* of the grey whale, see Figure S8.

A

```
CCCTCACCGCGGGTCTCTCCCCAGGACCAGAACCGCTACCACGAGGACATCTTTGGCCTGACGTTCCGCACGCAGGAGGT
                         D  Q  N  R  Y  H  E  D  I  F  G  L  T  F  R  T  Q  E  V
GGCCTCGCTGATCCGCTCACAGTCCTTCTCGGCGCAGGCACGGAGCCTGCTGGACCCTGCCCCCTGAGTGGCCCCTGGCT end of NOS3 cds.
 A  S  L  I  R  S  Q  S  F  S  A  Q  A  R  S  L  L  D  P  A  P  *
CCTCGGCCCATGGGGTGGGGGCAGGGCCCTTTGCCCCATTGCTGCTCCCTCCCCCCCCCCCCGAGTCCCCAATAAAGCAT
TTAGTTTTGATAATTGTAGTTTGTCATTTCCCACGATGCGCCAGTGCCTGGGGGTGGCCCCGGGTCGGGGTGGCCTCTCG
CGCCCCCATGCTCTGGGGCCCAGCCCAGCTGTGAGGGCAGGGCCCCACATTCAGACTCTGCAGCCCAGCCTACGGGCCCG
GCACCGGCCAGCGCCACCGGCTGCACCACGGCCCAGGAGAAGGAAGCCGGGCACAGGGGGGCCCAGGCTGCCGATGCTCC
GGGGGGAGAAGGCAGCTGCTTCCCCAGGGCCCTCGCCGGGGACACCGGGCAGGGTGCAGAGCTGGGGGCCACGGAGCCTC
AGCCACAGGCAGCAGCGCCCAGTCGGTCTCCAGCCCAGGGAGCGGGGCCATTGCTTGGATGGGCGGGACTCTGGTTGGCG
GCGGGGTCACTCCGGAGGAGGGGCGTGGCTCTGGCCTGGGGGCGCGGTCACTCCGGAGGAGATGCGGGGCTCAGGCACAG
GGGTCGGAGTCAGTCAGGGGAGGGGCGGGGCTCTGGCCTGGGGGCGGGGTCGCTCCGGACGAGGGGCGGGGCTCGGGCAC
AGGGGGCGGCGTCAGTCAGGGGAGGGGCGTGGCTCTGGCCTGGGGGGCGGGGTCGCTCCGGAGGAGCGGCGAGGCTCGGG
CACAGGGGGCGGGGTCAGTCATGGGGAGGGGCGTGGCTCGGGCACAGGGGGCGGGGTCAGTCAGGGGAGGGGAGGGGCTC
GGGCACGGGGGCGGGGTCGCTCCGGCGGAGGGGCGGGGCTCGGGCACAGGGGGCGGGGTCGCTCCGGAGGAGAGGCGGGG
CTCGGGCACAGGGGGCGGAGTCAGTCAGGGGGGGCGTGGCTCTGGCCTGGGGGCGCGGTCACTCCGGAGGAGCGGCGAGG
CTCGGGCATAGGGGGCGGGGTCAGTCATGGGGAGGGGCGGGGCTCTGGCCTGGGGGCGGGGTCACCCCGGAGGACGGGCG
TGGCTCGGGCACAGGGGGCGGGGTCGCTCCGCTCGCGCTCTCTCGCTGTCGCCGTGGTGACGACCCGGGCGCCCCGCTAG
TGGCGCGGTGCATTGTGGGCCGCCGGCCGGTGCCGTCATGCTGCTGCTCTGGGCCCGCTCCGCGCTGCGCCCCGCGGCCA start of ABCB8 cds.
                                      M  L  L  L  W  A  R  S  A  L  R  P  A  A
GGTACCGCGTCCCCCGGACGCCTGGGTCCCCCGGGGCGGGGTGGGCGCGGGGTGAGCCGGGGGGGGGTCCCGCTAACTC
R
```

B

```
CCCTGGCAGTGGCGGGGGTCCCTGACAGCACATCCCCACAGGACCAGAACCGCTACCACGAGGACATCTTTGGGCTGACG
                                          D  Q  N  R  Y  H  E  D  I  F  G  L  T
TTCCGCACGCAGGAGGTGGCCTCGCGCATCCGCAGCCAGTCCTTCTCCGCACGGGAGCACAAGTCCCTGGGCTTCAACGT
 F  R  T  Q  E  V  A  S  R  I  R  S  Q  S  F  S  A  R  E  H  K  S  L  G  F  N  V
CTGAGCCGCCACGGACCAGAGCCAGCCCTCAGCCCCAATAAAGCCTCTAGTTTCCATAACTGTCACAAAAGCCACCCTCC end of NOS3 cds.
  *
TGGGGACACGCCAGGCGCCGCTGGGGTACCAAGTCCGCAAACGTTTATTGCCTGGGGCTGGGCACGCACGGACCCACGGA
AGGAGTGCTGGGTGCGTTTCCCACAATGCACCTCTTCTGCCCCGTGCATTGCCCGCTTAGGGACCTGGGGGGAACGGGGT
AGGGAAGTCAGAGTGAGGGTGACCTTGGCCCCAGCTCCCAGCATGCACTGCTCCAACATGCATCCACTCCACAGGTCTCC
CCCCAACTCCCTCCCCGCCATGGTGTGGGGGCTGGCTTGGGGCCCTGCCAGTGTCCTGCCACTGCTGTCCCTGCCCTGCC
ATGCCCAGGCCCCGTGTTCAGCCTGGCCATCGCCCACAGCCAGTCCTGTCCAGCCCACGGCTGCCTGCCCCAAACCAGTG
ACCCTGACCCTGGCTTGCAGCAATACCCAGGGGACCACCTACCCCCGTCCCAGCCCTGCCTCCCGTGTCAGGTCTGGACC
CGGCCTGACACTGGCACTTCACGTGCGACCCTCTAGTCCAGCCCTGAGCACCCCTGGCCTGGCTGGACCCCACAACTGCA
GACCCCTGTGCTGGCCTGGGCAGGAAGACTGTGTGCTCACCCGGGCGAGCCAGAAGCAGCTGAGGGAGGGCTGGGAGCCA
GGGGCTGCGGCAGTGAGCACAGACCCACCCAGGGGTGCTGCTTGAGCCCCAAACTGGGCCCCCAACTCTCAGCTGCTGCC
CAGATCAGCCCTGGCCTTGGCCCCAGCTGGGGAGCAGGGAGGCAGAAGTGGGGCCACCCACACTCCAGCATGGCTGGCCC
CCCAGGGAAGTGACGAGGCCCCCGATGGGGTAACAGCAGAGAGGATGTGTTCCCCAAACAGCATGTCCCCACGGTGCCCG
GGCTGCCCAAGCCAGTGGGGAGCGGGCAGCAGCTGTGCAGACACCGATGCTGCGCTCCTGAACCCCGCGCCCACAGCAGC
ACCTCAGCACACCCGCGATAAGGGGCGGGGCCACGCAGGTGGGAGCGGAGCCACGCAGGTGGGGGCGGGGCAAGGCCGTG
CCGGCCGGCCGCGCGGGGCATTGTGGGCCGGCGGAGGACCTGGACCCGCTCCCGCCATGCTGCTGCTGCTGCCGCTGCTT start of ABCB8 cds.
                                                         M  L  L  L  L  P  L  L
CGCGCCGGGGCCGGGGCCGGGGCCGGGGTCGGGTCGGGCCGCTCCGCCCCGCGCCTGCCCGCCGCGATCGTGGCCAGGTA
 R  A  G  A  G  A  G  A  G  V  G  S  G  R  S  A  P  R  L  P  A  A  I  V  A  R
```

Figure S3. Absence of *ATG9B* between *NOS3* and *ABCB8* in the Western painted turtle and the American alligator. The nucleotide sequences from the last exon of NOS3 to the first exon of ABCB8 of the Western painted turtle (*Chrysemys picta bellii*) (A) and the American (*Alligator mississippiensis*) (B) are shown together with translations of the coding sequence (cds.). Start and stop codons are highlighted by red fonts. Other parts of the coding sequence are marked by grey shading. GenBank accession numbers: NC_088792.1, nucleotides 994191-995630 (A); NC_081828.1, nucleotides 219984861-219986301 (B).

A

ATG9B autophagy related 9B [*Tursiops truncatus* (common bottlenose dolphin)]
Gene ID: 117313525
Assembly: GCF_011762595.2
Nucleotide sequence: NC_047042.1, complement 99236401-99228201

```
CCGAGTCGGCTATGGGGTGACGTTTTGAGGGTGTGCAGAGTGCGTCCCCCCGCCCTGGGGTATCCATAGTAACGCGTCAGCTGCTGACCTCCGGGTCATT
             M  G
CTCTTATGGGCGCTCCTGGCGGACTCTAGATGTTCCGGAGTCTCCAGGGAGTGTAGACCGCTGGTTCTGGGAGACTAAGGGACAAAAACCGTTTGGAGTT
TTTTCAAAACATGTGCACGTGCTTTGCCACCCACCCCGCTCTCCTAGGCGAGTCAGAGCTCAGGGGACGGCAGCCACATATATTTGGGAGAAAAAAAACC
CTGGTGATTCTGATAAGCTGCCCCTACTCAATGGTTTAGAACCGCTGGCGCGACCCTAAATCCATCTCCCCCACAGATGCCTTCCTACCGCAGTTAGGCT
GACTGTGCCTGAAGCTGCCACCGCACAGCTCTTACACCTCTGTTTCACTCTTAGTTATTTCTTCTGTGTAATTACTTGTTTGCATAGCTGCCTCCCCTCT
GGGCTGTGACTTCTTTTTGGGTAGTGGCTCTGTCTCATTCATCCGTCCCCTCCTTCCCCTCCAAATGCCTGGCAGGGAAGAGTGTCCTTAATAATAAATT
GACGTGAATTGGGGCCGGTGGTAGGGAGGCTGTTCATCGATGAATCTGGAGTGAAACTGGGTAGGAAAACGTGTCAGGATTAGTCTGAGGTTGGGCTGGG
TGAGGAGGCTGTATGGGAGGGTCCTAAAGTGAGTATGGTGGAATATCAGGGTGAGTGAGGCTGGGGAGAGACCAGAGGAAACCCCACATATCTGATTTTG
ACAGGTGTCTCAGAGAGATCATGGGAATTTCCTAGCCCCTCTGCAGTGTGGGATTCCATTTTGGGCACCAGGAATCTTGTTTTCATAGCTCTGCCTCCTG
CTCGGGGATAAAACTATGCAAGTCCTGAGCTACCATGACAATTCCCCTCCCAGGAAGGAAGCACAGGTTCCTCCTCTAAGTTCCTCCAGTGGGCAATCTG
GGATGTACTCTGTTCCTCTGTTTCCATGTCTTGGATACTGGTCCCTACATCAGCGCTTGGGCTTAAGCCCTGGAGGCCCTGACACATGGGTGGCTGAGTC
AGCACCTCAGGAATGGACTTCAGTAGGAAACCTGATGATTCAGGGCCTCAAGAATGGCCTTGGACAAGCTACCATACCAGCTGCCTGGGCCTCCGCCTCT
TCCTTGAGGTGGCAACAAGGAACTCAACTCAGTCAGCTCATCCCTGGGGCACCCAAATTACCTGCCCTTCCAAATGCCTGACCTTCAGCTGCCCTCTGCT
GGGTCAGCCCTGATGCATCTCAGGGGGCTCCCAAAACTGGATCAGAACTTGGTAGAGGGGGTTTCAAGAGGATAAATAAGCAAACATACTGGAGCTTCCA
AGGAACCCCACTTTCCTACTGTAAAATCTCCGAGAACCACCTCTCGTCCCAAATAACTGCCTCTCCTCCCTTCACTTGCTCCCTCTCAGTCTCCCCCACC
CTGGCTGCAAAGTCAGACGGTCTCCCCTTTTCCATTCCTTAGCACTGTCCTACAAGCAAAACATGCAGCCGTTCTCGTTTCCTTTTTTTCTTTTTGTGTT
TGGTCTTCAAGTGTTTCTCCGTGGATCACTCTGTGTGTGTGTATATAGGGAGGGGAGATAAGTTGTTTTGTTTTATTCTGAGATAAGGTTTTAAGAAACT
AAAGATGTATATCTTGGCCCCATAGCCCTATAATCCTAACTAGATTCTTTCTGTCTTTGTTCTCGGCTTCTGCTCTCTGGCCTCCCGTTTTAAGTCCGCT
GGGGGTCTGAAGGTGGGACCGGGGGGATCTCCTTCCATCTGCCGCTAGTCTGCTCCTGCCTCTCCAGGTATTGCCCTGCCAGGCATGGAGGCGGGGCCCT
AAGAGGTTTGGCCGCGGGTTGCCCTGGAAGGGGTGTGGGTGGTCGGGCTGGTGACTGCGAGTTGGGACGCCTGAGGCCTGCCCCAACTGGGTCACCGGGC
TGTTGTTGTCACTGTCCTGGTTGCTGAAGTCCCCGCTCAGCAACGCAGACCTCTTCGGAAACAGCAGCAGCTCCCATCTTTCCAAGCTTTCAGGAGATGC
CGCTGGCCTGATGGTGAGGCAGATGGGCGGGGGGTGGGGACCGGGGGCAGCTGGGGCGTGGATCCGTGCCCCTCCTCCCCGTGCCACTGCCTCCTCTTTC
                         W  A  G  G  G  D  R  G  Q  L  G  R  G  S  V  P  L  L  P  V  P  L  P  P  L  S
TCCTCCTCCGGGTCGGGGACCTGGGGGAGGGAGGGTCTCCATCTTCTCTCTGGCCCCTGCACCTCACACAAAAAGCACGCCTTCCTCAGCTCCTCCTTCA
  P  P  P  G  R  G  P  G  G  G  R  V  S  I  F  S  L  A  P  A  P  H  T  K  S  T  P  S  S  A  P  P  S
GCCCCGGGGCCCCCCTACCCAGCGGTGCAGGCCCCAGGGGCTTCTCAGCTTCGCTATAGTACCCTCCCCACTCCCGCTACCCCCGCCACGCAGCCCGACC
 A  P  G  P  P  Y  P  A  V  Q  A  P  G  A  S  Q  L  R  Y  S  T  L  P  T  P  A  T  P  A  T  QX  R  P
CGCGATGATGCCCATCTCTGCTCCCCATTCCTGGGGTTCCCACCCTGCCCCTGCCCCATGCCGATGCCCCCAGGACCCTCCTGGGCTGCGGAGAGGCCCT
  A  M  M  P  I  S  A  P  H  S  W  G  S  H  P  A  P  A  P  C  R  C  P  Q  D  P  P  G  L  R  R  G  P
CTGATCCCTGAGCAGGATTCTGAGAGGCTGGAGGACTGTGACCCTGAGGGGTCCCAAGATTCAGCCCTCCATGGGGAGGGGCAGCAGCCCCTGCTTCACG
 L  I  P  E  Q  D  S  E  R  L  E  D  C  D  P  E  G  S  Q  D  S  A  L  H  G  E  G  Q  Q  P  L  L  H
TGCCTGAAGGGCTCCGAGGTGAGGGGCTGGGGAGGGCCTCAGGGCTCCAGAGTGTCCCCTGGGCTGGGCCTCTGGCAGAGGCTTGAGGGCACGGAGGCAG
V  P  E  G  L  R
GTGAGTCCCAACGCTCTCCCCTCTGTGCCCAGGCTCCTGGCACCACGTCCAGAACCTGGACAGCTTCTTCACCAAGATATCCTTTTGTGCTCTGCCTGGC
                                  G  S  W  H  H  V  Q  N  L  D  S  F  F  T  K
AGAAGGGGCACGGGAGACAGGGAGACAGGGAGGCAGTGGGCTGGGGACTTGGGGGCTGAGGAGTCGGCGTCCTCAGGAAAGGCCCATCATGCCTCAGGCT
TAATTCTTGACGCCCTGCCACATGTACAGCTACCACCAGAGGAGTGGCTTTGCCTGTATCCTGCTGGAGGATGTCTTCCAGCTGGGGTGAGATGCTTGTC
                       M  Y  S  Y  H  Q  R  S  G  F  A  C  I  L  L  E  D  V  F  Q  L  G
CCAGACTCCAAGCTCTTCCCCTTGCTTCCTCAGTAGTGCCTGCTGCTTCTGGGCCTCCAGTGCCTTCCCAGGCAACCTAGGGGCTCAGCAGGATGGGTTA
ATCCGGGCGGGCCCGGAGGCTGGGGCAGGAGCCCTCCCAGCCCGCAGGCCTCATCACTGCACTCCCTCCTGTCCCAGACGATTCATTTTCATTGTCACCT
                                                                                   R  F  I  F  I  V  T
TCACAACCTTCCTTCTTCGCTGCGTGGATTACAGCGTTCTCTTTGCCAACCAACCAAAAAACCGAACAAGACCTGGGTCGCTCCACAGCAAAGTGACCTT
F  T  T  F  L  L  R  C  V  D  Y  S  V  L  F  A  N  Q  P  K  N  R  T  R  P  G  S  L  H  S  K  V  T  L
GTCAGATGCTAACCTACCCTCATCCCAGTGTGCCCAGCGGTGAGTGACAGCTGGTGGGGGAGGTGATGGGGGAGAGCAACTGACAAAGACAAAAGCCTCC
  S  D  A  N  L  P  S  S  Q  C  A  Q  R  X  V  T
CCTCCTTTTGTTCCTTGGTTTAGATCAGCCATTCTCCAAGTTTTTAGTCTCAGGCCACCTTTGATACACTTAAAATTGATTGAGAACCCAACTCCCGAGA
CCTTTTGTTGATGAGAATCCTATCTAGATCCACCTTGTTAGAGATTAAAGCTGTCCCTAAGAACGGACAAACATACATTCCATCGACTGACCGTGATGTC
ACGTAGCCGCTGGAAAAGGCCACTGCGTACTCTTGGGAGAAGGAGAATGAAAATGGCAGAAAACCTTAATATTATATGAAAAGTTTTGACTTTGCAGACA
CTCACAAGGGTCTGGGGACCCCTAGGGCCCACACTTTGAGAACCACTGGTCTGGCTGGGTCCCCTCTCCAGGACCCCCTTGCTCCCCAAGCCAGGTCTCC
CTTGCTATGGGGCCGCTGCTTTCTCTCTTCCGTCTGTCCCGCCTGGCACAGGATCCGCTCCTGCTTGTCTTCCTGCTGATCCTGGCGGCTGCCGTCTGGC
                                                       D  P  L  L  L  V  F  L  L  I  L  A  A  A  V  W
TCTTCCAGCTGCTTTGCTCGGTCTGCAAACTCTTCAGCTACTGGGACATCCAGGTGTTTTCCAGGGAGGCCCTGCACATCCCCCCAGTGAGTTGGGTGAG
L  F  Q  L  L  C  S  V  C  K  L  F  S  Y  W  D  I  Q  V  F  S  R  E  A  L  H  I  P  P
CGTGTCTGTGGAGGCGTGCCATGTCAGGGGGCGGAGGGTGGGAAGGAGTAGGGAGCTGTACTGACAGAAGGATCCACTGGGAAGAGGGCAGAAGCCCTTA
GAGACACCTACCCTTGGCCCCGAATAATGTTAGGAGGTGGGGTACCGCCCCTTAGAACACAAGGTCCTTCCTGTTGTGGAGCTTAGTTGATGTAGATGGT
TATGGAAGATGATGCACAGTCACGTGCTTGTGGTCCAAACAATGGGTCTTTAGAGGAAGGGCAGTCACCTGACTTTCCAGAGCAGGAGGAACTGGGCTTG
GGCTGGGGAGGATAGGAGGCGGGGAGGCATTCCAAACCCCAGGCAGGAAGGGGGCAGGGGAAGCAATGCGCATCTTTGGGCAGCAGTAAGGAGACTGACC
CACCAGCCTGCAACTGAGTGTTCAGGCCCCCGTCAGTGAGCCCACCCCCCTTTCTGGCCCCTGTTTGCAGGAAGCTACTTCGTCGGCCCCTTCACTGTCC
CTGGTCACGCTTTGCTCTTTCCTCCCCATCTCGCTCATGCTCCTTCTGGCTCTGTACCCACGTTCTTTCTTTCTTTCTTTTTTTTTTTTTCGCGGTACGC
GGGCCTCTCACTGTTGTGGCCTCTCCCGTTGTGGAGCACAGACTCCGGACGCGCAGGCTCAGTGGCCATGGCTCATGGGCCCAGCCACTCCACGGCATGT
GGGATCTTCCCGGACCGGGGCACGAACCCGCGTCCCCTGCATCGGCAGGCGGACTCCCAACCACTGCGCCACCAGGGAAGCCCCCCAAGTTCTTTCTTTA
AGGCACGGCGCAAAGGCTACTTTCAGCAGCCAGCTTTCTCTGCTCCTCTCAGAAGTGCAGAGACTTTTACACAGCCCTCCTGTGCAGCAAAGGGGAGAGG
TTCTCGAAAGCCCACCTGAGTATGGAAGCACAAGCCATAGCACAGGAAAGAGTCGCGAGGGCTGGGGATGGAGGCAGGGAAGCTGAGGAGCTGGACCACT
GCTCCTCCAGGAGGTGCCTGACCCCTCTTTCTCCTTCCTTCTTTCCCCTCTTCCTTCCAGGGGAAAGGGAGACCCTGGGATGGACTGCAGAGGCTGGCCT
GGAGGGACTTGACTAAGCTGGACTGGGGAGGCTGGGTGAAACTGGGCTAAAATGAGGTTTGCAGATGAGCTCCAAGTGAGATCTGACAGCCTCCACCCCG
ACGGGGACAAGGCCACTGTCTTAGGAAGGTTAATCTGTTCGAGTGGGATGGGGCGGGGTGTGTGTGTGTGTGTCTCAAAGCATGCTGAGGTAAAAGTTTG
GCGGCTTCCGGGCCCAGCGGGCTGGTACCCCAGGAACGGGAAAGAGACTGCCTTGTTACCTGCAGCTCTGATGTACCTTGTATGTGTAGGGCTGCGGGCA
CGGGTGGGCGAGCGGTGGCCAGGAAAACCTGCTCTTACGGAGCTGAGCTCAGATACATGTAGAGTTGGTTTATGAGCTCTTCCTGGGAAGCACATGACTC
ACGGCCTCAGGAGGCGCAGGGGCCCGAGGAACACAGTTCAGAAGAGGGTGAGGGGAACTAAGAGGTGCCCCTTGTTGGTGGCAAGGCGAAGGGAGGGCGA
GGGGGCAGAAAGCGAAAGGTGGCAGCTTTCGTGCCCGAGAGACACTGGGTGAGGACGGCGGGGATACATCTCTGGTCACGGGGAGTTTGGGCATCGCCGT
GGAGAGGAATCCAGGTGGCTGGCGCTCAGGCGTCCCCTCTCCGCAGGAGGAGCTCAGCTCGGTGCCCTGGGCCGAGGTGCAGTCCCGCCTCCTGGCGCTG
                                                 E  E  L  S  S  V  P  W  A  E  V  Q  S  R  L  L  A  L
CAGAGGAGCGGAGGGTTGTGCGGGCAGCCGAGGCCGCTGACGGAGCTGGACGCCCACCACCGCATCCTGCGCTACACCAACTACCAGGTGGCGCTGGCCA
 Q  R  S  G  G  L  C  G  Q  P  R  P  L  T  E  L  D  A  H  H  R  I  L  R  Y  T  N  Y  Q  V  A  L  A
GCAAGGGCCTGCTGCCGGCCCGCCGCGCCGTGCCCCGGGGAGGCAGGGCGGCCTTCCTCAGCTGCGGCCTGGCGCCCAACTCGATCTGCTCCTCTCCCGC
S  K  G  L  L  P  A  R  R  A  V  P  R  G  G  R  A  A  F  L  S  C  G  L  A  P  NX  D  L  L  L  S  R
GGGCCCTTCTCGTTCTTCCGCGGCGGCTAGGAGCTGCCCGACGCCTACAAGCGCAGCGACCGGCGGGCCGCCCTGGCCGCGCGCTGGCGGCGCACGGTGC
 G  P  F  S  F  F  R  G  G  X  E  L  P  D  A  Y  K  R  S  D  R  R  A  A  L  A  A  R  W  R  R  T  V
TGCTGCTGGCGGCCGTGAACCTGGCGCTGAGCCCGCTGGTGCTGGCCTGGCAAGTGCTGCACGCCCTCTACAGCCGCGCTGAGCTGCTGCGGCGCGAGCC
L  L  L  A  A  V  N  L  A  L  S  P  L  V  L  A  W  Q  V  L  H  A  L  Y  S  R  A  E  L  L  R  R  E  P
```

```
CGGCGCGCTGGCGACGCGCCGCTGGTCCCGCCTGGCCCGCCAGCAGCTGCGCCACTTCAACGAGCTGCCGCACGAGCTGCGCGCGCGCCTGGCCCGCGCC
  G  A  L  A  T  R  R  W  S  R  L  A  R  Q  Q  L  R  H  F  N  E  L  P  H  E  L  R  A  R  L  A  R  A
TCCCGCCCCGCCGCCGCCTTCCTGCGCGCCCCCGCGCCCGTGCTCGCGCTGCTGGCCCGCCAGCTCGTCTTCTTCGCCGGCGCGCTCTTCGCCGCGCTGC
 S  R  P  A  A  A  F  L  R  A  P  A  P  V  L  A  L  L  A  R  Q  L  V  F  F  A  G  A  L  F  A  A  L
TCGTGCTCACCGTCTACTACAAGGATGTGCTCTACGTGGAGCACGTGCTCACCGCCATGACCGCGCTCGGGATAGTGGCCAGTGGCCAGGTGCGATGGGG
L  V  L  T  V  Y  Y  K  D  V  L  Y  V  E  H  V  L  T  A  M  T  A  L  G  I  V  A  S  G  Q  V  R  W  G
GAGATGTCCGGGGAGGGGCATTCTTCTGTCCTGGGGCTGAGCCTCCCGCGCATCCCTACCCCTTCCACCGCTAGTGGCCCCCCAGCCCGGCATCCTAGCC
  R  C  P  G  R  G  I  L  L  S  W
GCACATCCTCTAAACGGCAGGCCCCTTTTCCATCCCCCTCCCGCCCTCTAGGTCTTTCATTCCGGGCGGGCAGGGCCAAGGTCGTTCGCCGCAAACCCTG
                                                   G  L  S  F  R  A  G  R  A  K  V  V  R  R  K  P  C
CCCAGCTGCAGCCACGGCCAGCCTCCTGGCCTCCATTTCCCGAATGACCCAGGACTCAAGGTGAGGGGTGGGGGCCTGAGACGCAGGGAGCAGGGCTGGT
  P  A  A  A  T  A  S  L  L  A  S  I  S  R  M  T  Q  D  S  S
GGGGCTGATCCTCCAGGAGAAGGGTGCAGGGGCATGGGGGCGGGGGTGACCAGAAAGGAGAGGTGAAGCCTGGCCCTCCCTGGGGCATGTGGGGATGGGG
GGACCTGTCTTTTTTTCATCCACTGTGCCCTCTGTTCTAGCTGTGTGTCCCCAGGAGGCAGTGGGGGCCAGAAGCTGGCCCAGCTCCCAGAGCTTGTGTC
                                          C  V  S  P  G  G  S  G  G  Q  K  L  A  Q  L  P  E  L  V  S
TGCTGAGTCTCCACACCATTTACCTGCACCAGGTGAGTAGAGGGGTGCCAGAAAGAGAAATGCGGGGAGAAAGCAGCTGAACAAAATGGAGAGTGTGGAG
  A  E   L  H  T  I  Y  L  H  Q
GGGCTGGAGTGTAAGGAATTTACCCAACAGGAAAGAACACAGTTCAGAAGAGGGTGAGGGGAACTAAGAGGTGGCCCTTGTTGGTGACAAGGCGAAGGGA
GGGCGAGGGGGCAGAAAGGGAAAGAAAGGTGCAGGTTTCGTGCCGAGGGACACTGGGCGAGGATGCGAGGACATGTGGGTGTTCGCACTCCCCTCCTGGG
ATCCTCTCAACAGCCCCGCCCACTGGCTAGTCACTGCTCTTTTTTTTTTTTTTTTTTTTTGCGGTACGCGGGCCTCTCACTGTTGTGGCCTCTCCCGTTG
CGGAGCACAGGCTCCGGATGCGCAGGCTCAGCCGCCATGGCTCACGGGCCCAGCCGCTCCGCGGCATGTGGGATCTTCCCGGACCGGGGCACGAACCCAT
GTCCCCTGCGTCGGCAGGCGGACTCTCGACCACTGCGCCACCAGGGAAGCCCCATTGTCCTCATCTGACAAATGAGGGCCCTAAGGCTAGGGGAAGTGAA
GTGGCTAGCTCGAGGTCGCAGATGGCAAGCAGTGGAGCTGGGGGTGGAACGCAGCTCTGCTTTGAGTGAGGCAGGCAAACTCCTACCAAACCACAGCCCC
GCACAGCCAGGGACAGGCTCCAAGCTGAGGGTGGGGGAGGAGATTTCAGCACCCTCTGCACCGAGCCCAGGGGCTGGGCCTCAGGCTGCAGGGCCGGAGG
GAGCCCTTCATCCTTCTTCTCTCCATTTAGCTCCATCGGCAGCAGCAGCAGGAACTGTGGGGCGAGGCTTCAGCCTTCTCCCTGTCCAGGCCCTGGGCCA
                               L  H  R  Q  Q  Q  Q  E  L  W  G  E  A  S  A  F  S  L  S  R  P  W  A
GCCCGCCGCAGACACTCTCGCCGGACGAGGAGAAGCCATCCTGGTCCAGTGATGGTGAGGCGGTCAGGGTGTGTGGGGGACAGACCCTGGGTGAACTGCA
S  P  P  Q  T  L  S  P  D  E  E  K  P  S  W  S  S  D
GGTATCATGGCACTGCCCAGCTGATGGCTTTTCTCACCAGGCTCCAGTCCTGCTTCCAGCCCCAGACAGCAGTGGAGAACCCAGAGGACCCAGAATCTGT
                                        G  S  S  P  A  S  S  P  R  Q  Q  W  R  T  Q  R  T  Q  N  L
TCCCTGGAGGGTTTCAGGAGCCCACAGACACCCAGAAGGAGGCTGGCCAGGCCACTAGCACTGACTGAGAGGGCTGCTCACGGCCAGTATTGTCTAACTG
F  P  G  G  F  Q  E  P  T  D  T  Q  K  E  A  G  Q  A  T  S  T  D  *
```

B

ATG9B autophagy related 9B [*Kogia breviceps* (pygmy sperm whale)]
Gene ID: 131762933, updated on 6-Sep-2025
Assembly: GCF_026419965.1
Nucleotide sequence: NC_081318.1, 6397471-6407870

```
TGTATGGGAGGGTCCTAAAGTGAGTATGGTGCCATATCAGGGTGAGTGAGGCTGGGGAGAGACCAGAGGAAACGCCATATATTTGATTTTGACAGGTGTC
                          M  V  P  Y  Q
TCAGAGAGATCATGGGAATTTCCTAGCCCCTCTGCAGTGTGGGATTCCATTTTGGGCACCAGGAGTCTTGTTTTCATAGCTCTGCCTCCTGCTCGGGAAT
AACGCTATGCAAGTCCTGAGCTACCATGACAATTCCCCTCCCAGGAAGGAAGCACAGGTTCCTCCTCTAAGTTCCTCCAGTGGGCAGTCTGGGGTATACT
CTGTTCCTCTGTCTCCATGTCTTGGACACTGGTCCCTACATCAGCGGTTGGGCTTAAGCCCTGGAGGCCCTGACACGTGGGTGGCTGAGTCAGCACCTCA
GAAATGGACTTCAGTAGGAAACCTGATGATTCAGGGCCTCAAGAATGGCATTGGACAAGCCACCATACCAGCTGCCTGGGCCTCCGCCTCCTCCTTGAGG
TGGCAACAAGGAACTCAACTCAGTCAGCTCATCCCTGGGGCACCCAAATTACCTGCCCTTCCAAATTCCAGGCCTTCAGCTGCCCTCTGCTGGGTCAGCC
CTGATGCTTCTCAGGGGGCCCCCAAAACTGGATCAGAGCTTGGTAGAGAGGGTTTCAAGAGGATAAAAAAGCAAACATACTGGAGCTTCCGAGGAACCCC
ACTTTCCTACTGTAAAAGCTCCAAGAACCACCTCTCATCCAAAATAATTGCCTCTCCTCTCTTCACTTGCTCCCTCTCCTCAGTTTCCCCCACCCTGGCT
GCAAAGTCAGATGGCCTCCCCTTTTCCAGGCCTTAGCACTGTCATACAAGCAAAACATGCAGCCTTTTTCATTTTCTTTTTTTCTTTTCGTGTTTGGTCT
TAAAGTGTTTCTTGGGCTTCCCTGGTGGCACAGTGGTTGGGAGTCCGCCTGCCGATGCACGGGACATGGGTTCGTGCCCCGGTCCGGGAGGATCCCACGT
GCCGTGAAACGGCTGGGCCCGTGAGCCATGGCCGCTGAGCCTGCGCGTCCGGAGCCTGTGCTCCGCAACGGGAGAGGCCACAACAGTGAGAGGCCCGTGT
ACAGCAGGAAAATTTTTTAAAAAGCGTTTCTCTGTTGATCACTCTGTGTGTGTGTATATAGGGAGGGGAGGTAAGTTGTTTTATTTTTTTCTGAGATAAA
GTTTTAAGAAACTAAAGATGTGTATCTTGGCCCCATAGCCCTAGAATCCTTACTAGGATTCTTTCTGTCTTTGTGCTTGGCTGCTGCTCTCTGCCCCTCC
CCCACTTTAAGTCCGCTGGGGGTCTGAAGGTGGGGCCGTGGGGATCTCCTTTCATCTGCTCCTCGTCTGCCCCTGCCTCGCCAGGTACTGCCCTGCCAGG
AGTGGAGGTGGGGCCCTAAGAGGTTTGGCCGAAGGTTGCCCTGGAAGGGGTGTGAGTGGTCGGGCTAGTGACTGCGAGTTGGGACGCCTGAGGCCCCAAC
TGAGTCACTGGGCTCTTGTTGTCACCTGGTTGCTGAAGTCCCCGCTCAGCAATGCAGACCTCTTCGGAAACAGCAGCAGCTCCCATGCTTCCAAGCTTTC
                                                                                               A  F
AGGAGATGCCGCTGGCCTGATGGTGAGGCAAATGGGCGGGCGGAGGGGGGGGCGGGGACCAGGGGGCAGCTGGGGCGGTGGGGAGACCTGGGGCGTGGAT
 R  R  C  R  W  P  D  G  E  A  N  G  R  A  E  G  G  A  G  T  R  G  Q  L  G  R  W  G  D  L  G  R  G
CCGTGCCCCTCCTCCCTGTGCCACCGCCTCCTCTTCCTCCTCCTCCGGGTCGGGGACCTGGGGGAGGGAGGGTCTCCATCTTCTCTCTGGCCCCTGCCCC
S  V  P  L  L  P  V  P  P  P  P  L  P  P  P  P  G  R  G  P  G  G  G  R  V  S  I  F  S  L  A  P  A  P
TCACACAAAAAGCACCCCTTCCTCAGCTCCTTCCTCAGCCCCGGGGCCCCCCTACCCAGCAGTGCAGGCCCCAGGGGCTCCTCAGCTTCGCTATAGTACC
  H  T  K  S  T  P  S  S  A  P  S  S  A  P  G  P  P  Y  P  A  V  Q  A  P  G  A  P  Q  L  R  Y  S  T
CTTCCCAGTCCTGCTACCCCCACCACGCAGCCCCGGCCCGCGATGACGCCCCCTTCCTGGGGTTCCCTCCCCTGGCCTCGGGGACTGCCCCTCTCCCACG
 L  P  S  P  A  T  P  T  T  Q  P  R  P  A  M  T  P  P  S  W  G X  P  P  L  A  S  G  T  A  P  L  P  R
CCGACGCCCCCAGGACCCTCCCGGGCTGCGGAGAGGCCCTCTGATCCCTGAGCGGGGTTATGAGTGGCCGGAGGACCGTGACCCTGAGGGGCCCCGAGGT
  R  R  P  Q  D  P  P  G  L  R  R  G  P  L  I  P  E  R  G  Y  E  W  P  E  D  R  D  P  E  G  P  R  G
TCAGCCCTCCATGGCGAGAAGCAGCACCCCCTGCTTCATGTGCCTGCAGGGCTCCGAGGTGAGCGGGCGGGTGTGGCGCTGCGGAGGGCCTCAGGGCTGC
 S  A  L  H  G  E  K  Q  H  P  L  L  H  V  P  A  G  L  R
AGAGTGTCCCCCCGGGCTGGGCCTCTGGCAGAGGCTTGAGGGCACCGCGTCAGGTGAGACCCAACACTCTCCCCTCTGTGCCCAGGCTCCTGGCACCACG
                                                                                     G  S  W  H  H
TCCAGAACCTGGACAGCTTCTTCACCAAGATATCCTTTTTTTTTTTTTTTTTTTTTTTTTTTGCTGTACACGGGCCTCTCCCTGCCGTGGCCTCTCCCGTT
V  Q  N  L  D  S  F  F  T  K
GCGGAGCACAGGCTCCAGACGCGCATGCGCAGCGGCCACGGCTCACGGGCCCAGCCGCTCCGCGGCACGTGGGATCTTCCCGGACCGGGGCACGAACCCG
CGTCCCCTGCATCGGCAGGCGGATTCTCAACCACTGCGCCACCAGGGAAGCCCGTCCAAAATATCCTTTTGTGCTCTGCCTGGCAGAAGGGGCACGGGAG
GCAGGGAGACAGGGGTGCAGTGGGTCGCGGACTTGGGGGCTAAGGAGTCGGCGTCCTCAGGAAAGTCCCATCATGCTGCAGGCTTAATTCTTGACGCCCT
GCCACATCTAGAGCTACCACCAGGGGAGTGGCTTTGCCTGTATCCTGCTGGAGGATGTCTTCCAGCTGGGCTGAGAGGCTTGTCCCGGACCCCAAGCTCT
      I  X  S  Y  H  Q  G  S  G  F  A  C  I  L  L  E  D  V  F  Q  L
TCCCCTTGCTTCCTCAGTAGTGCATGCTGCTTCTAGCCCTCCAGCGCCTTCCCAGGCACCCCAGGGGCTCAGCAGGATGGGTGAATCCAGGCGGGCCCGG
AGGCTGGGGCAGGAGCCCTGCCAGCCCTCTGGCCTGATCACTACTGCACTCCCTCCTGTCCTAGACGATTCATTTTCATCGTCACCTTCACAACCTTCCT
                                                               G  R  F  I  F  I  V  T  F  T  T  F  L
TCTTCGCCGTGTGGATTACAGCGTTCTCTTTGCCAACCAACCAAAAAAACCGAACAAGACCTGGCTCGCTCCACAGCAAAGTGACCTTGTCAGATGCTAT
  L  R  R  V  D  Y  S  V  L  F  A  N  Q  P - K  N  R  T  R  P  G  S  L  H  S  K  V  T  L  S  D  A  I
CCTACCCTCATCCCAGTGTGCCCAGCGGTGAGTGACAGCTGGTGGGGGAGGTGATGGGGGAGATCAACCGACAAAAGCCTCCCCTCCTTTTGTTCCTTGG
  L  P  S  S  Q  C  A  Q  R
TCTAGATCAGCCATTCTCAGGCCACCTTTAATACATTTAAAATTGGTTGGGAACCCAACTCCCAAGAACTTTTGTTTATGAGAATCCTATCTATATTTAC
```

```
CTTGTTAGAGATTAAAGCTGGGAACAGCTTTAAAACCTAAGAACGGGGCTTCCCTGCTTCTCAACGCAGTGGTTGAGAGTCCGCCTGCCGATGCAGGGGA
TGCGGGTTCGTGCCCCGGTCACGGAGGATCCCACGTGCCGCGGAGCGGCTGGGCCCGTGAGCCATGGCCGCTGAGCCTGCGCGTCCGGAGCCTGTGCTCT
GCGACAGGAGAGGCCACAACAGTGAGAGACCCGCGTACCGCATTAAAAAAAAAAAAAAAAAAAAAAAAAAAAAAAAAAAAAAACAGAAAAAAATCCTAAGA
ACGTACAAACATACATTCCATCGACTGTGACCATGATGTCACGTAGCCTCCGGAAAAGGCCACTGTACACTCTTGGGAGAAGGGGAATGAAAATGGCATA
AAACCTTAATATTCTATGAAAAGTTTTGACTTTGCAGACACTCACAAGGGTCTGGGGACCCCTCGGGCCCACACTTTGAGGACCACTGGTCTGGCTGGGT
CCCCTCTCCAGGACCCCCGTCGCTCCTCAAGCCCGGTCTCCCTTGCTGTGTATGGGGCCCCTACTTTCTCTCTTCTGTCTGTCCCAGCGGCACAGGATGC
                                                                                                  M
GCTGCAGCCCCCTGCTGGTCTTCCTGCTAATCCTGGCTGCTGCCTTCTGGCTGTTCCGGCTGCTTCGCTCGGTCTGCAAACTCTTCAGCCACTGGGACAT
R  C  S  P  L  L  V  F  L  L  I  L  A  A  A  F  W  L  F  R  L  L  R  S  V  C  K  L  F  S  H  W  D  M
GCAGGTGTTTTCCAGGGAGGCCCTGCACATCCCCCCAGTGAGTTGGGTGAGCGTGTCTGTAGAGGGGTGCCATGTCAGGGGGCAGAGGATGGGAAGGAGT
  Q  V  F  S  R  E  A  L  H  I  P  P
AGGGGGCTGTACGGACAGAAGGATCCGCTGGGAAGAGGGCAGAAGGCCTTAGTGACACCTACCCTTGGCCCCGAATAATGTTAGGAAGTGGGGTACCACC
CCTTAGAACACAAGGTCCTTACCCTTGTGGAGCTTAGTTGATGCAGATGGTTATGGAAGATGATACACAGTCACATGCTTGTGGTCCAAACAATGGGTCT
TTAGAGGAAGGGCCGTCACCTGACCCTACAGAGCAGGACCAACCGGGGTTGGGCTGGGAAGGATAGGAGGTGGGCAGGCATTCCAAACCCCGGGCAGGAA
GGGGGCAGGGGAAGCAATGAGCATCTTTGGGCAGCAGTAAGGAGACTGACCCACCAGCCTGCAACTGAGTGTTCAGGCCCCGGTCGGTGAGCCCACCCCC
TTTTCTGGCCCGTTTGCAGGAAGCTACTTCGCCGGCCCCTTCACTGTCCCTGGTCAGCTTTGCTCTTTCCTCCCCATCTTGCTCATGCTTCTTCTGGCTC
TGTACCCAAGTTCCTTTTTTTTTTTTTGCTGTACACGGGCCTCTCACTGTTGTGGCTTCTCCCGTTGCAGAGCACCAGCTCCAGACGCTCAGGCTCAGTG
GCCATGGCTCACGGGCCCAGCCGCTCCGCGGCACGTGGGATCTTCCCAGACCGGGGCACGAACCCGCGTCCCCTGCATCGGCAGGCGGACTCTCAACCAC
TGCGCCACCAGGGAAGCCCCTTTTTAAAAAATGTTTTTTATTTTTATTATATTTTAAATGCATTTATTTCATTTATTTATATTTGGCTGTGTTGGGTCTT
TGTTGTTGTGCATGGGCTTTCTCTAGTTGCTGCGAGCGGGGGCTACTGGGCTACTCTTCGTTGTGGTGTGCGGGCTTCTCATTGCGGTGGCTTCTCTTTG
TCGCACAGCACTGGGCTCTAGGCACGTGGGCTTCAGTAGTTGTGGCACGCGGGCTCAGTAGTTGTGGCACACAGGCTTCGTTGCTCCCCGGCATGTGGGA
TCTTCCCGGACCGGGGCTCGAACCCGTGTCCCCTGCATTGGCAGGTGGATTCTTAACCACTGCGCCACCAGGGAAGCCCCCAAGTTCTTTCTTTAAGGCA
CAGTGCAAAGGCTACCTTCAGCAGCCAGCTTTCCTTGCTCATCTCAGAAGTGCAAAGCCTTTTACACAGCCCTCCTGTGCAGCAAAGGGGAGAGGTTCTC
GAAAGCCCACCTAAGCACGGAAGCACAAGCCAGAGCACAGGAAAGAGTGGCGAGGGCTGGGGATGGGGGTGGGGAAGGTCAGGAGCTGGACCACTGCTCC
TCCAGGAGGTGCCTGCCCCATCTCTTTCTCCTTCCTTCTTCAACCTCTTCCTTCCAGGGGAAAGGGAGACCCTGGGATGGACTGGGGAGGCTGGCCTGGA
GGGACTTGACTAAGCTGGACTGGGGAGGCTGGGTGAAACTGGGTTAAATGAAGTTTGCAGATGAGCTCCAAGTGAGATCTGACAGCCTCCACCCCAGATG
GGGACATGGCCATTGTCTTAGGAAGGTTAATCTGAGGGGGTGGGGTGGGGCGTGTGTGTGTGTGTGTGTGTGTGTGTGTGTGTGTGTGTGTGTATCTCAA
AGCGTGCTGAGGTAAAAGTTTGGGGGCTTCTGGGCTCAGCGAGCTGGTACCCTAGGAACGGGAAAGAGACTGCCTTGTTACCTGCAGCTCTGATGTACCT
TGTATGTGTAGGGCTGTGGGCATGGGGGGGCGAGCGGTGGCCAGGAAAACCTGCTCTTAAGGAGCTGAGCTCAGATACATGTAGAGTTCGGTTTATGAGC
TGTTCCTGGGAAGCACATGACTCACGGCCTCAGGAGGGACAGGAGGCTGAGGAACACAGTTTAGAAGAGGATGAGGGGAACTAAGAGGTGCCCCTTGTTG
GTGGCAAGAAGGGAGGGCGAGGGGGCAGAGAGCGAAAGGTGGCAGCTCTCGTGCCCGAGAGACACGGGGGGAGGGCGGCAGGGATGCATCTCTGGTCACG
GGGAGTTGGGGCATCGCCGTGGAGAGGAATCCAGGCGGCTGGCGCTCAGGCGTCCCCTCCGCAGGACGAGCTCAGCTCGGTGCCCCGGGCAGAGGTGCAG
                                                                      D  E  L  S  S  V  P  R  A  E  V  Q
TCCCGCCTCCTGGCGCTGCAGAGGAGCGGGGCTGGGGGGGCTGGGGGCGGGGAGCTGTGCGTGCAGCCGAGGCCGCTGACGGAGCGGGACGTCCACCACC
 S  R  L  L  A  L  Q  R  S  G  A  G  G  A  G  G  G  E  L  C  V  Q  P  R  P  L  T  E  R  D  V  H  H
GCATCCTGCGCTACACCAACTACCAGGTGGCGCTGGCCAACAAGGGCCTGCTGCCGGCCCCCTGCGCCGTGCCCTGGGGAGGCAGGGCGGCCTTCCTCAG
R  I  L  R  Y  T  N  Y  Q  V  A  L  A  N  K  G  L  L  P  A  P  C  A  V  P  W  G  G  R  A  A  F  L  S
CCGCGGCCTGGCGCTCAACGTCGATCTGCTCCTCTTCCGCGGGCCCTTCTCGTTCTTCCGCGGCGGCTGGGAGCTGCCCGACGCCTACAAGCGCAGCGAC
  R  G  L  A  L  N  V  D  L  L  L  F  R  G  P  F  S  F  F  R  G  G  W  E  L  P  D  A  Y  K  R  S  D
CGGCGGGCCGCCCTGGCCGCGCGCTGGCGGCGCACGGTGCTGCTGCTGGCGGCCGTGGACCTGGGGCTGAGCCCGCTGGTGCTGGCCTGGCAGGTGCCGC
 R  R  A  A  L  A  A  R  W  R  R  T  V  L  L  L  A  A  V  D  L  G  L  S  P  L  V  L  A  W  Q  V  P
ACGCCTTCTACAGCCGCGCGGAGCTGCTGCGGCGCGAGCCCGGCGCGCTGGGGACGCGCCGCTGGTCCCGCCTGCAGCTGCGCCACTTCAACGAGCTGCC
H  A  F  Y  S  R  A  E  L  L  R  R  E  P  G  A  L  G  T  R  R  W  S  R  L  Q  L  R  H  F  N  E  L  P
GCACGAGCCGCGCGCGCGCCTGGCCCGCGCCTACTGCACCGCCGCCGCCTTCCTGCGCGCCGCCGCGGCCCCGCCCCTGCTCGCGCTGCTGGCCCACCCG
  H  E  P  R  A  R  L  A  R  A  Y  C  T  A  A  A  F  L  R  A  A  A  A  P  P  L  L  A  L  L  A  H  P
CTCGTCGTCCTCGCCCGCGCGCTCTTCGCCGCGCTGCTCGTGCTCACCGTCTACGACGAGGACGTGCTCTCCGCGGAGCACGTGCTCACCGCCATGACCG
 L  V  V  L  A  R  A  L  F  A  A  L  L  V  L  T  V  Y  D  E  D  V  L  S  A  E  H  V  L  T  A  M  T
CGCTCGGGGTCGTGGCCACCGTGGCCAGGTGCGATGGGGGAGATGTCCGGGGAGGGGGCATTCCTCTGTCCTGGGGCTGGGCCTCCAGCGCATCCCTCCC
A  L  G  V  V  A  T  V  A  R
CCTCCCCCCAGCCCTGCATCGCAGCCGCACATCCTCTGAACGGCAGGCCCCTTCTCCACCTCCGTCCCGCCCTCTAGGTCTTTCATTCCGGGTGAACAGG
                                                                                  S  F  I  P  G  E  Q
GCCAAGGTCATCAGCCGCAAATCCTGCTGCCGGCGGCCTTGGCGCTCATGCACTACCTGCCCTGCCGGCAGGACCAGCGCTTGCCGGCAGATGGCGCGGC
G  Q  G  H  Q  P  Q  I  L  L  P  A  A  L  A  L  M  H  Y  L   P  A  G  R  T  S  A  C  R  Q  M  A  R
TGCTGCAATACCGAGCGGTGAGGGTCACGCCCTTAGCGGGGCTGCGGAGGACGCTTGGCTGCCCGCGGTGTCTGGGGACAAACGGGGCTCAGGGCTGGCC
L  L  Q  Y  R  A
CTGCCGGGCGTCGGGGGAATGACTGGGTTGGAGGTTTCCTGCTTGGCGAGTTTGGTTGCCCGCACCAACCCCTTCTCAGCCTCACCCCTGCTTCCCCAGG

TCTCCTTCACGGAGGAGCTCCCGTCCCCTCTCCTCACCCCACTGTTTCTGCTCTTCTGGTTTCGCCCCCGCGCCTTGGAGCTTACTGACTTTTTCCGTCA
V  S  F  T  E  E  L  P  S  P  L  L  T  P  L  F  L  L  F  W  F  R  P  R  A  L  E  L  T  D  F  F  R  H
CTTCACTGTAGATGTGGCCGGGGTTGGGGGACATCTGTTCCTTTGCCCTTATGGATGTGAAGCGCCATGGCCACGCTCAGGTAAGAGCCTGTCCCTGGTT
  F  T  V  D  V  A  G  V -G  D  I  C  S  F  A  L  M  D  V  K  R  H  G  H  A  Q
GTGCGGGGCTGTGGTTTGGTAGGAGTTTGCTTGCCTCACTCAAAGCAGAGCTGGGTTCCACCCCCAGCTTCATTGCCTGCCACCTGTGACCTCGAGCTCG
TCACTTCACTTCCCCTCAGGGCCCTCATTTGTCAAGTGAGGACGATGACCAGCCGGTGCGTGGGGCTGTTATGAGGATTACGTGAGGTGACATGTGTAGA
GCCCTACCAGAGTCCCCAGCATGTCATAACACCTGTAAATGGGGACGCAGACCATCAGAGAGACCGAGAAACGTCCCGGTCAGTGGACAGGGCTGGAGTC
AGGCCAGCCCTGGTATCCAGTGTGAACAGGTTTGGCAAAACACTGAGGTCACGCCGTATCCTAGTGCTGAGTCTGGACCCAGGTCCAGTTCTGCGGCTGT
GTTTGTGGGGGTCTTGAGCTAGTCCCCATTCTTTCCAGTTCACCTGGGAAGAAGGAATGGGGGGGCTAGTGTTTCCTGTCCTGCCTACCCCAGGTGGCAC
CCACTACAGCCGTAAACGTGGTGTTCACGGGAGTTCCCCGGTGTCCTAGTGGTTAGGACTCGGCGCTTTCACTGCCAGGGCCCGGGTTCCATCCCTGGTA
GGGGAACTGGGATCCCGAAAGCCACGTGGCACGGCCAAAAAAAAAAAAAAAGTGCTGTTGGCAACACCTGAAGGACTGTTGTTGTCCTCAGTGGCTCTCAG
                                                                                             W  L  S
AGGGACAGACAGAGGCCTCACTGTCTGAGCGGGTGAGGACGGGAAGACTGAGCTCTCATTGATGAGGTTCTCCCTGGTTCATCCACAACGGTGCCCTCCA
E  G  Q  T  E  A  S  L  S  E  RX  E  D  G  K  T  E  L  S  L  M  R  F  S  L  V  H  P  Q  R  C  P  P
GGCCACAGCTCCAAGTTCCTGGCGCACCTTCGAGGCCGGGTGCAACAAGATGCGGCCGCCTGGGGCGCCAGCTCGGTTTGCAGCCCCCGCAGCCCTGGGA
 G  H  S  S  K  F  L  A  H  L  R  G  R  V  Q  Q  D  A  A  A  W  G  A  S  S  V  C  S  P  R  S  P  G
TCCACAGCAACTCTGCCTCCCCTCTGGGGAGTCGAAGCCTGCCTGCCCTGCTGCCCTCACCCCCCGGCCTCTCCCCACCTCTGTCTCACCTCTTGTATTT
I  H  S  N  S  A  S  P  L
ACCCCCTCACTAGCGCAGCCTGCTCTCCTGCCTTCCTTAGACCTCCGCCTCCCCTCCTGTCCTCTCTTCTGACCCTCTCTTCTCTTGCGCCCAGCCGGTG
                                                                                                P  V
GCCTTCCCGGACAGCCTCTCGATGCAGCCCCTCGTGCCCCCCCAGGACCTGAGCCCCATAGCCCCCTGCCCAGCTGCAGCCACAGCCAGCCTCCTGGCCT
 A  F  P  D  S  L  S  M  Q  P  L  V  P  P  Q  D  L  S  P  I  A  P  C  P  A  A  A  T  A  S  L  L  A
TCATTTCCCGAATGACCCAGGACTCAAGGTGAGGGGTGGGGGCCTGAGACGCAGAGAGCAGGGCTGGTAGGGCTGATCCTCTAGGAGAAGGACGCAGGGG
F  I  S  R  M  T  Q  D  S  S
CATGGGTGGGGGTGATCAGGAAGGGGAGGTGAAGCCTGGCCATGTCTTTTTTTAATCCACTGTGCCCTGTGTTCTAGCTGTGTGTCCCCAGGAGGCACTG
                                                                                  C  V  S  P  G  G  T
GGGGCCAGGAGCTGGCCCAGCTCCCAGAGCTTGTGTCTGCTGAGATGAGTCTCCATGCCATTTACCTGCACCAGGTGAGTAGAGGGGTGCCAGAAAGAGA
G  G  Q  E  L  A  Q  L  P  E  L  V  S  A  E  M  S  L  H  A  I  Y  L  H  Q
AATGCGGGGAGAAAGCATCTGAACAAAATGGAGAGTGTGGAGGGGCTGGAGTGTGAGGAGTTACCCAACAGGGAAGAACACAGTTCAGAAGAGGGTGAGG
AGAACTAAGAGGTACCCCTTGTTGGTGACAAGGCGAAGGGAGGGCGAGGGGGCAGAAAGGGAAAGAAAGGTGGCAGCTTTCGTGCCCGAGGGACACGGGG
CGAGCGGACTCGAGGACACGTGGGTGTTCCGCACTCCTCTCCTGTAATCCTCTTAACAGCCCCGCCCACTGGCTAGTCGTTGTCCTCGTTTGACAAATGA
GGGCGCTAAGGCTAGGGGAAGTGAAGTGCCGAGCTCGAGGTCGCAGGTGGCAAGCAGTAGAGCTGGGGTGGAACGTAGCTCTGCCTTGAGTGAGGCAAGC
```

```
AAACTCCTACCAAACCACAGCCCCGCGCAGCCAGGGACAGGCTCTAAGCTGAGAGTGGGGGAGGAGATTTCAGCACCCTCTGCACCGAGCCCGGGGGCTG
GGCCTCAGGCTGCAGGGCCGGAGGGAGCCCCTCATTCTTCTCCTTTCCATTTAGCTCCATGGGCAGCAGCAGCAGGAACTGTGGGGCGAGGCTTCAGCCT
                                                                  L  H  G  Q  Q  Q  Q  E  L  W  G  E  A  S  A
CCTCCCCATCCAGGCCCTGGTCCAGCCCGCCGCAGACAGTCTCGCCGGATGAGGAGAAGCCATCCCGGTCCAGTGATGGTGAGGCGGGCAGGGTGTGCGG
S  S  P  S  R  P  W  S  S  P  P  Q  T  V  S  P  D  E  E  K  P  S  R  S  S  D
GGGACAGACCCCGGGTGAACCGCAGGTGTCACAGCACTGCCCAGCTGGTGGCTTTTCTCACCAGGCTCCAGTCCTGCTTCCAGCCCCAGAGAGCAGTGGA
                                                                              G  S  S  P  A  S  S  P  R  E  Q  W
GAGCCCAGAGGACCCGGGATCTGTTCCCTGGAGGGTTTCAGGAGCCCACAGACAGCCTGGCCAGGCCACTAGCACTGCCTGAGAGGGCTGCTCACAGCTG
R  A  Q  R  T  R  D  L  F  P  G  G  F  Q  E  P  T  D  S  L  A  R  P  L  A  L  P  E  R  A  A  H  S  W
GTCGAACTGGGGTGGAGGCGGGGCAATGAGTAGGACACTGGGTGGAGTCAGACTCGGAGGGACGGGAACAGTGCTGGGAATTTGGGGTGATGGGGACGGT
  S  N  W  G  G  G  G  A  M  S  R  T  L  G  G  V  R  L  G  G  T  G  T  V  L  G  I  W  G  D  G  D  G
CATCTCTGGAACTTCAACCCCTGGACTGCTTGTCAAGAGTTTTTCCTCCCCCAGGCTCCCTGGCTTCTTCTCCAGCCACAAGAGATTGGACGCGGTAGAG
 H  L  W  N  F  N  P  W  T  A  C  Q  E  F  F  L  P  Q  A  P  W  L  L  L  Q  P  Q  E  I  G  R  G  R
TAGAAGGGCATGCTGAGCCCTCTTTAAAGATCCCCACGGATGCTTTAGCTGGGCAGAACTTGGGAGTCAGTGAAGGACTCGCCCCAGAGAAGAGAGAAAG
V  E  G  H  A  E  P  S  L  K  I  P  T  D  A  L  A  G  Q  N  L  G  V  S  E  G  L  A  P  E  K  R  E  R
GGTAGGAGAGGAGATGCCCAAGGCATCGGGAAACATGTCTTAGAGACAGTCCTCGCGTGGAGGGGCTAAGAGACGACTGTCTGCCCTGGAGTGGTCACCT
  V  G  E  E  M  P  K  A  S  G  N  M  S  *
```

Figure S4. Multiple disruptive mutations are present in the *ATG9B* pseudogenes of toothed whales (*Odontoceti*). The sequences of ATG9B of the common bottlenose dolphin (*Tursiops truncatus*) (A) and the pygmy sperm whale (*Kogia breviceps*) (B) are shown. The nucleotide sequences of current gene predictions (March 24, 2026) and their translation were downloaded using the tool “Sequence text view” from NCBI GenBank (https://www.ncbi.nlm.nih.gov/gene/117313525 and https://www.ncbi.nlm.nih.gov/gene/131762933, last accessed on March 24, 2026). In-frame stop codons and frame-shift mutations are highlighted by blue and red shading, respectively. Note that these gene predictions of GenBank contain erroneous predictions of exon borders, and further mutations exist in both pseudogenes.

```
Acc. nr.        Start #                                                                                              End #
NC_080070.1     6352466     AGCCGCGGCCTGGCGCTCAACGTCGATCTGCTCCTCTTCCGCGGGCCCTTCTCGTTCTTCCGCGGCGGCTGGGAGCTGCCCGACGCCTAC  6352555
NC_083723.1     123256835   AGCCGCGGCCTGGCGCTCAACGTCGATCTGCTCCTCTTCCGCGGGCCCTTCTCGTTCTTCCGCGGCGGCTGGGAGCTGCCCGACGCCTAC  123256746
NC_090831.1     124318250   AGCCGCGGCCTGGCGCTCAACGTCGATCTGCTCCTCTTCCGCGGGCCCTTCTCGTTCTTCCGCGGCGGCTGGGAGCTGCCCGACGCCTAC  124318161
NC_081318.1     6403869     AGCCGCGGCCTGGCGCTCAACGTCGATCTGCTCCTCTTCCGCGGGCCCTTCTCGTTCTTCCGCGGCGGCTGGGAGCTGCCCGACGCCTAC  6403958
NC_041218.1     6217620     AGCCGCGGCCTGGCGCTCAACGGCGATCTGCTCCTCTTCCGCGGGCCCTTCTCGTTCTTCCGCGGCGGCTGGGAGCTGCCCGACGCCTAC  6217709
NC_089227.1     100026272   AGCTGCGGCCTGGCGCCCAACGTCGATCTGCTCCTCTCCCGCGGGCCCTTCTCGTTCTTCCGCGGCGGCTAGGAGCTGCCCGACGCCTAC  100026183
NW_021703776.1  99001594    AGCTGCGGCCTGGCGCCCAACGTCGATCTGCTCCTCTCCCGCGGGCCCTTCTCGTTCTTCCGCGGCGGCTAGGAGCTGCCCGACGCCTAC  99001505
NW_020174060.1  634699      AGCTGCGGCCTGGCGCCCAACGTCGATCTGCTCCTCTCCCGCGGGCCCTTCTCGTTCTTCCGCGGCGGCTAGGAGCTGCCCGACGCCTAC  634610
NW_022098064.1  6222243     AGCTGCGGCCTGGCGCCCAACGTCGATCTGCTCCTCTCCCGCGGGCCCTTCTCGTTCTTCCGCGGCGGCTAGGAGCTGCCCGACGCCTAC  6222332
NC_045771.1     6142321     AGCTGCGGCCTGGCGCCCAACGTCGATCTGCTCCTCTCTCGCGGGCCCTTCTCGTTCTTCCGCGGCGGCTAGGAGCTGCCCGACGCCTAC  6142410
NC_083322.1     6080089     AGCTGCGGCCTGGCGCCCAAC-TCGATCTGCTCCTCTCCCGCGGGCCCTTCTCGTTCTTCCGCGGCGGCTAGGAGCTGCCCGACGCCTAC  6080177
NC_064567.1     6107251     AGCTGCGGCCTGGCGCCCAAC-TCGATCTGCTCCTCTCCCGCGGGCCCTTCTCGTTCTTCCGCGGCGGCTAGGAGCTGCCCGACGCCTAC  6107339
NC_090303.1     101003396   AGCTGCGGCCTGGCGCCCAAC-TCGATCTGCTCCTCTCCCGCGGGCCCTTCTCGTTCTTCCGCGGCGGCTAGGAGCTGCCCGACGCCTAC  101003308
NC_047042.1     99230441    AGCTGCGGCCTGGCGCCCAAC-TCGATCTGCTCCTCTCCCGCGGGCCCTTCTCGTTCTTCCGCGGCGGCTAGGAGCTGCCCGACGCCTAC  99230353
NW_020837949.1  6079899     AGCTGCGGCCTGGCGCCCAAC-TCGATCTGCTCCTCTCCCGCGGGCCCTTCTCGTTCTTCCGCGGCGGCTAGGAGCTGCCCGACGCCTAC  6079987
NC_082691.1     100968513   AGCTACGGCCTGGCGCCCAAC-TCGATCTGCTCCTCTCCCGCGGGCCCTTCTCGTTCTTCCGCGGCGGCTAGGAGCTGCCCGACGCCTAC  100968425
NC_083102.1     6086703     AAATGCGGCCTGGCGCCCAAC-TCGATCTGCTCCTCTCCCGCGGGCCCTTCTCGTTCTTCCGCGGCGGCTAGGAGCTGCCCGACGCCTAC  6086791
```

Figure S5. Nucleotide sequence alignment of *ATG9B* sequence segments of cetaceans. The sequence segment corresponds to that shown in Figure 3A. However, sequences from additional species of cetaceans are included in the alignment. GenBank accession numbers (acc. nr.) and the nucleotide positions at the start and the end of the aligned sequences are shown. Red fonts indicate premature stop codons and frameshifts.

A

```
NC_045793.1       Balaenoptera musculus isolate JJ_BM4_2016_0621 chromosome 9, mBalMus1.pri.v3
NC_090831.1       Eschrichtius robustus isolate mEscRob2 chromosome 8, mEscRob2.pri
NC_080070.1       Balaenoptera acutorostrata chromosome 7, mBalAcu1.1
NC_082647.1       Balaenoptera ricei isolate mBalRic1 chromosome 9, mBalRic1.hap2
NC_083723.1       Eubalaena glacialis isolate mEubGla1 chromosome 8, mEubGla1.1.hap2.+ XY
```

B

```
NC_045793.1  6347537    CCTTCCAAGCTTTCAGGAGATGCCGCTGGCCTGATGGTGAGGCAAATGGG--CGGGGGG-  6347593
NC_090831.1  124321960  CCTTCCAAGCTTTCAGGAGATGCCGCTGGCCTGATGGTGAGGCAAATGGG--CGGGGGG-  124321904
NC_080070.1  6348752    CCTTCCAAGCTTTCAGGAGATGCCGCTGGCCTGATGGTGAGGCAAATGGG--CAGGGGG-  6348808
NC_082647.1  6362272    CCTTCCAAGCTTTCAGGAGATGCCGCTGGCCTGATGGTGAGGCAAATGGGTGGGGGTGG-  6362330
NC_083723.1  123260543  CCTTCCAAGCTTTCAGGAGATGCCGCTGGCCTGATGGTGAGGCAAATGGG--CGGGGGGG  123260486

NC_045793.1  6347594    TGGGGTGGGGTG---CGGACCAGGGGGCAGCTGGGGCGGTGGGGAGACCTGGGGCGTGGA  6347650
NC_090831.1  124321903  TGGGGTGGGGTG---TGGACCACGGGGCGGCTGGGGCGGTGGGGAGACCTGGGGCGTGGA  124321847
NC_080070.1  6348809    TGGGGTGGGGTG---CGGACCAGGGGGCAGCTGGGGCGGTGGGGAGACCTGGGGCGTGGA  6348865
NC_082647.1  6362331    GGGGGTGGGGTG---CGGACCAGGGGGCAGCTGGGGCGGTGGGGAGACCTGGGGCGTGGC  6362387
NC_083723.1  123260485  GGGGGGGGGGAGGGACGGACCAGGGGGCAGCTGGGGCGGTGGCGAGACCTGGAGCGTGGA  123260426

NC_045793.1  6347651    TCCTTGCCCCTCCTCCCCGTGCCACTGCCTCCTCT---TCCTCCTCCTCCGGGTCGGGGA  6347707
NC_090831.1  124321846  TCCGTGCCCCTCCTCCCCGTGCCACTGCCTCCTCT---TCCTCCTCCTCCGGGTCGGGGA  124321790
NC_080070.1  6348866    TCCTTGCCCCTCCTCCCCGTGCCACTGCCTCCTCT---TCCTCCTCCTCTGGGTCGGGGA  6348922
NC_082647.1  6362388    TCCTTGCCCCTCCTCCCCGTGCCACTGCCTCCTCTTCCTCCTCCTCCTCCGGGTCGGGGA  6362447
NC_083723.1  123260425  TCCGTGCCCCTCCTCCCCGGGCCACTGCCTCCTCT---TCCTCCTCCTCCGGGTTGGGGA  123260369

NC_045793.1  6347708    CCTGGGGGAGGGAGGGTCTCCATCTTCTCTCTGGCCCCTGCCCCTCACACACAAAGCACC  6347767
NC_090831.1  124321789  CCTGGGGGAGGGAGGGTCTCCATCTTCTCTCTGGCCCCTGCCCCTCACACAAAAAGCACC  124321730
NC_080070.1  6348923    CCTGGGGGAGGGAGGGTCTCCATCTTCTCTCTGGCCCCTGCCCCTCACACAAAAAGCACC  6348982
NC_082647.1  6362448    CCTGGGGGAGGGAGGGTCTCCATCTTCTCTCTGGCCCCTGCCCCTCACACAAAAAGCACC  6362507
NC_083723.1  123260368  CCTGAGGGAGGGAGGGTCTCCATCTTCTCTCTGGCCCCTGCCCCTCACACAAAAAGCACC  123260309

NC_045793.1  6347768    CCTTCCTCAGCTCCTTCTTCAGCCCCGGGGCCCCCCTTCCCAGCGGTGCAGGCCCCAGGG  6347827
NC_090831.1  124321729  CCTTCCTCAGCTCCTTCTTCAGCCCCGGGGCCCCCCTTCCCAGCGGTGCAGGCCCCAGGG  124321670
NC_080070.1  6348983    CCTTCCTCAGCTCCTTCTTCAGCCCCGGGG-ACCCCTTCCCAGCGGTGCAGGCCCCAGGG  6349041
NC_082647.1  6362508    CCTTCCTCAGCTCCTTCTTCAGCCCCGGGGCCCCCCTTCCCAGCGGTGCAGGCCCCAGGG  6362567
NC_083723.1  123260308  CCTTCCTCAGTTCCTTCTTCAGCCCCGGGGCCCCCCTACCCAGCGGTGCAGGCCCCAGGG  123260249
```

Figure S6. Alignment of nucleotide sequences from exon 1 of *ATG9B* of baleen whales. (A) GenBank accession numbers of genome sequences investigated. **(B)** Nucleotide sequence alignment. The start codon is highlighted by green shading. Indels leading to frame-shifts are highlighted by red shading. Numbers indicate nucleotide positions.

```
>primary_assembly:mBalMus1.v2:9:6347519:6356069:1
TCAGACGGTCTCCCCTTTTCCATTCCTTAGCACTGTCATACAAGCAAAACATGTAGCCTTTTTCATTTTCTTTTTTTCTTTTTGTTTTTGGTCTTAAAGTGTTTCTCTGTTGATCACTCTGTGTG
TGTGTATATAGGGAGGGGAGATAAGTTGTTTTGTTTTTTTCTGAGATAAGGTTTTAAGAAACTAAAGCTGTGTATCTTGGCCCCGTAGCCCTATAGTCCTCACTAGGATTCTTTCTGTCTTTGTT
CTTGGCTGCTGCTCTCTGGCCTCCCCGCTTTAAGTCCGCGGGGGGTCTGAAGGTGGGACCGTAGGGATCTCCTTCCATCTGCTGCTTGTCTGCTCGTGTCTCTCAAGGTACTGCCCTGCCAGGCA
TGGAGGTGGGGCCCTAAGAGGTTTGGCTGCAGGTTGCCCTGGAAGGGGTGTGAGTGGTCGGACTGGTGACTGCGAGTTGGGACGCCTGAGGCCTCCCCCAACTGAGTCACCGGGCTGTTGTTGTC
ACCGTGCTGGTTGTTGAAATCCCCGCTCAGCAACGCAGACCTCTTCAGAAATAGCAGCAGCTCCCATCCTTCCAAGCTTTCAGGAGATGCCGCTGGCCTGATGGTGAGGCAAATGGGCGGGGGGT
GGGGTGGGGTGCGGACCAGGGGGCAGCTGGGGCGGTGGGGAGACCTGGGGCGTGGATCCTTGCCCCTCCTCCCCGTGCCACTGCCTCCTCTTCCTCCTCCTCCGGGTCGGGGACCTGGGGGAGGG
AGGGTCTCCATCTTCTCTCTGGCCCCTGCCCCTCACACACAAAGCACCCCTTCCTCAGCTCCTTCTTCAGCCCCGGGGCCCCCCTTCCCAGCGGTGCAGGCCCCAGGGGCTTCTCAGCCTCGCTC
TAGTACCCTCCCCACTCCCGCTACCCCCGCCACGCAGTCCCGGCCCGCAATGACGCCCATCTCTGCTCCCCCTTCCTGGGGGTCCCACTCTGCCCCTCCCCCACGCCGATGCCCCCAGGACCCTC
CTGGGCTGCGGATAGGCCCTCTGGTCCCTGAGCAGGTTTATGAGCGGCTGGATGACTGTGATCCTGAGGGGTCCCAAGATTCAGCCCTCCATGGGGAGGAACAGCAGCCCCTGCTTCATGTGCCT
GAAGGGCTCCGAGGTGAGCGGGCGGGTGTGGGGCTGGGGAGGGCCTCAGGGCTCCAGAGTGTCCCCCGGGCTGGGCCTCTGGCAGAGGCTTGAGGGCACGGAGTCAGGGGCGTCCCAACGCTCTC
CCCTCTGTGCCCAGGCTCCTGGCACCACGTCCAGAACCTGGACAGCTTCTTCACCAAGATATCCTTTTGTGCTCTGCCTGGCAGAAGGGGCACGGGAGGCAGGGAGACAGCGGTGTAGTGGGTTG
GGGACTTGGGGGCTGAGGAGTCGGCGTCCTCAGGAAAGGCCCATCATGCCGTAGGCTTAATTCTTGACGCCCTGCCACATCTACAGCTACCACCAGAGGAGTGGCTTTGCCTGTATCCTGCTGGA
GGATGTCTTCCAGCTGGGGTGAGATGCTTGTCCCAGACCCCAAGGTCTTCCCCTTGCTTCCTCAGCAGTGCCTGCTGAGTCCGCCAGCGCCTTCCCAGGCAACCCAGGGGCTCAGCAGGATGGGT
TAATCCGGGCGGGCCTGGAGGCTGGGACAGGAGCCCTCCCAGCCCTCAGGCCTGACCACTGCACTCCCTCCTGTCCCAGACAATTCATTTTCATTGTCACCTTCACAACCTTCCTTCTTCGCTGT
GTGGATTACAGCGTTCTCTTTGCCAACCAACCAAAAAACCGAACAAGACCTGGGTCGCTCCACAGCAAAGTGACCTTGTCAGATGCTATCCTACCCTCATCCCAGTGTGCCCAGCGGTGAGTGAC
AGCTGGTGGGGGAGGTGACGGGGGAGAGCAACCGACAAAAGCCTCCCCTCCTTTTGTTCCTTGGTCTAGATCAGCCATTCTCCAAGTTTTTAGTCTCAGGCCACCTTTAATACACTTAAAATTGA
TTGAGAACCCAACTCCCAAGAGCTTTTGTTTATGAGAATCCTATCTATATTTACCTTGTTAGAGATTAAAGTTGAGAACAGCTTTAAAACCTAAGAACGTACAAACACACATTCCATCGACTGTG
ACCGTGATGTCACGTAGCCTCTGGAAAAGGCCACTGTATACTCTTGGGAGAATGCGAATGAAAATGGCAGAAAACCTTAATATTATATGAAAAGTTTTGACTTTGCAGACACTCACAAGCGTCTG
GGGACCCCCTAGGGCCCACACTTTGAGAACCACTGGCCTGGCTGGGTCCCCTCTCCGGGACCGCCATCGCTCCCCAAGCCTGGTCTCCCCTGCCATCGGGCCCCTACTTTCTCTCTTCTGTCTGC
CCCACCCGGCACAGGATCCGCTCCAGCCCCCTGCTGGTCTTCCTGCTCATCCTGGCTGCTGCCTTCTGGCTCTTCCAGCTGCTTCGCTCGGTCTGCAAACTCTTCAGCTACTGGGACATCCGGGT
GTTTTACAGGGAAGCCCTGTGCATGCCCTCAGTGAGTTGGGTGAGCGTGTCTGTGGAGGGGTGCCATGTCAGGGGGCAGAGGATGGGAAGGAGTAAGGGGGCTGTACGGACAGAAGGATCCACTG
GGAAGAGGGCAGAAGGCCTGAGTGACACCTACCCTTGGCCCCGAATAATGTTAGGAAGTGGGGTACCTCCCCTTAGAACACAAGGTCCTTACTGTTGTGGAGCGTAGTTGATGTAGATGGTTATG
GAAGATGAGGTACAGTCACGTGCTTGTGGTCCAAACAATGGGTCTTTAGAGGGAGGGCAGTCACCTGACTTTACAGAGCAGGAGGAACTGGGGTTGGGCTGGGAAGGTTAGGAGGTGGGGAGGCA
TTCCAAACCCCAGGCAGGGGAAGCAATGAGCATCTTTGGGCAGCAGTAAGGAGACTGACCCACCAGCCTGCAACTGAGTGTTCAGGCCCCCGTCGGTGAGCCCACCCCGCTTTCTGGCCCCTGTT
TGCAGGAAGCTACTTCGTCGGCCCCTTCACTGTCCCTGGTCACACTTCGTTCGCTCTTTCCTCTCCGTCTTGCTCCTGCTCCTTCTGGCTCTGCACCCAAGTTCTTTCTTTAAGGCACAGCGCAA
AGGCTACCTTCAGCAGCCGGCTCTCCTTGCTCCTCTCTGCAGTGCAGAGCCTTTTACACAGCCCTCCTGTGCAGCAAAGGGGAGAGGTTCTCGAAAGCCCACCTGAGTACAGAAGCACAAGCCAT
AGCACAGGGAAGAGCCGCGAGGGCTGGGGATGGAGGCAGGGAAGATCAGGAGCTGGACCACTGCTCCTCCAGGAGGTGCCTGACCCTTCTCTTTCTCCTTCCTTCTTTCCCCGCTTCCTTCCAGG
GGAAAGGGAGACCCTGGGATGGACTGGAGAGGCTGGCCTGGAGGGACTTGACTAAGCTGGACTGGGGAGGCTGGGTGAAACTGGGCGAAAATGAGGTTTGCAGATGAGCTCCAAGTGAGATCTGA
CAGCCTCCACCCCAGATGGGGACACGGCCATTGTTTTGGGAAGGTTAATCCGATGGAGTGGGATGGGGCGGGGTGTGTGTGTGTGTGTGTGTCTCAAAGCATGCTGAGGTAAAAGTTTGGGGGCT
TCTGGGCCCAGCGGGCTGGTACCCTAGAACAGGCAAGAGACTGCCTTGTTACCTGCAGCTGTGATGTACCTTTTATGTGTAGGGCTGTGGGCATGGGGGGGCGAGCGGTGGCCAGGAAAACCTGC
TCTTAAGGAGCTGAGCTCAGATACCTGTAGAGTTTGGTTTATGAGCTGTTCCTGGGAAGCACATGACTCACGGCCTCAGGAGGGGCAGGGCCCTGAGGAACACAGTTCAGAAGGGGATGTGGGGA
ACTAAGAGGTGCCCCTTGTTGGTGGCAAGGCAAAGGGAGGGCGAGGGGGCAGAAAGCAAAAGGTGGCAGCTTTCGTGCCCGAGAGACACTGGGTGAGGACGGCGGGGATGCATCTCTGGTCACGG
GGAGTTTGGGCATCGCCGTGGAGAGGAATCCAGGTGGCTGGCGCTCAGGCGTCCCCTCTCCGCAGGAGGAGCTCAGCTCGGTGCCCTGGGCAGAGGTGCAGTCCCGCCTCCTGGCGCTGCAGAGG
AGCGGGGGGCTGTGCGTGCAGCCGAGGCCGCTGACGGAGCTGGACGTCCACCACCGCATCCTGCGCTACACCAACTACCAGGTGGCGCTGGCCAACAAGGGCCTGCTGCCGGCCCGCTGCGCCGT
GCCCTGGGGAGGCAGCGCGGCCTTCCTCAGCCGCGGCCTGGCGCTCAACGTCGATCTGCTCCTCTTCCGCGGGCCCTTCTCCTTCTTCCGCGGCGGCTGGGAGCTGCCCGACGCCTACAAGCGCA
GCGACCGGCGGGCCGCCCTGGCCGCGCGCTGGCGGCGCACGGTGCTGCTGCTGGCGGCCGTGAACCTGGCGCTGAGCCCGCTGGTGCTGGCCTGGCAGCTGCTGCACGCCTTCTACAGCCGCGCG
GAGGTGCTGCGGCGCGAGTCCGGCGCGCTGGGGACGCGCCGCTGGTCCCGCCTGGCCCGCCTGCAGCTGCGCCACTTCAACGAGCTGCCGCACGAGCTGCGCGCGCGCCTGGCCCGCGCCTACTG
CCCCGCCGCCGCCTTCCTGCGCGCCGCCGCGCCCCCGCGCCCCTGCTCGCGCTGCTCGCCCGCCAGCTCCTCTTCTTCGCCGGCGCGCTCTTCGCCGCGCTGCTCGTGCTCACCGTCTACGACG
AGGACGTGCTCTCCGTGGAGCACGTGCTCACCGCCATGACCGCGCTCGGGATCGTGGCCACCGTGGCCAGGTGCAATGGGGGAGATATCCGGGGAGGGGGCATTCCTCTGTCCTGGGGCTGAGCC
TCCCGCGCATCCCCTACCCCCCTCACCGCTAGTGGCCCCCTAGCCCTGCATCCCAGCCGCGCATCCTCTGAACGGCAGGCCCCTTCTCCACCCCGATCCCGCCCTCTAGATCTTTCATTCCGGGT
GAGCAGGGCCAAGGGCGTCCGCCGCAAATCCTGCTGCCGGCGGCCTTGGCGCACATGCACTACCTCCCGGAGGAGACCGGCCCTGCCGGCAGGACCAGCGCTTACCGGCAGATGGCGCGGCTGTT
GCAATACCGAGCGGTGAGGGTCACGCCCGTAGCGGGGCTGCGGAGGAGGCTTGGCTGCCTGCGGTGTCGGCTTGGCTGCCTGCGGTGTCTGGGGACAAACGGGCTCAGGGCTGGCCCTCCCGGGC
GTCGGGGAATGAGTGGGTTGGAGGTTTCCTGCTTGGCGAGTTTGGTTGCCCTCAGCAGCCCTTCCCAGCCTCACCCCTGCTTCCCCAGGTCTCCCTCGCGGAGGAGCTCCTGTCGCCTCTCCTCA
CCCCACTGTTTCTGCTCTTCTGGTTTCGCCCTCGCGCCTTGGAGATTATTGACTTTTTTCGTCACTTCACTGTAGATGTGGCCGGGGTTGGGGACATCTGTTCCTTTGCCCTTATGGATGTGAAG
CGCCACGGCCACCCTCAGGTGAGAGCCTGACCCTGGCTGGGCGGGGCTGTGGTTTGGTAGGAGTTTGCTTGCCTCACTCAGAGCAGAGCTGGGTTCCACCCGCAGCTCCACTGCCTGCCACCTGT
GACCTCGAGTTAGTCACTTCACTTCCCCTTAGGGCCCTCATTTGTCAAATGCAGACAATGACTAGCCAGTGCGTGGGACTGTTAGGACGATTACGTGAGGTGACGTGTGTAGAGCCCTGCCAGAG
GTCCCAGCATGTCGTAACACCTGTAAACGGGAACGAAGACCATCAGAGAGGCCGAGAAACGTCTCGGTCAGTGGACGGGGCTGGAGTCAGGCCAGCCCTGGTATCCAGTGCGCACAGGTTTGGCA
AAACACTGAGGTCACGCCCCATCCTAGTGCTGAGTCTGGACCCAGGTGCAGTTCTGCGGCTGTGTTTTTGGGTGGTCTTGAGCTAGTCCCCATTCTTTCCAGTTCACCTGGAAAGAAGGAATGGG
GGGCTAGTGTCTCCCGTCCTGCCTACCCCAGGGGACACCCACTGCAGCTATAAAAGTGGTGTTCACGGGAGTTCCCCGGTGGCCTAGTGGTTAGGATTTGGCGCTTTCACTGCCAGGGCCCGGGT
TCCATCCCTGGTAGGGGAACTGAGATCCCACAAGCCACGTGGCACGGCCAAAAAAAAAAAAAAAAAAAAGTGCTGTTGGCGGCACCTGAAGGACTGTGGTTGCTTTCAGTGGCTCTCGGAGGGACA
GGCGGAGGCTTCACTGTCAGAGCGTGCCGAGAACGGGAAGACTGAGCTCTCACTGATGAGGTTCTCCCTGCTTCATCCACAATGGTGCCCCCCAGGCCACAGCTCCAAGTTCCTGGGGCACCTTC
GAGGCCGGGTGCAACAAGACGCGGCCGCCTGGGGCGCCAGCTCGGTTCGCAGCCCCCTCAGCCCCGGGATCCTCAGCAACTCTGCCTCCCCTCTGGTGAGTCGAAGCCTGGCCTGCCCTGCTGCC
CTCACCCCCCGGCCCCTCCCCTCCCTCTGCCTCACCTCTTGTATTAAGAGGCTTGACAGCCTGCTCTCCTGCCTTCCTTAGACCTCCGCCTCCCCTCCTGTCCTCTCTTCTGACCCTCTCTTCTC
TTGCGCACAGCAGGAGGCCTTCCCGGCCAACCTCTCGATGCAGCCCCTCGGGCCCCCCCAGGACCTGAGCCCCATAGTCCCCTGCCCAGCTGCAGCCACGGCCAGCCTCCTGGCCTCCATTTCCC
GAATGACCCAGGACTCAAGGTGAGGGGTGGGGGCCTGAGACGCAGGGAGCAGGGCTGATGGGGCTGATCCTCCAGGAGAAGGGTGCAGGGGCATGGGGGGGGGTCAGGAAGGGGAGGTGAAGCC
TGGCCCTCCCTGGGGCATGTGGGGACCGGGGGACATGTCTTTTTTTTCATCCACTGTGCCCTCTGTTCTAGCTGTGTGTCCCCAGGAGGCACTGGGGGCCAGAAGCTGGCCCAGCTCCCAGAGCT
TGCGTCTGCTGACATGAGTCTCCACGCCATTTACTTGCACCAGGTGAGTAGAGGGGTGCCAGAAAGAGAAATGCGGGGAGAAAGCAGCTGAACAAAATGGAGAGTGTGGAGGGGCTGGAGTGTAA
GGAATTACCCAACAGGAAAGAACACAGTTCAGAAGAGGGTGAGGGGAACTAAGAGGTGCCCCTTGTTGGTGACAAGGCGAAGGGAGGGCGTGGGGGCAGAAAGGGAAAGAAAGGTGGCAGGTTTT
GTGCCCGAGGGACACTGGGCGAGGACGCGAGGACACGTGGCTGTTCGCACTCCTCTCCTGTAATCCTCTTAACAGCCCCACCCACTGGCTGGTCTTTGTCCTCATTTGACAGATGAGGGCACTAA
GGGGAAGTGAAGCGACCAGCTCGAGGTCACAGGCGGCAAGCAGTGGAGCTGGGGGTGGAACGCAGCTCTGCTTTGAGTGAGGCAAGCAAACTCCTACCAAACCACAACCCCGCACAGCCAGGGAC
AGGCTCTAAGCTGAGGGTGGGGGAGGAGATTTCAGCACCCTCTGCACCGAGCCTGGGGGCTGGGCCTCAGGCTGCAGGGCCGGAGGGAGCCCCTCATTCTCCTCCTTTCCGTTTAGCTCCATCGG
CAGCAGCAGCAGGAACTGTGGGGCGAGGCTTCAGCCTCCTCCCTGTCCAGGCCCTGGTCCAGCCCGCCGCAGACACTCTCGCCGGAGGAGGAGAAGCCATCCTGGTCCAGTGGTGAGGCGCTCAG
GGTGTGCGGGGGACAGACCCCGGGTGAACCGCAGGTATCATGGCACTGCCCAGCTGATGGCTTTTCTCACCAGGCTCCAGTCCTGCTTCCAGCCCCAGACAGCAGTGGAGAACCTAGAGGACCCG
GAATCTGTTCCCTGGAGGGTTTCAGGAGCCCACAGACACCCAGAAGGAGCCTGGCCAGGCCACTAGCACCGACTGAGAGAGCTGCTGACGCTCAGTATTGTCTAACTGGGGTGGAGGCGGGGCAA
TGAGTAGGACACTGGGTGGAGTCAGACTCGGAGGGATGGGAACAATGGTGGGAATTTGGGGTGATGGGGATGGTCATCTCTGGAACCTAAACCCCTGGACTGCTTGTCAAGAGTTTTGCCTCCCC
CAGGTTCCCTGGCTTCTTCTCCAACCACAGGAGATCGGACGAGGTAGAGTGGAAGGGCACGCCGAGCCCTCTTTAAAGACCCCCATGGATGCTTTAGCTGGGCAGAACTTGGGAGTCAATGACGA
ACTCGCCCCGGAGAAGAGAGAGAGGGTAGGAGAGGAGATGCCCAAGGCATCGGGAAACATGTCTTAGAGACAGTCCTCACATGGAGAGGCTAAGAGACAACTATCTGTCCTGGAGTGGTCGTCTG
GGAAGAAGTGCCCGAGGGACCTCAAGTTGGAGGGCAGAGCCAAGATGGAGGCCAAGCTACACCTAAGGGAGCCATGAGGGACTGGACCTGAAAGGCAGTAGGTTCTTTAGACACATCTGGACTTT
AGGTTCCCGTGGGACTTGTGGAAGGGCATGGGGACCGTGAGTGGAAAGACT
```

Figure S7. *ATG9B* of the blue whale (*Balaenoptera musculus*) contains a premature stop codon in the last exon. The coding sequence homologous to *ATG9B* of land-dwelling mammals, such as the the cattle and humans, is shaded yellow. Red shading highlights a premature stop in the last exon. The sequence was downloaded from ENSEMBL, https://www.ensembl.org/Balaenoptera_musculus/Gene/Sequence?db=core;g=ENSBMSG00010013951;r=9:6348119-6355469;t=ENSBMST00010021213, last accessed on 8 February 2026. The gene is named *ATG9B*, ENSBMSG00010013951; location: Primary_assembly 9: 6,348,119-6,355,469 forward strand, mBalMus1.v2:CM020949.1.

A

```
GTCACCGTGCTGGTTGTTGAAATCCCCACTCAGCAACGCAGACCTCTTCGGAAATGGCAGCAGCTCCCATCCTTCCAAGCTTTCAGGAGATGCCGCTGGCCTGATGGTGAGGCAAATGGG
                                                                                       M  V  R  Q  M  G
CGGGGGGTGGGGTGGGGTGTGGACCACGGGGCGGCTGGGGCGGTGGGGAGACCTGGGGCGTGGATCCGTGCCCCTCCTCCCCGTGCCACTGCCTCCTCTTCCTCCTCCTCCGGGTCGGGG
  G  G  W  G  G  V  W  T  T  G  R  L  G  R  W  G  D  L  G  R  G  S  V  P  L  L  P  V  P  L  P  P  L  P  P  P  P  G  R  G
ACCTGGGGGAGGGAGGGTCTCCATCTTCTCTCTGGCCCCTGCCCCTCACACAAAAAGCACCCCTTCCTCAGCTCCTTCTTCAGCCCCGGGGCCCCCCTTCCCAGCGGTGCAGGCCCCAGG
  P  G  G  G  R  V  S  I  F  S  L  A  P  A  P  H  T  K  S  T  P  S  S  A  P  S  S  A  P  G  P  P  F  P  A  V  Q  A  P  G
GGCTTCTCAGCCTCGCTCTAGTACCCTCCCCACTCCCGCTACCCCCGCCACGCAGCCCCGGCCCGCAATGACGCCCATCTCTGCTCCCCCTTCCTGGGGGTCCCACTCTGCCCCTCCCCC
  A  S  Q  P  R  S  S  T  L  P  T  P  A  T  P  A  T  Q  P  R  P  A  M  T  P  I  S  A  P  P  S  W  G  S  H  S  A  P  P  P
ACGCCGATGCCCCCAGGACCCTCCTGGGCTGCGGATAGGCCCTCTGGTCCCTGAGCAGGTTTATGAGCGGCTGGATGACTGTGATCCTGAGGGGTCCCAAGATTCAGCCCTCCATGGGGA
  R  R  C  P  Q  D  P  P  G  L  R  I  G  P  L  V  P  E  Q  V  Y  E  R  L  D  D  C  D  P  E  G  S  Q  D  S  A  L  H  G  E
GGAACAGCAGCCCCTGCTTCATGTGCCTGAAGGGCTCCGAGGTGAGCGGGCGGGTGTGGGGCTGGGGAGGGCCTCAGGGCTCCAGAGTGTCCCCCGGGCTGGGCCTCTGGCAGAGGCTTG
  E  Q  Q  P  L  L  H  V  P  E  G  L  R
AGGGCACGGAGTCAGGGGAGTCCCAACGCTCTCCCCTCTGTGCCCAGGCTCCTGGCACCACGTCCAGAACCTGGACAGCTTCTTCACCAAGATATCCTTTTGTGCTCTGCCTGGCAGAAG
                                       G  S  W  H  H  V  Q  N  L  D  S  F  F  T  K
GGGCACGGGAGGCAGGGAGACAGGGGTGCAGTGGGTTGGGGACTTGGGGGCTGAGGAGTCGGCGTCCTCAGGAAAGGCCCATCATGCCGTAGGCTTAATTCTTGACGCCCTGCCACATCT
                                                                                                  I
ACAGCTACCACCAGAGGAGTGGCTTTGCCTGTATCCTGCTGGAGGATGTCTTCCAGCTGGGGTGAGATGCTTGTCCCAGACCCCAAGGTCTTCCCCTTGCTTCCTCAGCAGTGCCTGCTG
Y  S  Y  H  Q  R  S  G  F  A  C  I  L  L  E  D  V  F  Q  L  G
AGTCCGCCAGCGCCTTCCCAGGCAACCCAGGGGCTCAGCAGGATGGGTTAATCCGCGCAGGCCTGGAGGCTGGGGCAGGAGCCCTCCCAGCCCTCAGGCCTGACCACTGCACTCCCTCCT
GTCCCAGACAATTCATTTTCATTGTCATCTTCACAACCTTCCTTCTTCGCTGCGTGGATTACAGCGTTCTCTTTGCCAACCAACCAAAAAACCGAACAAGACCTGGGTCGCTCCACAGCA
        Q  F  I  F  I  V  I  F  T  T  F  L  L  R  C  V  D  Y  S  V  L  F  A  N  Q  P  K  N  R  T  R  P  G  S  L  H  S
AAGTGACCTTGTCAGATGCTATCGTACCCTCATCCCAGTGTGCCCAGCGGTGAGTGACAGCTGGTGGGGGAGGTGACGGGGGAGAGCAACCGACAAAAGCCTCCCCTCCTTTTGTTCCTT
K  V  T  L  S  D  A  I  V  P  S  S  Q  C  A  Q  R
GGTCTAGATCAGCCATTCTCCAAGTTTTTAGTCTCAGGCCACCTTTAATACACTTAAAATTGATTGAGAACCCAACTCCCAAGAGCTTTTGTTTATGAGAATCCTATCTATATTTACCTT
GTTAGAGATTAAAGCTGAGAACAGCTTTAAAACCTAAGAACGTACAAACACACATTCCATCGACTGTGACCGTGATGTCACGTAGCCTCTGGCAAAGGCCACTGTATACTCTTGGGAGAA
TGCGAATGAAAATGGCAGAAAACCTTAATATTATATGAAAAGTTTTGAGTTTGCAGACACTCACAAGCGTCTGGGGACCCCTAGGGCCCACACTTTGAGAACCACTGGCCCGGCTGGGTC
CCCTCTCCAGGACCGCCATCGCTCCCCAAGCCTGGTCTCCCTTGCCATGGGGCCCCTACTTTCTCTCTTCTGTCTGTCCCACCCGGCACAGGATCCGCTCCAGCCCCCTGCTGGTCTTCC
                                                                              I  R  S  S  P  L  L  V  F
TGCTCATCCTGGCTGCTGCCTTCTGGCTCTTCCAGCTGCTTCGCTCGGTCTGCAAACTCTTCAGCTACTGGGACATCCGGGTGTTTTACAGGGAGGCCCTGCGCATGCCCCCAGTGAGTT
L  L  I  L  A  A  A  F  W  L  F  Q  L  L  R  S  V  C  K  L  F  S  Y  W  D  I  R  V  F  Y  R  E  A  L  R  M  P  P
GGGTGAGCGTGTCTGTGGAGGGGTGCCATGTCAGGGGGCAGAGGATGGGAAGGAGTAAGGGGGCTGTACGGACAGAAGGATCCACTGGGAAGAGGGCAGAAGGCCTGAGTGACACCTACC
CTTGGCCCCGAATAATGTTAGGAAGTGGGGTACCTCCCCTTAGAACACAAGGTCCTTACTGTTGTGGAGCGTAGTTGATGTAGATGGTTATGGAAGATGAGGTACAGTCACGTGCTTGTG
GTCCAAACAATGGGTCTTTAGAGGGAGGGCAGTCACCTGACTTTACAGAGCAGGAGGAACTGGGGTTGGGCTGGGAAGGTTAGGAGGTGGGGAGGCATTCCAAACCCCAGGCAGGGGAAG
CAATGAGCATCTTTGGACAGCAGTAAGGAGACTGACCCACCAGCCTGCAACTGAGTGTTCAGGCCCCCGTCGGTGAGCCCACCCCGCTTTCTGGCCCGTTTGCAGGAAGCTACTTCGTCG
GCCCCTTCACTGTCCCTGGTCACGCTTCGCTCTTTCCTCTCCGTCTTGCTCCTGCTCCTTCTGGCTCTGCACGCAAGTTCTTTCTTTAAGGCACAGCGCAAAGGCTACCTTCAGCAGCCG
GCTCTCCTTGCTCCTCTCAGCAGTGCAGAGCCTTTTACATAGCCCTCCTGTGCAGCAAAGGGGAGAGGTTCTCAAAAGCCCACCTGAGTACGGAAGCACAAGCCGTAGCACAGGGAAGAG
CCGCGAGGGCTGGGGATGGAGGCAGGGAAGATCAAGAGCTGGACCACTGCTCCTCCAGGAGGTGCCTGACCCTTCTCTTTCTCCTTCCTTCTTTCCCCGCTTCCTTCCAGGGGAAAGGGA
GACCCTGGGATGGACTGGAGAGGCTGGCCTGGAGGGACTTGACTAAGCTGGACTGGGGAGGCTGGGTGAAACTGGGCGAAAATGAGGTTTGCAGATGAGCTCCAAGTGAGATCTGACAGC
CTCCACCCCAGATGGGGACACGGCCATTGTCTTGGGAAGGTTAACCCGATGGAGTGGGATGGGGCGGGGGTGTGTGTGTGTGTGTCTCAAAGCATGCTGAGGTAAGAGTTTGGGGGCTTC
TGGGCCCAGCGGGCTGGTACCCTAGGAACGGGCAAGAGACTGCCTTGTTACCTGCAGCTGTGATGTACCTTGTATGTGTAGGGCTGTGGGCATGCGGGGGGGGGCGGGCGAGCGGTGGCC
AGGAAAACCTGCTCTTAAGGAGCTGAGCTCAGATACCTGTAGAGTTTGGTTTATGAGCTGTTCCTGGGAAGCACATGACTCACGGCCTCAGGAGGGGCAGGGCCCTGAGGAACACAGTTC
AGAAGGGGATGTGGGGAACTAAGAGGTGCCCCTTGTTGGTGGCAAGGCGAAAGGAGGGCGAGGGGGCAGAAAGCAAAAGGTGGCAGCTTTCGTGCCCGAGAGACACTGGGTGAGGACGGC
GGGGATGCGTCTCTGGTCACGGGGAGTTTGGGCATCGCCGTGGAGAGGAATCCAGGTGGCTGGCGCTCAGGCGTCCCCTCTCCGCAGGAGGAGCTCAGCTCGGTGCCCTGGGCAGAGGTG
                                                                          E  E  L  S  S  V  P  W  A  E  V
CAGTCCCGCCTCCTGGCGCTGCAGAGGAGCGGGGGGCTGTGCGTGCAGCCGAGGCCGCTGACGGAGCTGGACGTCCACCACCGCATCCTGCGCTACACCAACTACCAGGTGGCGCTGGCC
 Q  S  R  L  L  A  L  Q  R  S  G  G  L  C  V  Q  P  R  P  L  T  E  L  D  V  H  H  R  I  L  R  Y  T  N  Y  Q  V  A  L  A
AACAAGGGCCTGCTGCCGGCCCGCTGCGCCGTGCCCTGGGGAGGCAGCGCGGCCTTCCTCAGCCGCGGCCTGGCGCTCAACGTCGATCTGCTCCTCTTCCGCGGGCCCTTCTCGTTCTTC
 N  K  G  L  L  P  A  R  C  A  V  P  W  G  G  S  A  A  F  L  S  R  G  L  A  L  N  V  D  L  L  L  F  R  G  P  F  S  F  F
CGCGGCGGCTGGGAGCTGCCCGACGCCTACAAGCGCAGCGACCGGCGGGCCGCCCTGGCCGCGCGCTGGCGGCGCACGGTGCTGCTGCTGGCGGCCGTGAACCTGGCGCTGAGCCCGCTG
 R  G  G  W  E  L  P  D  A  Y  K  R  S  D  R  R  A  A  L  A  A  R  W  R  R  T  V  L  L  L  A  A  V  N  L  A  L  S  P  L
GTGCTGGCCTGGCAGCTGCTGCACGCGTTTTACAGCCGCGCGGAGGTGCTGCGGCGCGAGCCCGGCGCGCTGGGGACGCGCCGCTGGTCCCGCCTGGCCCGCCTGCAGCTGCGCCACTTC
 V  L  A  W  Q  L  L  H  A  F  Y  S  R  A  E  V  L  R  R  E  P  G  A  L  G  T  R  R  W  S  R  L  A  R  L  Q  L  R  H  F
AACGAGCTGCCGCACGAGCTGCGCGCGCGCCTGGCCCGCGCCTACTGCCCCGCCGCCGCCTTCCTGCGCGCCGCCGCGCCCCCCGCGCCCCTGCTCGCGCTGCTCGCCCGCCAGCTCCTC
 N  E  L  P  H  E  L  R  A  R  L  A  R  A  Y  C  P  A  A  A  F  L  R  A  A  A  P  P  A  P  L  L  A  L  L  A  R  Q  L  L
TTCTTCGCCGGCGCGCTCTTCGCCGCGCTGCTCGTGCTCACCGTCTACGACGAGGACGTGCTCTCCGTGGAGCACGTGCTCACCGCCATGACCGCGCTCGGGATCGTGGCCACCGTGGCC
 F  F  A  G  A  L  F  A  A  L  L  V  L  T  V  Y  D  E  D  V  L  S  V  E  H  V  L  T  A  M  T  A  L  G  I  V  A  T  V  A
AGGTGCAATGGGGGAGATATCCGGGGAGGGGGCATTCCTCTGTCCTGGGGCTGAGCCTCCCGCGCATCCCCTACCCCTCCCACCGCTAGTGGCCCCCGAGCCCTGCATCCCAGCCGCGCA
 R
TCCTCTGACCGGCAGGCCCCTTCTCCACCCCGATCCCGCCCTCTAGGTCTTTCATTCCGGGTGAGCAGGGCCAAGGTCGTCCGCCGCAAATCCTGCTGCCGGCGGCCTTGGCGCACATGC
                                        S  F  I  P  G  E  Q  G  Q  G  R  P  P  Q  I  L  L  P  A  A  L  A  H  M
ACTACCTCCCGGAGGAGACCGGCCCTGCCGGCAGGACCAGCGCTTACCGGCAGATGGCGCGGCTGTTGCAATACCGAGCAGTGAGGGTCACGCCCGTAGCGGGGCTGCGGAGGAGGCTTG
H  Y  L  P  E  E  T  G  P  A  G  R  T  S  A  Y  R  Q  M  A  R  L  L  Q  Y  R  A
GCTGCCCGCGGTGTCTGGGGACAAACGGGCTCAGGGCTGGCCCTCCCGGGCGTCAGGGAATGAGTGGGTTGGAGGTTTCCTGCTTGGCGAGTTTGGTTGCCCTCAGCAGCCCTTCCCAGC
CTCACCCCTGCTTCCCCAGGTCTCCCTCGCGGAGGAGCTCCTGTCGCCTCTCCTCACCCCACTGTTTCTGCTCTTCTGGTTTCGCCCTCGCGCCTTGGAGATTATTGACTTTTTTCGTCA
                 V  S  L  A  E  E  L  L  S  P  L  L  T  P  L  F  L  L  F  W  F  R  P  R  A  L  E  I  I  D  F  F  R  H
CTTCACTGTAGATGTGGCCGGGGTTGGGGACATCTGTTCCTTTGCCCTTATGGATGTGAAGCGCCACGGCCACCCTCAGGTGAGAGCCTGACCCTGGCTGAGCGGGGCTGTGGTTTGGTA
  F  T  V  D  V  A  G  V  G  D  I  C  S  F  A  L  M  D  V  K  R  H  G  H  P  Q
GGAGTTTGCTTGCCTCACTCAGAGCAGAGCTGGGTTCCACCCCCAGCTCCACTGCTTGCCACCTGTGACCTCGAGTTAGTCACTTCACTTCCCCTTAGGGCCCTCATTTGTCAAATGCAG
ACAATGACTAGCCAGTGCGTGGGACTGTTAGGACGATTACGTGAGGTGACGTGTGTAGAGCCCTGCCAGAGGTCCCAGCATGTCGTAAACACCTGTAAACGGGAACGAAGACCATCAGAG
AGGCCGAGAAACGTCTCGGTCAGTGGACGGGGCTGGAGTCAGGCCAGCCCTGGTATCCAGTGCGCACAGGTTTGGCAAAACACTGAGGTCACGCCCCATCCTAGTGCTGAGTCTGGACCC
AGGTGCAGTTCTGCGGCTGTGTTTTTGGGTGGTCTTGAGCTAGTCCCCATTCTTTCCAGTTCACCTGGAAAGAAGGAATGGGGGGCTAGTGTCCCCCGTCCTGCCTACCCCAGGGGACAC
CCACTGCAGCTATAAAAGTGGTGTTCACGGGAGTTCCCCGGTGGCCTAGTGGTTAGGATTTGGCGCTTTCACTGCTAGGGCCCGGGTTCCATCCCTGGTAGGGGAACTGAGATCCCACAA
GCCACGTGGCACGGCCAAAAAAAAAAAAAAAAAAAAAAAGTGCTGTTGGCGGCACCTGAAGGACTGTGGTTGCTTTCAGTGGCTCTCGGAGGGACAGACGGAGGCTTCACTGTCAGAGCGT
                                                                  W  L  S  E  G  Q  T  E  A  S  L  S  E  R
GCCGAGGACGGGAAGACTGAGCTCTCACTGATGAGGTTCTCCCTGCTTCATCCACAATGGTGCCCCCCAGGCCACAGCTCCAAGTTCCTGGGGCACCTTCGAGGCCGGGTGCAACAAGAC
 A  E  D  G  K  T  E  L  S  L  M  R  F  S  L  L  H  P  Q  W  C  P  P  G  H  S  S  K  F  L  G  H  L  R  G  R  V  Q  Q  D
GCGGCCGCCTGGGACGCCAGCTCGGTTCGCAGCCCCCCAGCCCCGGGATCCTCAGCAACTCTGCCTCCCCTCTGGTGAGTCGAAGCCTGGCCTGCCCTGCTGCCCTCACCCCCTGGCCC
 A  A  A  W  D  A  S  S  V  R  S  P  P  S  P  G  I  L  S  N  S  A  S  P  L
CTCCCCTCCCTCTGCCTCACCTCTTGTATTAAGAGGCTTGACAGCCTGGTCTCCTGCCTTCCTTAGACCTCCGCCTCCCCTCCTGTCCTCTCTTCTGACCCTCTCTTCTCTTGCACGCAG
CAGGAGGCCTTCCCGGCCAACCTCTCGATGCAGCCCCTCGGGCCCCCCCAGGACCTGAGCCCCATAGTCCCCTGCCCAGCTGCAGCCACGGCCAGCCTCCTGGCCTCCATTTCCCGAATG
 Q  E  A  F  P  A  N  L  S  M  Q  P  L  G  P  P  Q  D  L  S  P  I  V  P  C  P  A  A  A  T  A  S  L  L  A  S  I  S  R  M
ACCCAGGACTCAAGGTGAGGGGTGGGGGCCTGAGACGCAGGGAGCAGGGCTGATGGGGCTGATCCTCCAGGAGAAGGGTGCAGGGGCATGGGGGGGGGTGATCAGGAAGGGGAGGTGAAG
 T  Q  D  S  S
CCTGGCCCTCCCTGGGGCATGTGGGGACCGGGGGACATGTCTTTTTTTTCATCCACTGTGCCCTCTGTTCTAGCTGTGTGTCCCCAGGAGGCACTGGGGGCCAGAAGCTGGCCCAGCTCC
                                                               C  V  S  P  G  G  T  G  G  Q  K  L  A  Q  L
CAGAGGTTGCGTCTGCTGACATGAGTCTCCACGCCATTTACTTGCACCAGGTGAGTAGAGGGGTGCCAGAAAGAGAAATGCGGGGAGAAAGCAGCTGAACAAAATGGAGAGTGTGGAGGG
P  E  V  A  S  A  D  M  S  L  H  A  I  Y  L  H  Q
GCTGGAGTGTAAGGAATTACCCAACAGGAAAGAACACAGTTCAGAAGAGGGTGAGGGGAACTAAGAGGTGCCCCTTGTTGGTGACAAGGCGAAGGGAGGGCGTGGGGGCAGAAAGGGAAA
GAAAGGTGGCAGGTTTTGTGCCCGAGGGACACTGGGCGAGGACGCGAGGACACGTGGGTGTTCGCACTCCTCTCCTGTAATCCTCTTAACAGCCCCACCCACTGGCTAGTCTTTGTCCTC
ATTTGACAGATGAGGGCACTAAGGGGAAGTGAAGCGACCAGCTCGAGGTCACAGGTGGCAAGCAGTGGAGCTGGGGGTGGAACGCAGCTCTGCTTTGAGTGAGGCAAGCAAACTCCTACC
AAACCACAACCCCGCACAGCCAGGGACAGGCTCTAAGCTGAGGGTGGGGGAGGAGATTTCAGCACCCTCTGCACCGAGCCTGGGGGCTGGGCCTCAGGCTGCAGGGCCGGAGGGAGCCCC
TCATTCTCCTCCTTTCCGTTTAGCTCCATCGGCAGCAGCAGCAGGAACTGTGGGGCGAGGCTTCAGCCTCCTCCCTGTCCAGGCCCTGGTCCAGCCTGCTGCAGACACTCTCGCCGGAGG
                    L  H  R  Q  Q  Q  Q  E  L  W  G  E  A  S  A  S  S  L  S  R  P  W  S  S  L  L  Q  T  L  S  P  E
AGGAGAAGCCATCCTGGTCCAGTGGTGAGGCGCTCAGGGTGTGCGGGGGACAGACCACGGGTGAACCGCAGGTATCATGGCACTGCCCAGCTGATGGCTTTTCTCACCGGGCTCCAGTCC
E  E  K  P  S  W  S  S                                                                 S  P  G  S  S  P
TGCTTCCAGCCCCAGACAGCAGTGGAGAACCCAGAGGACCCGGAATCTGTTCCCTGGAGGGTTTCAGGAGCCCACAGACACCCAGAAGGAGCCTGGCCAGGCCACTAGCACCGACTGAGA
  A  S  S  P  R  Q  Q  W  R  T  Q  R  T  N  L  F  P  G  G  F  Q  E  P  T  D  T  Q  K  E  P  G  Q  A  T  S  T  D  *
```

B

```
                              W  S  S  D                                                                                     G  S  S
Hippopotamus amphibius kiboko TGGTCCAGTGATGGTGAGGCGGTGGGGGCGTGCAGGGGACAGACCCTGGGTGAACCACAGGCATCTTGGCACTGCCCAGCTAAAGGCTTTTCTCACCAGGCTCCAGT
Eubalaena glacialis           TGGTCCAGTGATGGTGAGGCGGTCAGGGTGTGTGGGGACAGACCCCGGGTGAACCGCAGGTATCATGGCACTGCCCAGCTGATGGCTTTTCTCACCAGGCTCCAGT
Balaenoptera musculus         TGGTCCAGT---GGTGAGGCGCTCAGGGTGTGCGGGGACAGACCCCGGGTGAACCGCAGGTATCATGGCACTGCCCAGCTGATGGCTTTTCTCACCAGGCTCCAGT
Eschrichtius robustus         TGGTCCAGT---GGTGAGGCGCTCAGGGTGTGCGGGGACAGACCACGGGTGAACCGCAGGTATCATGGCACTGCCCAGCTGATGGCTTTTCTCACCGGGCTCCAGT
                              W  S  S                                                                                        G  S  S
```

Figure S8. *ATG9B* of the grey whale (*Eschrichtius robustus*). (A) Nucleotide sequence and translation of coding sequence downloaded from GenBank (https://www.ncbi.nlm.nih.gov/gene/?term=137768632, last accessed on 8 February 2026). Erroneous predictions of amino acid residues were corrected (red fonts). **(B)** The nucleotide sequence of the last intron and the flanking exonic regions of *ATG9B* genes were aligned. Consensus splice motifs (GT, splice donor site; AG, splice acceptor site) are underlined. Coding sequences are shaded yellow. Red shading highlights a mutation that likely impairs splicing at this site in the grey whale.

Table S1. Accession numbers of genome sequences

Species	Phylogenetic clade	Genomes sequence assembly name	GenBank accession number
Balaenoptera acutorostrata (minke whale)	Mysticeti	mBalAcu1.1	GCF_949987535.1
Balaenoptera musculus (blue whale)	Mysticeti	mBalMus1.pri.v3	GCF_009873245.2
Balaenoptera ricei (Rice's whale)	Mysticeti	mBalRic1.hap2	GCF_028023285.1
Eschrichtius robustus (grey whale)	Mysticeti	mEscRob2.pri	GCF_028021215.1
Eubalaena glacialis (North Atlantic right whale)	Mysticeti	mEubGla1.1.hap2.+ XY	GCF_028564815.1
Physeter macrocephalus (sperm whale)	Odontoceti	ASM283717v2	GCF_002837175.3
Kogia breviceps (pygmy sperm whale)	Odontoceti	mKogBre1 haplotype 1	GCF_026419965.1
Mesoplodon densirostris (Blainville's beaked whale)	Odontoceti	mMesDen1 primary haplotype	GCF_025265405.1
Phocoena phocoena (harbor porpoise)	Odontoceti	mPhoPho1.1	GCF_963924675.1
Phocoena sinus (vaquita)	Odontoceti	mPhoSin1.pri	GCF_008692025.1
Neophocaena asiaeorientalis asiaeorientalis (Yangtze finless porpoise)	Odontoceti	Neophocaena_asiaeorientalis_V1	GCF_003031525.1
Monodon monoceros (narwhal)	Odontoceti	NGI_Narwhal_1	GCF_005190385.1
Delphinapterus leucas (beluga whale)	Odontoceti	ASM228892v3	GCF_002288925.2
Globicephala melas (long-finned pilot whale)	Odontoceti	mGloMel1.2	GCF_963455315.2
Orcinus orca (orca, killer whale)	Odontoceti	mOrcOrc1.1	GCF_937001465.1
Pseudorca crassidens (false killer whale)	Odontoceti	mPseCra1.hap1	GCF_039906515.1
Sagmatias obliquidens (Pacific white-sided dolphin)	Odontoceti	ASM367639v1	GCF_003676395.1
Delphinus delphis (saddleback dolphin)	Odontoceti	mDelDel1.2	GCF_949987515.2
Lagenorhynchus albirostris (white-beaked dolphin)	Odontoceti	mLagAlb1.1	GCF_949774975.1
Tursiops truncatus (common bottlenose dolphin)	Odontoceti	mTurTru1.mat.Y	GCF_011762595.2
Alligator mississippiensis (American alligator)	Crocodylia	rAllMis1	GCF_030867095.1
Alligator sinensis (Chinese alligator)	Crocodylia	ASM45574v1	GCF_000455745.1
Chrysemys picta bellii (western painted turtle)	Testudines	ASM1138683v2	GCF_011386835.1
Chelonia mydas (green sea turtle)	Testudines	rCheMyd1.pri.v2	GCF_015237465.2
Latimeria chalumnae (coelacanth)	Coelacanthiformes	fLatCha1.pri	GCF_037176945.1
